# Structural Transformations of Metal–Organic Cages through Tetrazine-Alkene Reactivity

**DOI:** 10.1021/jacs.4c08591

**Published:** 2024-09-05

**Authors:** Martin R. Black, Soumalya Bhattacharyya, Stephen P. Argent, Ben S. Pilgrim

**Affiliations:** School of Chemistry, University of Nottingham, University Park, Nottingham NG7 2RD, U.K.

## Abstract

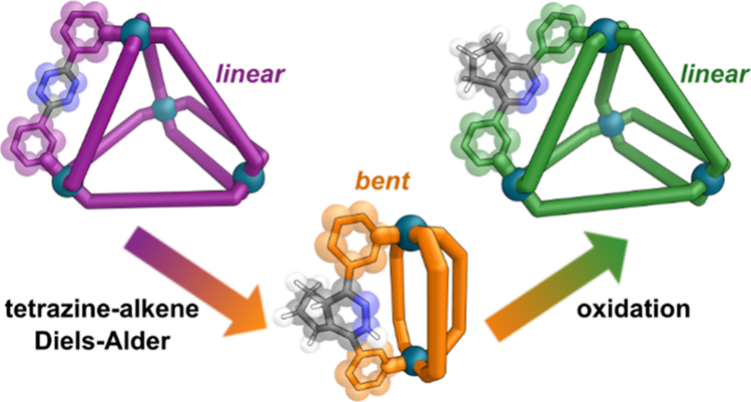

The assembly of metal–organic cages is governed by metal ion coordination preferences and the geometries of the typically rigid and planar precursor ligands. Pd*_n_*L_2*n*_ cages are among the most structurally diverse, with subtle differences in the metal–ligand coordination vectors resulting in drastically different assemblies, however almost all rely on rigid aromatic linkers to avoid the formation of intractable mixtures. Here we exploit the inverse electron-demand Diels–Alder (IEDDA) reaction between tetrazine linker groups and alkene reagents to trigger structural changes induced by post-assembly modification. The structure of the 1,4-dihydropyridazine produced by IEDDA (often an afterthought in click chemistry) is crucial; its two sp^3^ centers increase flexibility and nonplanarity, drastically changing the range of accessible coordination vectors. This triggers an initial Pd_4_L_8_ tetrahedral cage to transform into different Pd_2_L_4_ lantern cages, with both the transformation extent (thermodynamics) and rate (kinetics) dependent on the alkene dienophile selected. With cyclopentene, the unsymmetrical 1,4-dihydropyridazine ligands undergo integrative sorting in the solid state, with both head-to-tail orientation and enantiomer selection, leading to a single isomer from the 39 possible. This preference is rationalized through entropy, symmetry, and hydrogen bonding. Subsequent oxidation of the 1,4-dihydropyridazine to the aromatic pyridazine rigidifies the ligands, restoring planarity. The oxidized ligands no longer fit in the lantern structure, inducing further structural transformations into Pd_4_L_8_ tetrahedra and Pd_3_L_6_ double-walled triangles. The concept of controllable addition of limited additional flexibility and then its removal through well-defined reactivity we envisage being of great interest for structural transformations of any class of supramolecular architecture.

## Introduction

**Figure 1:**
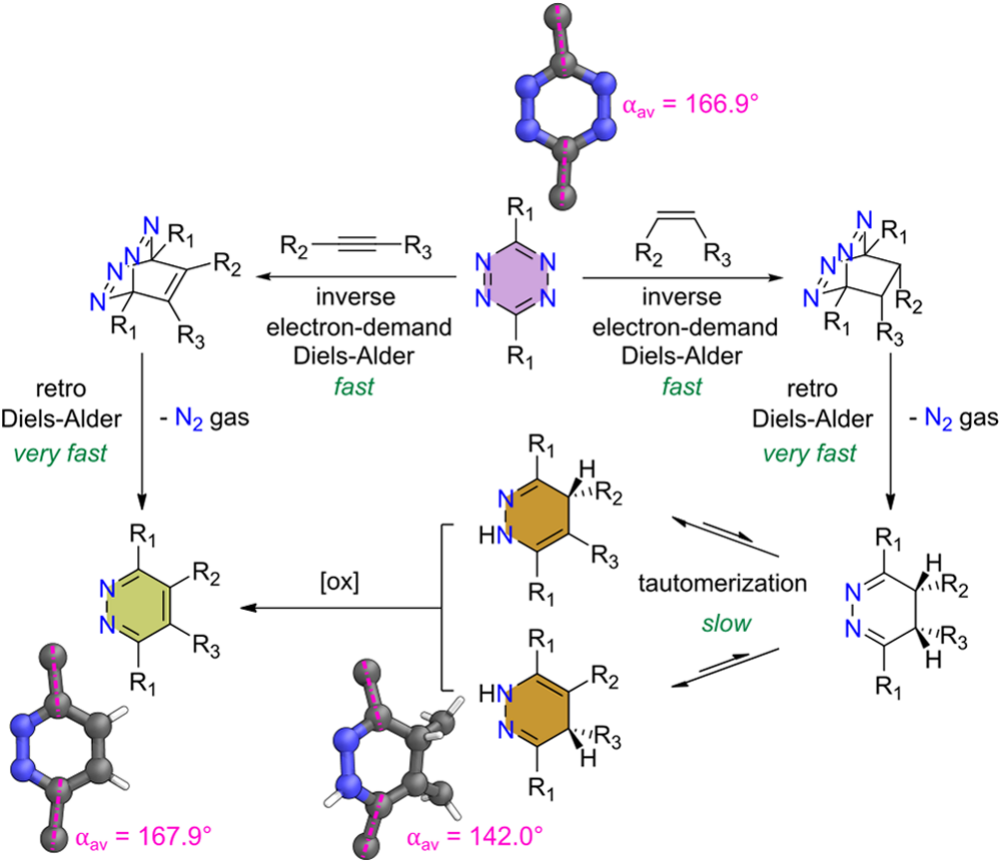
Mechanism of the inverse electron-demand Diels–Alder (IEDDA) reaction of tetrazine with alkynes and alkenes. Inset, extracted representative geometries of tetrazine, 1,4-dihydropyridazine, and pyridazine rings from single crystal X-ray diffraction structures obtained in this work, with the angle α_av_ between adjoining C–C bonds indicated. Color: C = gray, N = blue, H = white.

The inverse electron-demand Diels–Alder (IEDDA) reaction between tetrazines and alkenes/alkynes has recently emerged as a leading reaction in click and bioorthogonal chemistry,^1^ due to fast reaction kinetics, high selectivity, and biocompatibility. Focus has naturally been on optimizing the first IEDDA step to be as fast as possible, as this accomplishes conjugation, with second order rate constants up to 10^6^ M^–1^ s^–1^ having been achieved. After the initial IEDDA (Figure 1), a second retro-Diels–Alder step occurs rapidly to relieve strain and liberate nitrogen gas. However, after this the final reaction outcome depends on the components. Tetrazine plus alkyne gives the aromatic pyridazine directly, whereas tetrazine plus alkene gives a 4,5-dihydropyridazine intermediate, which undergoes a slower tautomerization to a 1,4-dihydropyridazine,^2^ which can then be reoxidized to the aromatic pyridazine. While the resulting structures of the IEDDA products are of little consequence in conjugation chemistry, we reasoned that the greater flexibility and nonplanarity of the ring in this dihydro oxidation level could be exploited to drive structural transformations of metal–organic cage architectures.

Tetrazine IEDDA chemistry is now widely employed for labeling.^3^ Applications which use a feature of this chemistry other than its high conjugation efficiency are less common, but include the ability to turn on fluorescence,^4^ and elimination from the dihydropyridazine intermediate to trigger the release of a fragment of interest,^5^ for example in biopolymer-targeted drug delivery.^6^ Within (metallo)supramolecular chemistry, the IEDDA reaction has been used previously for the covalent post-assembly modification (PAM) of supramolecular structures,^7^ with the mild reaction conditions and quantitative nature of the reaction being essential to not disrupt the dynamic linkages in these systems. In most reported cases on the IEDDA modification of metal–organic frameworks (MOFs),^8^ metal–organic cages,^9^ or organic macrocycles,^10^ there were no major structural changes arising from the reaction, although some changes in material rigity,^11^ or host–guest binding constants have been reported.^12^ The reaction has also recently been employed to drive the transformation of trefoil knots to Solomon links,^13^ and the unlinking of Borromean rings.^14^

In previous work on metal–organic cages, dienophiles were always chosen so that the planar aromatic pyridazine was reached directly, and the rare examples of structural change were driven through the steric bulk of the conjugated substituents or other factors such as anion templation.^15^ This is in part due to the limited literature on the steps after the initial Diels–Alder in the IEDDA reaction sequence. The rate of tautomerization of the 4,5-dihydropyridazine to the 1,4-dihydropyridazine varies depending on the system, but is reported to occur via the intermediate hemiaminal and can take many hours.^2^ The 1,4-dihydropyridazines can be subsequently oxidized to planar aromatic pyridazines. Efficient oxidants reported include nitrous reagents (e.g., isoamyl nitrite, NaNO_2_), hydrogen peroxide, DDQ, and PhI(OAc)_2_.^16^ However even in the absence of an added oxidant, slow oxidation from the air can still occur, taking from days to weeks.^17^

Palladium-based assemblies of formula Pd*_n_*L_2*n*_ are among the most widely studied metal–organic cages and are typically constructed from the combination of Pd(II) salts and ditopic monodentate donor ligands L (characteristically with pyridine nitrogen atoms as the donors).^18^ These structures have seen applications in catalysis,^19^ medicinal chemistry (including *cis*-platin delivery^20^ and in anticancer^21^ and cytotoxic agents^22^), and have been hierarchically assembled into functional materials including vesicles^23^ and gels.^24^ A wide variety of structures are accessible including Pd_2_L_4_ lanterns,^25^ Pd_4_L_8_ tetrahedra,^26^ topologically interlocked Pd_4_L_8_^27^ and Pd_8_L_16_ architectures,^23^ Pd_6_L_12_ octahedra,^28^ and larger structures up to Pd_48_L_96_.^29^ Structural rigidity of the ligand is generally crucial to favor a shape-persistent, well-defined architecture, with flexible ligands leading to product mixtures or limiting assemblies to those of low nuclearity. Hence, the typical ligand design features two pyridine coordinating groups linked by rigid and planar sp^2^ or sp hybridized spacers. However, slight alteration of the average angle, Θ_av_, between metal–ligand coordination vectors can have profound influence on the structure that forms. Both tetrazine and pyridazine are aromatic heterocycles, and hence the ring planarity forces the C–C bonds to adjoining substituents at the 3 and 6-positions to be orientated close to antiparallel to each other (Figure 1), with the average angle, α_av_ = 166.9 ± 2.0° for tetrazine and 167.9 ± 2.2° for pyridazine in this work (see Section S8.1 and S8.2 in the Supporting Information). By contrast the two sp^3^ centers in a 1,4-dihydropyridazine ring mean the ring is no longer planar, and this angle significantly reduces, with α_av_ = 142.0 ± 2.5° (Section S8.4 in the Supporting Information), which we exploit to enable structural transformations through their influence on the average angle, Θ_av_, between metal–ligand coordination vectors.

Herein we describe a structural transformation sequence of a tetrazine-edged Pd_4_L_8_ tetrahedral cage. Through IEDDA with alkene dienophiles and subsequent oxidation, we have observed an initial transformation to Pd_2_L_4_ lanterns upon IEDDA reaction. By the controlled addition of a limited amount of extra flexibility, intermediate 1,4-dihydropyridazine structures possessing a nonplanar ligand conformation have been accessed for the first time. Removal of this flexibility through reoxidation of the ring to a planar aromatic pyridazine induces a further transformation to Pd_4_L_8_ tetrahedra and Pd_3_L_6_ triangles. Unlike previous transformation sequences between thermodynamic equilibria structures, the intermediate lanterns are transient under normal atmospheric conditions, with control demonstrated over their rate of formation with the chosen alkene, and their rate of removal with the chosen oxidant.

## Results and Discussion

**Figure 2:**
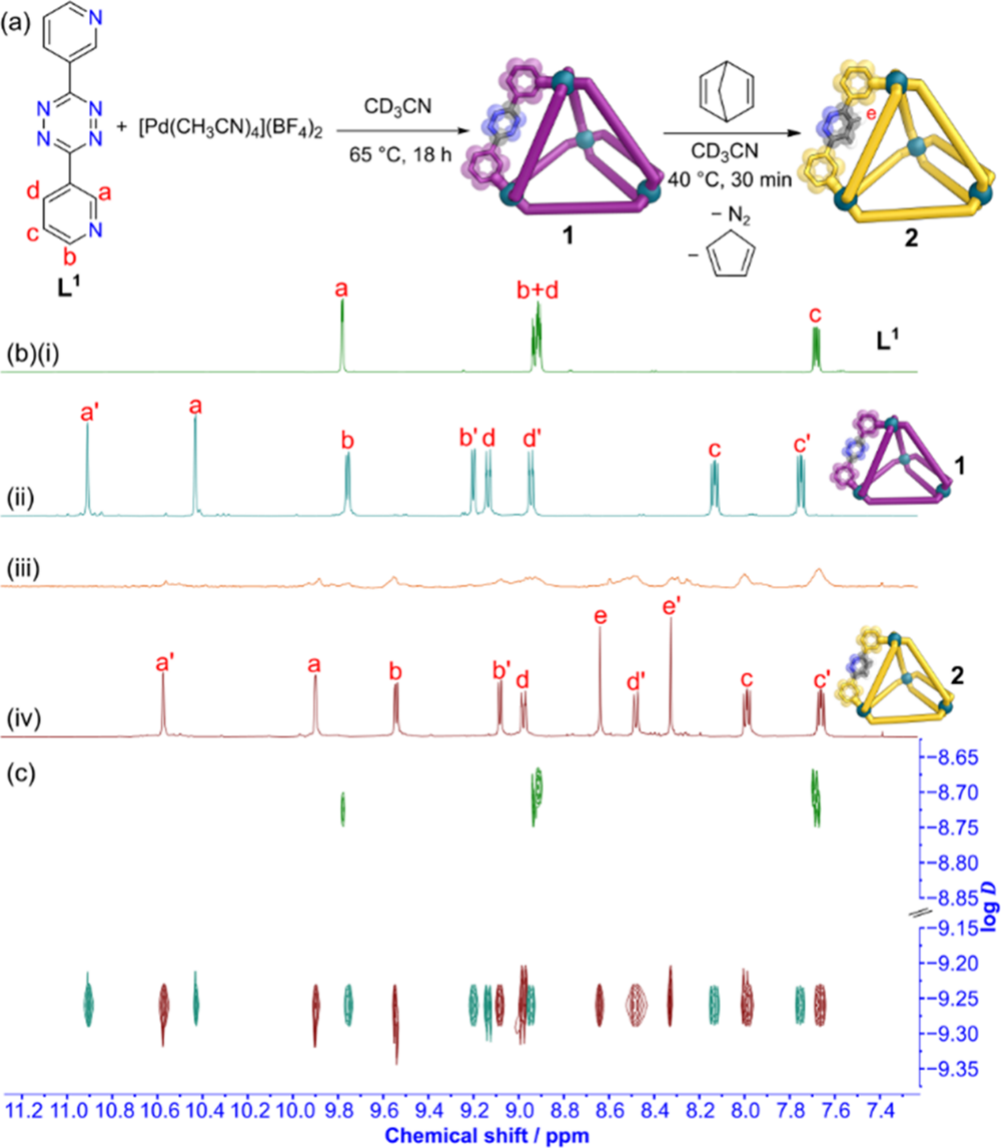
(a) Synthesis of cage **1** and post-assembly modification to cage **2**. (b) Stacked partial ^1^H NMR spectra (CD_3_CN, 500 MHz, 298 K) of: (i) ligand **L**^**1**^ (green); (ii) cage **1** (blue); (iii) cage **1** and NBD after 5 min at 313 K (spectrum also ran at 313 K) (orange); (iv) product cage **2** (maroon) with new pyridazine signals e and e′ after 30 min. (c) Overlaid DOSY NMR spectra of **L**^**1**^ (green), cage **1** (blue) and cage **2** (maroon).

Self-assembly of ditopic tetrazine ligand **L**^**1**^ with [Pd(CH_3_CN)_4_](BF_4_)_2_ occurred over 18 h at 65 °C in CD_3_CN, resulting in a clear pink colored solution. ^1^H and ^13^C NMR spectroscopic studies revealed only a single metallosupramolecular species was present which possessed two signals for each ligand environment (Figures 2a and S18), consistent with the formation of a tetrahedral structure analogous to the behavior of the benzene-centered ligand previously reported.^30^ A single diffusion coefficient for all proton signals was observed in the DOSY NMR spectrum, with log *D* = −9.26, indicative of an assembly of large size. For comparison, ligand **L**^**1**^ had a lower diffusion coefficient, with log *D* = −8.71 (Figure 2c).

**Figure 3:**
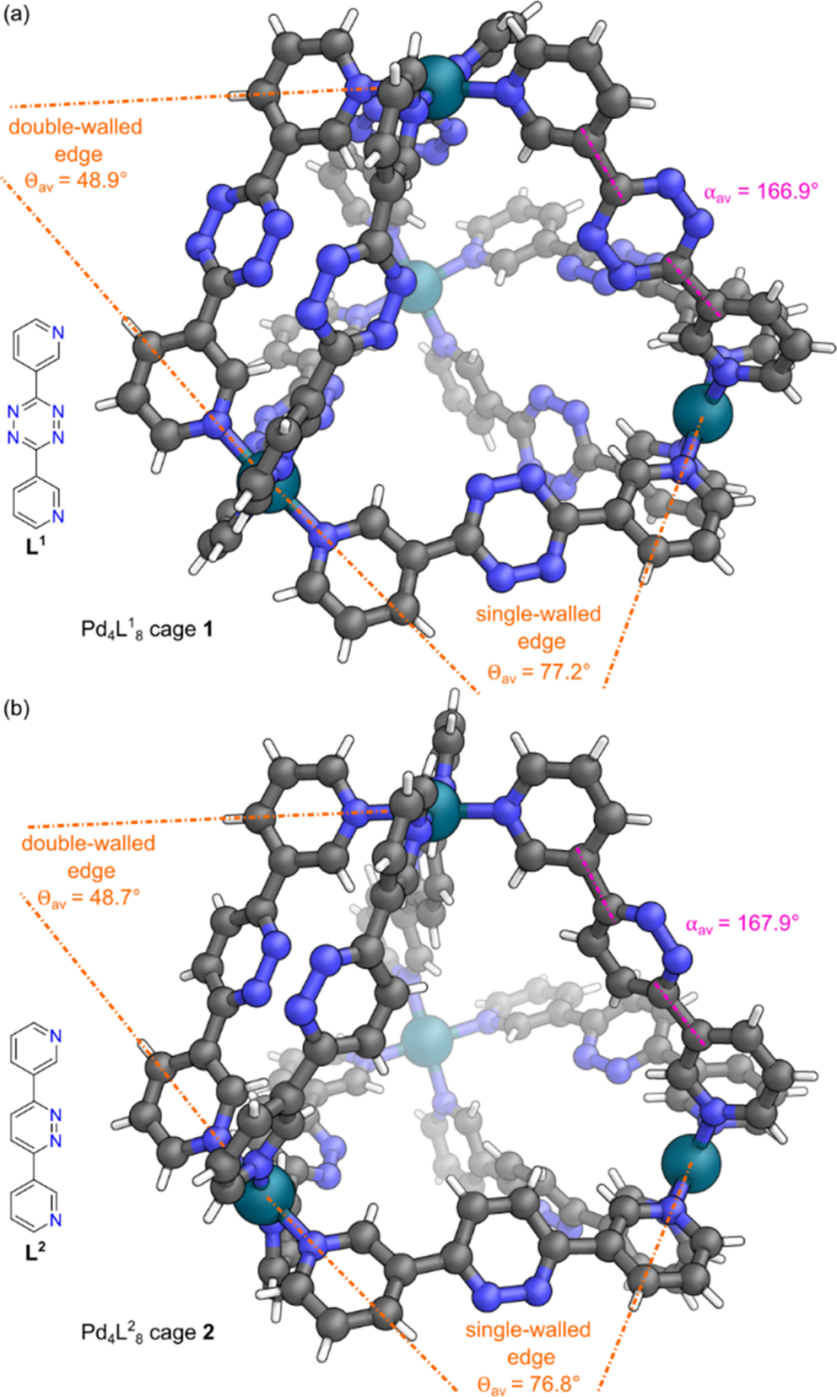
Representations of the SCXRD structures of (a) cage **1** and (b) cage **2**. Disorder, solvent and counteranions have been omitted for clarity. Color: C = gray, N = blue, Pd = turquoise, H = white.

Electrospray ionization-mass spectrometry (ESI-MS) analysis revealed peaks corresponding to a Pd_4_L^1^_8_ assembly, with well-resolved isotopic distribution patterns (Figure S22). Crystals suitable for single crystal X-ray diffraction (SCXRD) analysis were obtained by vapor diffusion of Et_2_O into a CD_3_CN solution of cage **1** revealing in the solid-state cage **1** possessed idealized *D*_2*d*_ point group symmetry (Figure 3a) (Section S10.3 in the Supporting Information), with an average Pd–Pd distance of 11.2 Å along a double-walled edge and 12.2 Å along a single-walled edge. The ideal angle between coordination planes in a tetrahedron is 70.5°. In cage **1**, the metal–ligand coordination vectors varied between single-walled edges and double-walled edges with Θ_av_ = 77.2 ± 12.5° in a single-walled edge and Θ_av_ = 48.9 ± 2.7° in a double-walled edge (Section S8.1 in the Supporting Information).

Before attempting to introduce structural changes via IEDDA, we examined whether Pd_4_L^1^_8_ cage **1** could undergo successful PAM with a simpler reaction partner. Conceptually IEDDA with acetylene is ideal, as this would just change −N=N– to −CH=CH–, however acetylene is sluggish to react and difficult to handle. Norbornadiene (NBD) behaves as functionally equivalent to acetylene, as after the IEDDA and retro-DA to lose nitrogen, the intermediate undergoes a second retro-DA to lose cyclopentadiene, directly forming the unsubstituted pyridazine.

Addition of NBD (3 equiv per tetrazine) to cage **1** resulted in a color change from pink to pale yellow over 20 min at 40 °C, indicative of tetrazine to pyridazine conversion on the ligand backbone. Monitoring the reaction through in situ ^1^H NMR spectroscopy showed broadening of signals at intermediate reaction times (∼5 min) (Figure 2b(iii)), likely due to desymmetrization from partial reaction, but no signals from free tetrazine ligand **L**^**1**^ were observed. By 20 min, a new set of sharp signals had appeared from product pyridazine cage **2** and all broad peaks had disappeared; the reaction was monitored up to 30 min with no further changes (Section S6.1 in the Supporting Information). New signals (e and e′) from the pyridazine C–H were apparent with a similar two signals per ligand environment (Figure 2b(iv)), suggesting pyridazine cage **2** was also a Pd_4_L^2^_8_ tetrahedron. This was further confirmed with ESI-MS analysis (Figure S27). Crystals suitable for SCXRD analysis were grown via vapor diffusion of *i*Pr_2_O into a CD_3_CN solution of cage **2**, with the solid-state structure of cage **2** also possessing idealized *D*_2*d*_ point group symmetry (Figure 3b) (Section S10.4 in the Supporting Information). Cage **2** possessed similar average Pd–Pd distances of 11.1 Å along a double-walled edge and 12.2 Å along a single-walled edge. Again, the metal–ligand coordination vectors varied between single-walled edges and double-walled edges, with Θ_av_ = 76.8 ± 12.3° in a single-walled edge and Θ_av_ = 48.7 ± 2.5° in a double-walled edge (Section S8.2 in the Supporting Information).

The convergence of supramolecular assemblies typically relies on rigid ligand backbones to limit the range of Θ_av_. This work shows that controlled addition of a certain amount of flexibility assists in regulating the conversion of one structure to another. This is achieved through IEDDA with substituted alkenes, with the nonplanar conformation of the 1,4-dihydropyridazine central ring in the product imparting greater flexibility to Θ_av_. When any ligand is made more flexible, a wider range of structures can form,^31^ but entropic factors favor smaller assemblies, and this was observed thermodynamically in this system.

**Figure 4:**
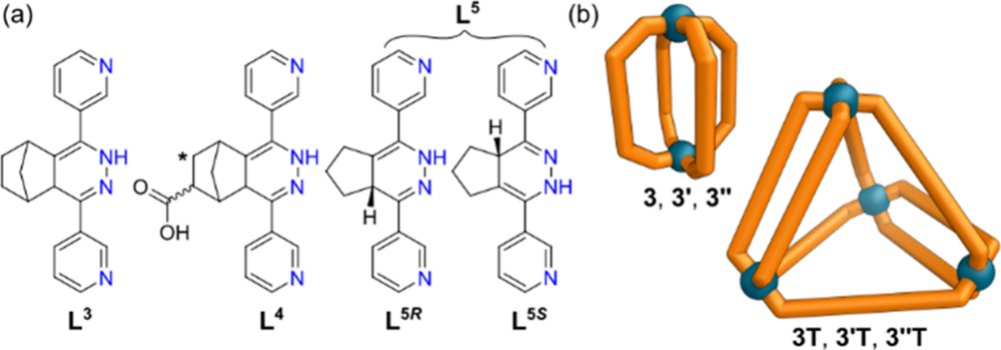
(a) Structures of 1,4-dihydropyridazine ligands **L**^**3**^, **L**^**4**^, and **L**^**5**^. Ligand **L**^**3**^ exists as multiple stereoisomers; ligand **L**^**4**^ exists as both multiple stereoisomers and as regioisomers with the COOH substituent also possible at the position marked with an *; ligand **L**^**5**^ exists as two enantiomers only: **L**^**5*R***^ and **L**^**5*S***^. (b) Cartoon representations of lantern cages **3**, **3′**, and **3**″, and double-walled tetrahedral cages **3T**, **3′T**, and **3**″**T**.

We chose to first examine the IEDDA chemistry with norbornene (NB). Upon reaction of NB (3 equiv per tetrazine) with cage **1**, the ^1^H NMR signals broadened over the course of 18 h at 35 °C and the spectrum remained highly complex even after extended reaction times. Despite the complexity of the ^1^H NMR spectrum, there was a group of signals in the DOSY NMR spectrum (Figure S37), with log *D* = −9.13, indicating that discrete metallosupramolecular assemblies existed, but were of smaller size than cage **1**. ESI-MS analysis of this mixture clearly indicated the peaks expected from a Pd_2_L^3^_4_ assembly **3** (Figures 4 and S39), which typically are lantern structures, alongside smaller peaks from a Pd_4_L^3^_8_ assembly **3T** (Figures 4 and S40).

Our hypothesis was that PAM was quantitative, giving initially Pd_4_L^3^_8_ tetrahedron **3T** as the kinetic product. While the more flexible ligand **L**^**3**^ could fit into the tetrahedron structure, its greater flexibility appeared to allow transformation to the lantern assembly. The complex nature of the NMR spectrum was attributed to both this mixture of structures and mixtures of isomers of each structure. It is important to highlight that 1,4-dihydropyridazine ligand **L**^**3**^ exists as four stereoisomers (there is a carbon stereocenter (*R*/*S*) on the central ring and the hydrogen on that center can be both *syn*/*anti* to the one carbon bridge). **L**^**3**^ is also unsymmetrical head to tail, thus a very large number of isomeric cage architectures are possible.

Crystals of lantern **3**, suitable for SCXRD were successfully obtained by vapor diffusion of *i*Pr_2_O into an CH_3_CN solution of this mixture (Figure S63), confirming the Pd_2_L^3^_4_ lantern geometry of **3** in the solid state, with a Pd–Pd distance of 8.6 Å. The 1,4-dihydropyridazine ligand enabled a significant twist and bend in the ligand backbone, allowing an average angle, Θ_av_ = 5.2°, between metal–ligand coordination vectors to be achieved, much closer to the ideal angle of 0° for a lantern than possible in the starting ligand **L**^**1**^. However, due to the multiple possible stereoisomers of **L**^**3**^, its flexible nature, and the crystallographic processing undertaken, while the overall geometry of lantern **3** is clear, the angle between the C–C bonds, α_av_, cannot be accurately determined in this structure (Section S8.3 in the Supporting Information).

Due to the sparse literature on the rate of air oxidation of dihydropyridazines, the reactions were then repeated under more rigorously anoxic conditions using Schlenk techniques and in situ monitoring in J-Young NMR tubes. The resultant spectra were similarly complex, indicating that partial oxidation was not the reason for this complexity. Other structures of Pd*_n_*L_2*n*_ empirical formulas in the literature have also given broad signals with mixed or reduced symmetry ligands, so the complexity of our spectra was not unexpected given the energetic differences between some of the structures could be very small.^32^ The ESI-MS of the anoxic sample immediately post reaction indicated a higher proportion of tetrahedron **3T** (Figure S57), however after 13 days, the ESI-MS of the anoxic sample showed an almost complete loss of tetrahedral cage signals and conversion to lantern cage **3**, supporting our hypothesis of a kinetic product Pd_4_L^3^_8_ assembly **3T**, with the thermodynamic product being Pd_2_L^3^_4_ assembly **3**.

It was suspected the size of the alkene substituent could have a substantial influence on the time taken for this tetrahedron to lantern transformation to occur. IEDDA with a functionalized norbornene (5-norbornene-2-carboxylic acid) also led to the formation of Pd_2_L^4^_4_ lantern architecture **3′** as observed via ESI-MS analysis (Figure S44), again with a highly complex ^1^H NMR spectrum (Figure S41). Interestingly when the reaction was performed with Schlenk techniques, only a small quantity of Pd_4_L^4^_8_ tetrahedron **3′T** was produced along with the Pd_2_L^4^_4_ lantern **3′** (Figure S58), and there was little further change after 13 days. We attribute this to the greater steric bulk of the substituted norbornyl moiety on the central ring of **L**^**4**^ causing a greater degree of steric hindrance in the double-walled edges of tetrahedron **3′T**, similar to phenomena previously observed in other systems.^33^ Hence, kinetic product Pd_4_L^4^_8_ tetrahedron **3′T** has a smaller barrier for conversion to Pd_2_L^4^_4_ lantern **3′**, or the equilibrium composition favors more of lantern **3′**, or a combination of both of these effects (Section S7.2 in the Supporting Information). We were unable to grow crystals suitable for SCXRD of assemblies from ligand **L**^**4**^.

**Figure 5:**
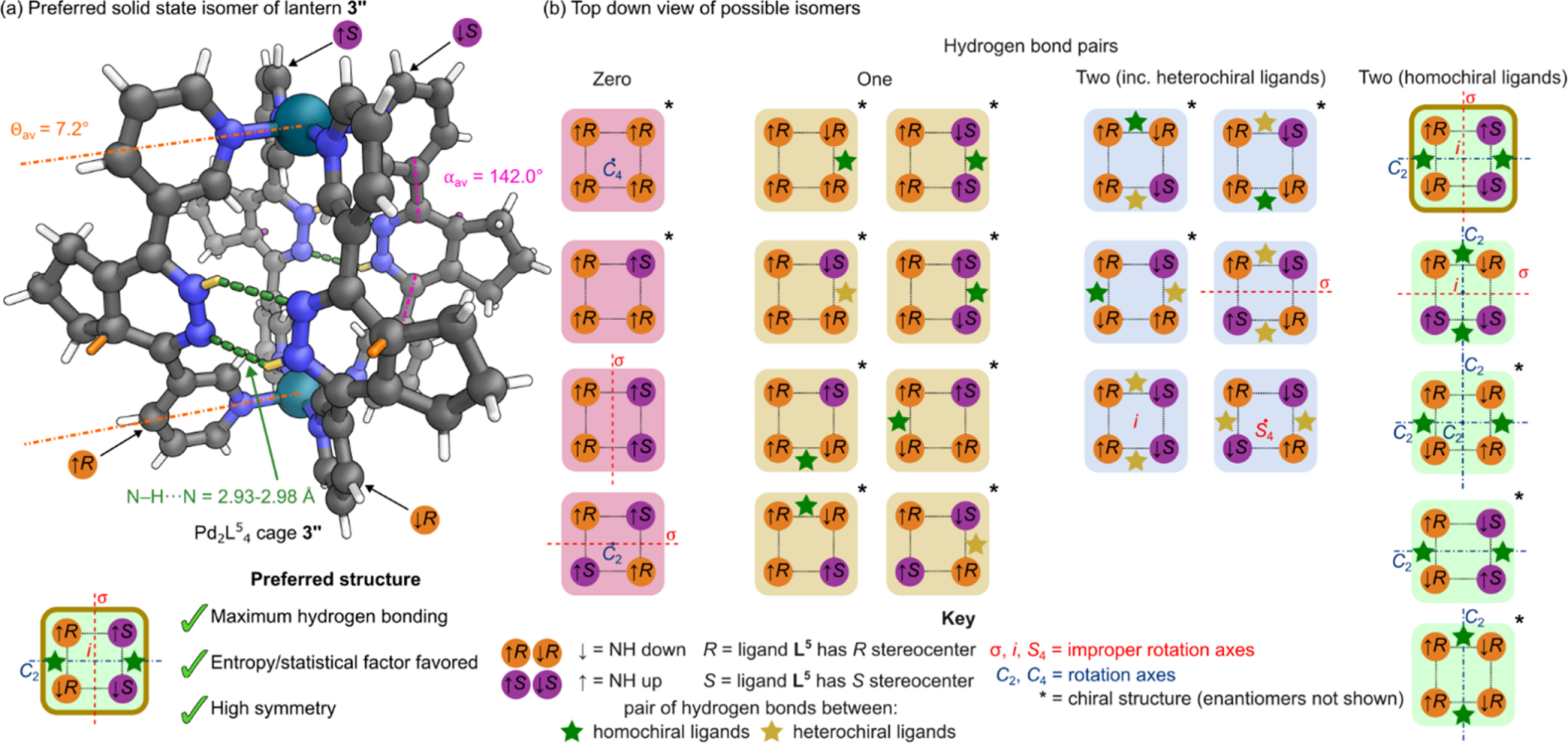
(a) A representation of the SCXRD structure of the single isomer of lantern cage **3**″ found in the solid state. Disorder, solvent, and counteranions have been omitted for clarity. Color: C = gray, *N* = blue, Pd = turquoise, H = orange on *R* stereocenter, purple on *S* stereocenter, yellow hydrogen bonded, white other. (b) Cartoon representation of the 39 possible isomers of cage **3**″ viewed from the top. These consist of 16 enantiomeric pairs of chiral structures (only one enantiomer shown) and seven achiral structures, with the symmetry properties and hydrogen bonding possibilities labeled.

To investigate the effect of a reduction in the steric bulk of the alkene partner, we next investigated IEDDA with cyclopentene (CP). The corresponding 1,4-dihydropyridazine ligand **L**^**5**^ now only exists as a pair of enantiomers due to the *R*/*S* carbon stereocenter on the central ring (denoted **L**^**5*****R***^ and **L**^**5*****S***^ respectively) (Figure 4a). The reaction of CP (3 equiv per tetrazine) with Pd_4_L^1^_8_ cage **1** appeared to reach completion after 30 min at 35 °C. The ^1^H NMR spectrum of the resultant mixture was broad, both when the reaction was performed using Schlenk techniques and without; reducing the temperature of the ^1^H NMR sample to 245 K did not lead to sharpening nor simplification of the spectra (Figure S47). The DOSY NMR spectrum had an average diffusion coefficient, log *D* = −9.16, although the overall range of values was broader than starting tetrahedral cage **1** (Figure S48). The reaction using Schlenk techniques also contained substantial signal intensity for the tetrahedral topology **3**″**T** in addition to the lantern **3**″ in the ESI-MS analysis (Figure S59). We successfully obtained single crystals of lantern **3**″ suitable for SCXRD by vapor diffusion of *i*Pr_2_O into a CD_3_CN solution of this mixture (Figure 5a). Diffraction from the best of these crystals confirmed the Pd_2_L^5^_4_ lantern architecture of **3**″ in the solid state, with a similar Pd–Pd distance of 8.8 Å to that of lantern **3** at 8.6 Å. Again, the flexibility of the 1,4-dihydropyridazine enabled significant ligand twisting and bending, with the angle between the C–C bonds to adjoining substituents reduced to an α_av_ = 142.0 ± 2.5°, leading to an average angle, Θ_av_ = 7.2 ± 0.4°, between metal–ligand coordination vectors (Section S8.4 in the Supporting Information).

Further analysis of the structure revealed the ligands were hydrogen bonded in pairs between the N–H and N groups on the 1,4-dihydropyridazine rings. Each pair possessed two hydrogen bonds with donor–acceptor distances of 2.977(9) and 2.932(9) Å, in a cyclic array of type *R*_2_^2^(6).^34^ Interestingly this hydrogen bonding appeared to be directing the integrative sorting and orientation of the **L**^**5*****R***^ and **L**^**5*****S***^ ligands within this solid-state structure. One side of the lantern possessed a pair of **L**^**5*****R***^ ligands hydrogen bonded in a head-to-tail arrangement, while the other possessed a pair of **L**^**5*****S***^ ligands hydrogen bonded in a head-to-tail arrangement. The idealized overall structure possessed a central mirror plane, *C*_2_ axis, and had inversion symmetry, with *C*_2*h*_ point group symmetry.

Thus, this lantern possessed a combination of structural features incredibly rare among Pd_2_L_4_ architectures. The vast majority of Pd_2_L_4_ lanterns are homoleptic (four identical L) with the ligands also being symmetric (no head (H) to tail (T) isomerism possible).^35^ Mixed ligand architectures require an integrative (or social) sorting of the ligands^36^ and have almost exclusively been studied with symmetric ligands. Reported examples of this heteroleptic *cis*-Pd_2_L^A^_2_L^B^_2_ assembly have used approaches such as coordination sphere engineering (addition of bulky substituents around the pyridine donors),^37^ shape complementarity (tilting the two metal coordination planes),^38^ guest encapsulation,^39^ and ligand-ligand hydrogen bonding^40^ to favor this structure.

Conversely, studies with unsymmetrical ligands capable of head (H) to tail (T) isomerism have focused on homoleptic assemblies. The *cis*-HHTT arrangement in homoleptic cases has been selected for through hydrogen bonding interactions,^41^ guest binding,^42^ or altering the ligand aspect ratio.^43^ Where the head and tail end of the ligand differ significantly, computational approaches have shown the *cis*-HHTT arrangement to be lowest in energy.^18e,44^ We are aware of just a single report of a lantern that possesses both these structural features. Bloch and Fallon reported a Pd_2_L_4_ lantern with shape-shifting bullvalene ligands, with the same isomeric possibilities as lantern cage **3**″.^32a^

There are 16 enantiomeric pairs of chiral structures (32) and seven achiral structures, making 39 possible isomers of lantern cage **3**″ in total (Figure 5b, Section S9 in the Supporting Information). Of the 39 isomers, in six the ligand arrangement precludes hydrogen bonding from occurring (pink background); a further 16 can only have hydrogen bonding between one pair of edges (yellow background). All these structures are significantly less stabilized than the one observed. Of the remaining isomers, another nine possess two pairs of hydrogen bonded edges (blue background), but these include at least one hydrogen bond pair between heterochiral ligands (**L**^**5*****R***^ matched with **L**^**5*****S***^). These too would be energetically different to the hydrogen bonding between homochiral ligands observed in the crystal structure of lantern **3**″. Of the final eight structures which can have both pairs of hydrogen bonds between homochiral edges (green background), the achiral structure of **3**″ (highlighted in gold) possesses both high symmetry and a high relative entropy (making the structure favored on statistical grounds) (Section S9.3 in the Supporting Information). Interestingly, the single achiral structure observed by Bloch and Fallon was the same isomer as cage **3**″, i.e., *cis*-L^a^(H)L^a^(T)L^b^(T)L^b^(H), which they rationalized on only the latter two of these three factors: higher relative entropy and higher symmetry. The mechanism and rate of interconversion are also vastly different, with the bullvalene system interconverting very rapidly through pericyclic Cope rearrangements as opposed to the much slower ionic tautomerization processes required here.

Due to a high amount of unfixable disorder in the SCXRD data of NB lantern **3**, we are unable to say with certainty that the same single isomer forms in the solid state of this structure. The distance and relative orientation of the dihydropyridazine rings indicates the presence of hydrogen bonding and head-to-tail ligand pairing. However, individual atom hybridization cannot be determined nor the stereochemistry at the carbon centers, meaning not all other possibilities can be excluded (Section S9.6 in the Supporting Information).

While looking for crystals of other lantern isomers of **3**″ (which we never found), we obtained a crystal of a Pd_4_L_8_ structure (Section S10.7 in the Supporting Information). We have denoted this structure **3**″**T**. The tetrahedron appears composed of a mixture of both dihydropyridazine ligands **L**^**5**^ and oxidized pyridazine ligands **L**^**6**^ which are disordered substitutionally and orientationally. The electron density maps in the region of the disordered linker moieties of **3**″**T** are diffuse, preventing the clear identification of hydrogen positions and carbon hybridizations, however, the gross conformation of the ligands along the double-walled edges clearly indicates the presence of hydrogen bonds between them, confirming that at least one of each pair is in the dihydro form **L**^**5**^. In the ESI-MS data of samples of tetrahedral cage **1** which had undergone PAM with CP, we saw the full range of no ligand oxidation (all **L**^**5**^) to full ligand oxidation (all **L**^**6**^) depending on the conditions the sample had experienced before analysis (Section S7.1 in the Supporting Information).

**Figure 6:**
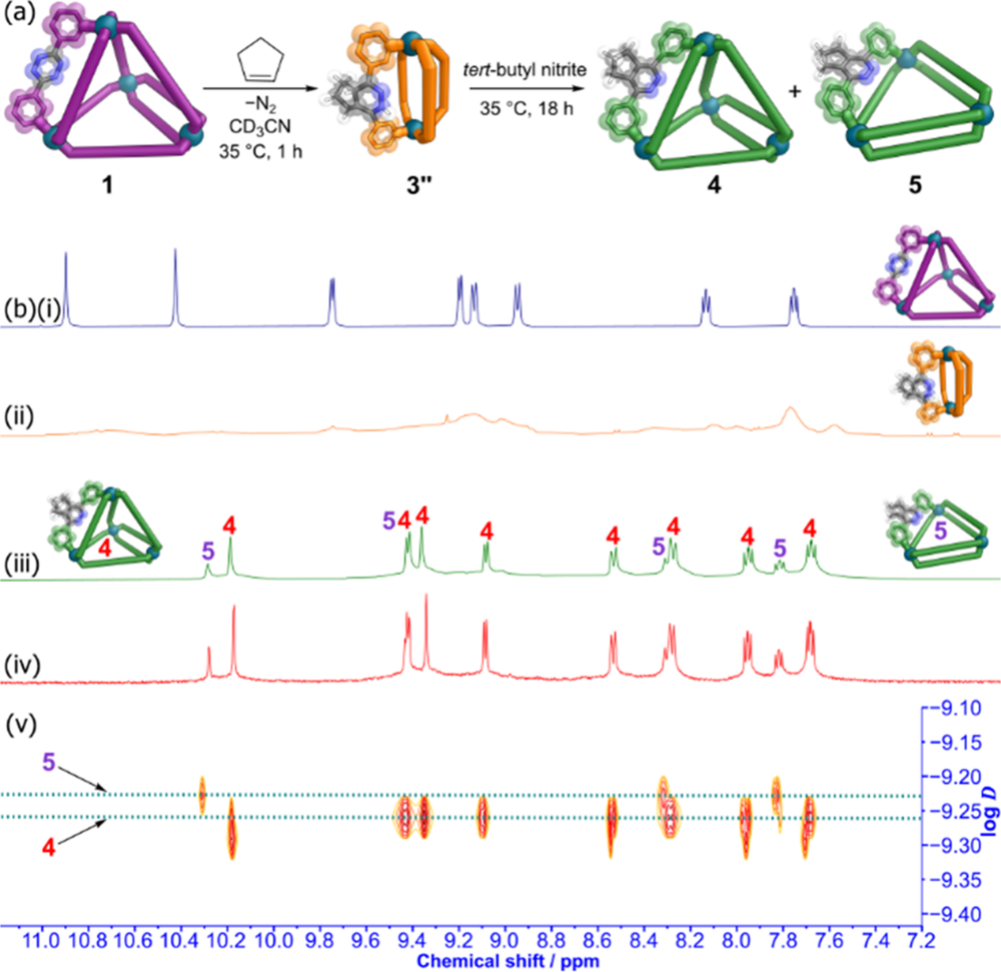
(a) Post-assembly modification of tetrahedral cage **1** to lantern cage **3**″ and secondary oxidation to tetrahedral cage **4** and triangular cage **5**. (b) Stacked partial ^1^H NMR spectra (CD_3_CN, 500 MHz, 298 K) of: (i) cage **1** (blue); (ii) cage **3**″ 15 min after addition of CP at 308 K (orange); (iii) formation of cages **4** and **5** 18 h after addition of *tert*-butyl nitrite at 308 K (green); (iv) formation of cages **4** and **5** after eight days heating at 308 K (red); (v) DOSY NMR spectra of cages **4** and **5**.

We postulated the reoxidation of the flexible 1,4-dihydropyridazines to rigid pyridazines would transform the lantern structures back to double-walled tetrahedra again. While our studies had supported the literature consensus of a ‘slow’ reoxidation of 1,4-dihydropyridazines under ambient conditions, we wondered whether we could perform this oxidation more quickly upon addition of an external oxidizing agent so that we could trigger a transformation of a lantern to tetrahedron. Addition of *tert*-butyl nitrite (1.2 equiv per dihydropyridazine) to a CD_3_CN solution of the cage **3**″/**3**″**T** mixture led after 18 h at 35 °C to the complete sharpening of the broad signals from **3**″/**3**″**T** to a new ^1^H NMR spectrum which possessed three sets of signals per ligand environment (Figure 6b (iii)). Two sets of these signals were present at equal intensity, consistent with the formation of **4**, the fully reoxidized Pd_4_L^6^_8_ tetrahedron. The third set of signals had a lower intensity. We postulated that these were from **5**, a Pd_3_L^6^_6_ double-walled triangle architecture, as these are often formed in equilibrium with double-walled tetrahedra,^30,45^ and have only one set of ligand signals. This was supported by ESI-MS analysis of this mixture, where peaks for both Pd_3_L^6^_6_ and Pd_4_L^6^_8_ assemblies were detected (Figures S31 and S32). The DOSY NMR spectrum showed signals with log *D* = – 9.23 for the postulated Pd_3_L^6^_6_ triangle **5** and log *D* = – 9.27 for Pd_4_L^6^_8_ tetrahedron **4**.

The oxidation could also be achieved via heating the sample at 35 °C over the course of eight days while standing in an NMR tube. We performed a separate synthesis of oxidized ligand **L**^**6**^ and studied its direct self-assembly (Section S5.3 in the Supporting Information). This gave a ratio of cage concentrations of approximately 64% of tetrahedron **4** to 36% of triangular cage **5**, or a ratio of 71% of ligand **L**^**6**^ being in tetrahedron **4** to 29% in triangle **5** (Section S5.3 in the Supporting Information), the same ratio as obtained through both PAM oxidation conditions, confirming an equilibrium composition was reached through PAM.

**Figure 7:**
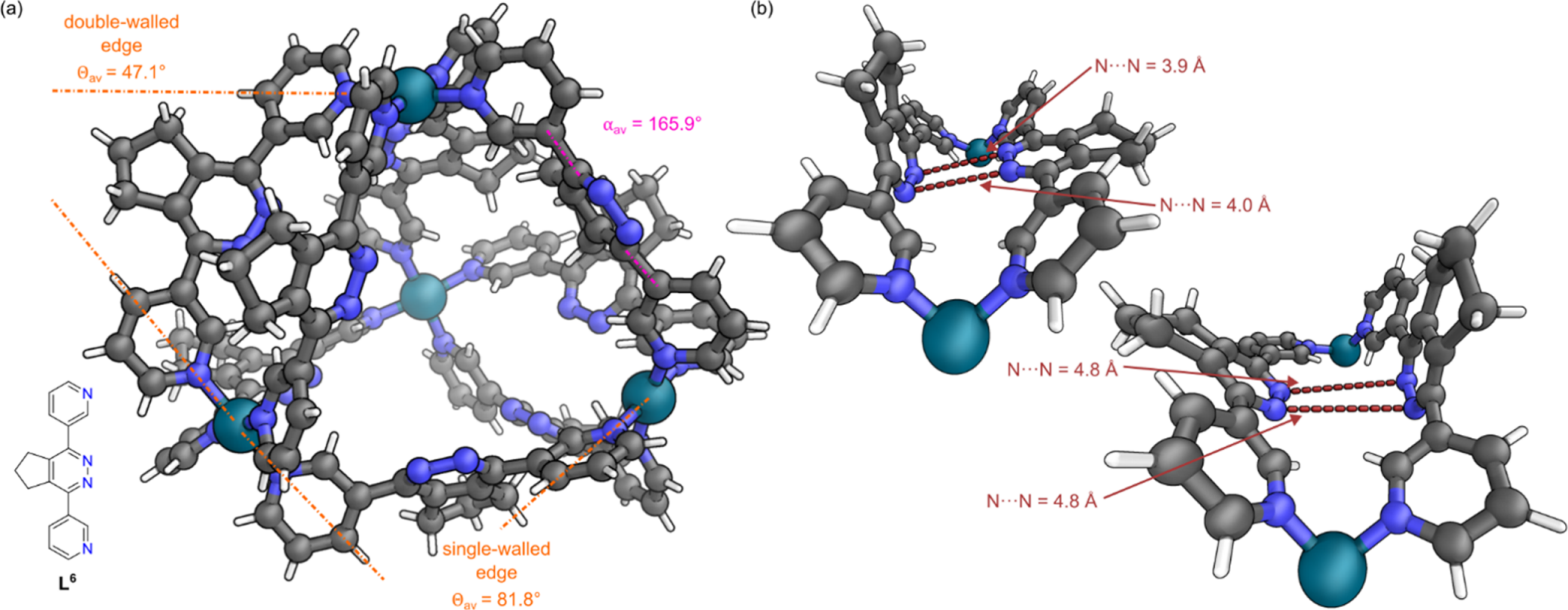
(a) Representation of the SCXRD structure of cage **4**. Disorder, solvent, and counteranions have been omitted for clarity. Color: C = gray, N = blue, Pd = turquoise, H = white. (b) Views along the two double-walled edges of cage **4**. The large N-to-N distances and the relative orientation of the central rings confirm no hydrogen bonding interactions are present and the rings are in their fully oxidized state.

Crystals suitable for SCXRD were obtained by vapor diffusion of *i*Pr_2_O into a CD_3_CN solution of the mixture of cages **4** and **5**. The crystal structure of tetrahedral cage **4** obtained indicated that all eight **L**^**6**^ ligands were in their oxidized form, with the structure possessing idealized *D*_2*d*_ point group symmetry (Figure 7a) (Section S10.8 in the Supporting Information). Cage **4** had similar average Pd–Pd distances of 10.9 Å along a double-walled edge and 12.1 Å along a single-walled edge to cages **1** and **2**, and a similar average angle, Θ_av_, between metal–ligand coordination vectors, of 64.5 ± 24.1°. In contrast to the crystal structures of the mixed ligand tetrahedron **3**″**T** and lanterns **3** and **3**″, the pyridazine rings in the double-walled edges of **4** were not oriented toward each other, with N-to-N distances larger than what would be expected if N–H to N hydrogen bonds were present (Figure 7b). We were unable to obtain X-ray quality single crystals of triangular cage **5** from this equilibrium mixture. Without a single crystal structure of triangle **5**, it is difficult to conclude why addition of the cyclopentyl substituent makes this structure of similar energy to tetrahedron **4** (when no triangle is observed with unsubstituted pyridazine ligand **L**^**2**^). With similar ideal angles between coordination vectors (70.5° vs 60°), slight alterations in ligand bend and twist angles or possibility of C–H-π interactions may alter the relative energy of the tetrahedral and triangular structures.^46^

Interestingly, addition of *tert*-butyl nitrite (3 equiv per dihydropyridazine) to CD_3_CN solutions of both cages **3**/**3T** and **3′**/**3′T** led to the reaction mixture being converted into insoluble precipitate. Attempts at direct assembly of the oxidized norbornene pyridazine ligand with [Pd(CH_3_CN)_4_](BF_4_)_2_ also resulted in similar insoluble precipitate (Section S4.4 in the Supporting Information). We attributed this to the bulk of the norbornyl substituent on the central ring hindering formation of both the tetrahedron and the triangle when in the fully oxidized and planar form. This is because both these structures possess double-walled edges, and so their formation would be more substantially hindered by bulkier substituents, thus further indicating the delicate balance of kinetics and thermodynamics on display during this work.

## Conclusions

In summary, we have demonstrated the transformation from a Pd_4_L_8_ tetrahedral cage to Pd_2_L_4_ lanterns by introducing a controlled amount of flexibility in the ligand backbone through the IEDDA PAM reaction, and then further converting them to Pd_4_L_8_ tetrahedra and Pd_3_L_6_ triangles by rigidifying the ligand. This is underpinned by the preferred 1,4-tautomer of the dihydropyridazine product, formed by tetrazine-alkene IEDDA reaction, occupying a nonplanar conformation, reducing the angle between ligand vectors from an average of 63.1° to 7.2°. The PAM is quantitative and initially gives a kinetic product Pd_4_L_8_ tetrahedron, which transforms to a thermodynamic product Pd_2_L_4_ lantern over a period of time governed by the size of the added substituent. In the case of lantern **3**″, a single isomer (of 39 possible) is favored in the solid state, with an extremely rare combination of both head-to-tail orientation selection and enantiomer selection. Control over this selection is achieved through hydrogen bonding, entropy, and symmetry. We envisage this transient head-to-tail hydrogen bonded motif may be highly useful in other systems. Upon oxidation however, ligand planarity returns, and this induces a further rearrangement to return to a modified scaffold of the original Pd_4_L_8_ tetrahedron **4**, accompanied with a Pd_3_L_6_ triangle **5**.

Supramolecular structural transformations have been used to develop responsive soft materials, systems capable of cargo capture and release, and photoswitchable catalysts, controlled by various and sometimes multiple stimuli^47^ including guest binding,^48^ ligand and metal substitution,^49^ and photoisomerization reactions.^50^ However, to realize these applications, fine control over the shape and size of resultant assemblies is required. Our present approach allows us to make simple ligand alterations that have profound structural consequences. Many promising applications of metal–organic cages such as catalysis, separations, and cargo transport rely on a precisely tailored cage and cavity to optimize binding, making strategies to finely tune cage characteristics ever more crucial.

## Supporting Information Available

Experimental procedures, NMR spectra (^1^H, COSY, NOESY, DOSY, ^13^C), high-resolution ESI-MS, and analysis of the SCXRD data (PDF)
