# Supplementary material for: Structural Transformations of Metal–Organic Cages through Tetrazine-Alkene Reactivity
Source: J Am Chem Soc. 2024 Sep 5;146(41):28233–41. doi: 10.1021/jacs.4c08591 (PMC11487605; doi:10.1021/jacs.4c08591)
Supplement: Supplementary file 1 — ja4c08591_si_001.pdf [file ja4c08591_si_001.pdf]

Supporting Information for

Structural Transformations of Metal–Organic Cages through Tetrazine-Alkene Reactivity

Martin R. Black,[a] Soumalya Bhattacharyya,[a] Stephen P. Argent,[a] and Ben S. Pilgrim[a*]

[a]*School of Chemistry, University of Nottingham, University Park, Nottingham, NG7 2RD, UK*

*E-mail: ben.pilgrim@nottingham.ac.uk

Contents

S1 Reagents and General Materials ... 5

S2 Characterisation and Analysis Methods ... 6

S2.1 NMR Spectroscopy ... 6

S2.2 Mass Spectrometry ... 6

S2.3 Access to Raw Data ... 6

S3 Ligand and Cage Reference Charts ... 7

S3.1 Ligand Reference Chart ... 7

S3.2 Cage Reference Chart ... 8

S4 Ligand Synthesis and Characterisation ... 9

S4.1 Synthesis of 3,6-di(pyridin-3-yl)-1,2,4,5-tetrazine L^1 ... 9

S4.2 Synthesis of 3,6-di(pyridin-3-yl)pyridazine L^2 ... 12

S4.3 Synthesis of 1,4-di(pyridin-3-yl)-6,7-dihydro-5H-cyclopenta[d]pyridazine L^6 ... 15

S4.4 Synthesis of 1,4-di(pyridin-3-yl)-5,6,7,8-tetrahydro-5,8-methanophthalazine L^7 ... 17

S5 Synthesis and Characterisation of Metal-Organic Cages ... 21

S5.1 Self-assembly of tetrahedral cage $[1](BF_4)_8$ / $[Pd_4L^1_8](BF_4)_8$ ... 21

S5.2 Self-assembly of tetrahedral cage $[2](BF_4)_8$ / $[Pd_4L^2_8](BF_4)_8$ ... 25

S5.3 Self-assembly of tetrahedral cage $[4](BF_4)_8$ / $[Pd_4L^6_8](BF_4)_8$ and triangular cage $[5](BF_4)_6$ / $[Pd_3L^6_6](BF_4)_6$ ... 29

S6 Post-Assembly Modification of Cages ... 34

S6.1 Post-assembly modification route to tetrahedral cage $[2](BF_4)_8$ / $[Pd_4L^2_8](BF_4)_8$ ... 34

S6.2 Post-assembly modification route to lantern cage $[3](BF_4)_4$ / $[Pd_2L^3_4](BF_4)_4$ and tetrahedral cage $[3T](BF_4)_8$ / $[Pd_4L^3_8](BF_4)_8$ ... 37

S6.3 Post-assembly modification route to lantern cage $[3'](BF_4)_4$ / $[Pd_2L^4_4](BF_4)_4$ and tetrahedral cage $[3'T](BF_4)_8$ / $[Pd_4L^4_8](BF_4)_8$ ... 41

S6.4 Post-assembly modification route to lantern cage $[3''](BF_4)_4$ / $[Pd_2L^5_4](BF_4)_4$ and tetrahedral cage $[3''T](BF_4)_8$ / $[Pd_4L^5_8](BF_4)_8$ ... 45

S6.5 Post-assembly modification route to tetrahedral cage $[4](BF_4)_8$ / $[Pd_4L^6_8](BF_4)_8$ and double-walled triangle cage $[5](BF_4)_6$ / $[Pd_3L^6_6](BF_4)_6$ ... 51

S7 ESI-MS Studies of Cage Systems ... 53

S7.1 ESI-MS studies of the oxidation of dihydropyridazine ligands ... 53

S7.2 ESI-MS studies on whether post-assembly modification led to thermodynamic mixtures of cages or kinetically trapped mixtures ... 56

S7.2.1 Lantern cage 3 and tetrahedral cage 3T ... 57

S7.2.2 Lantern cage 3' and tetrahedral cage 3'T *58*
S7.2.3 Lantern cage 3'' and tetrahedral cage 3''T *59*
S7.2.4 Lantern cage 3'' and tetrahedral cage 3''T at higher temperatures *60*

S8 Determination of bend angles and ligand vector angles 62

S8.1 Cage 1 64
S8.2 Cage 2 68
S8.3 Cage 3 72
S8.4 Cage 3'' 74
S8.5 Cage 4 76
S8.6 Ligand L^1 80
S8.7 Ligand L^2 80
S8.8 Ligand L^6 80

S9 Analysis of Isomers of Lantern Cage 3'' 81

S9.1 Discussion of isomer possibilities 81
S9.2 Table showing equivalence of different structures 85
S9.3 Similarity of structures 1243 and 1342 90
S9.4 Comparison to bullvalene system 91
S9.5 Consideration of hydrogen bonds 92
S9.6 Additional possibilities of isomerism in lantern 3. 93

S10 X-ray Crystallography 94

S10.1 Single Crystal Structure of L^2 97
S10.1.1 Specific crystal structure and refinement details for L^2 .... *97*
S10.2 Single Crystal Structure of L^6 98
S10.2.1 Specific crystal structure and refinement details for L^6 .... *98*
S10.3 Single Crystal Structure of tetrahedral cage 1 99
S10.3.1 Specific crystal structure and refinement details for tetrahedral cage 1 .... *99*
S10.4 Single Crystal Structure of tetrahedral cage 2 101
S10.4.1 Specific crystal structure and refinement details for tetrahedral cage 2 .... *101*
S10.5 Single Crystal Structure of 3 103
S10.5.1 Specific crystal structure and refinement details for lantern cage 3 .... *103*
S10.6 Single Crystal Structure of 3'' 105
S10.6.1 Specific crystal structure and refinement details for lantern cage 3'' *105*
S10.7 Single Crystal Structure of 3''T 107
S10.7.1 Specific crystal structure and refinement details for tetrahedral cage 3''T *107*

S10.8 Single Crystal Structure of 4 111

S10.8.1 Specific crystal structure and refinement details for tetrahedral cage 4 111

S11 References 113

S1 Reagents and General Materials

All chemicals and dry solvents were purchased from Sigma-Aldrich or Fluorochem. Commercial solvents and reagents were used without further purification unless specified. Flash column chromatography was performed using silica gel high purity grade (pore size 60 Å, 230–400 mesh particle size, Sigma-Aldrich). Automated flash column chromatography was performed using a CombiFlash NextGen 300 Automated Flash Chromatography System, with a UV-Vis detector 200-800 nm with PeakTrak software control via a 12" capacitive touchscreen, with a flow rate range of 1-300 mL min^{-1} and a maximum pressure limit of 160 psi (11 bar) TLC analyses were performed on Merck TLC silica gel 60 F_{254} plates. Product spots were visualized under UV light (λ_{max} = 254 nm) and/or by staining with potassium permanganate or vanillin solutions. Centrifugation was carried out on a Grant-bio LMC-3000 or a Corning Mini Microcentrifuge. All reactions were stirred with magnetic followers. Room temperature is taken as 293 K.

S2 Characterisation and Analysis Methods

S2.1 NMR Spectroscopy

NMR spectra were recorded at 298 K using Bruker Avance(III) 500 or 400 spectrometers. (^{1}H, 500 / 400 MHz; ^{13}C{1H}, 126 / 101 MHz; ^{19}F NMR, 471 / 376 MHz). Spectrometers were automatically tuned and matched to the correct operating frequencies. Routine ^{1}H NMR characterisation was carried out using a zg30 pulse program (30° pulse). ^{1}H and ^{13}C NMR spectra are referenced to the residual solvent peak for CD_3CN (^{1}H: 1.94 ppm, ^{13}C: 118.26 ppm) and $CDCl_3$ (^{1}H: 7.26 ppm, ^{13}C: 77.16 ppm). Deuterated acetonitrile (CD_3CN) and deuterated chloroform ($CDCl_3$) were obtained from Sigma Aldrich and used without any further purification. NMR signals are reported in terms of chemical shift (δ) in parts-per-million (ppm), multiplicity, coupling constants (in Hz), and relative integral in that order. The following abbreviations for multiplicity are used: s, singlet; d, doublet; t, triplet; qu, quartet; qn, quintet; m, multiplet; br, broad. Where spectra have been assigned this has been done on accompanying figures. Spectra were digitally processed (phase and baseline corrections, integration, peak analysis) using Mestrenova 14.0.0-23239. DOSY NMR experiments were performed on a Bruker Avance(III) 500 MHz, 5 mm BBO probe NMR spectrometer. Gradient strength was between 1.73 and 17.5 G/cm. DOSY measurements were performed using the standard pulse program, dstebpgp3s, employing a stimulated echo and longitudinal eddy-current delay (LED) using bipolar gradient pulses for diffusion using two spoil gradients. SinE.100 gradients were used. Diffusion times Δ = 200 ms and δ = 2000 µs were used for the experiments. The size of fid = 32. Individual rows of the S4 psuedo-2D diffusion databases were phased and baseline corrected. Raw DOSY data were processed using the Peak fit DOSY transform program in Mestrenova 14.0.0-23239.

S2.2 Mass Spectrometry

High resolution electrospray ionisation (ESI) mass spectra were obtained on a Bruker ESI-TOF MicroTOF II spectrometer or a Bruker Impact II.

S2.3 Access to Raw Data

Raw data are available upon reasonable request to the corresponding author Dr Ben Pilgrim ben.pilgrim@nottingham.ac.uk.

S3 Ligand and Cage Reference Charts

S3.1 Ligand Reference Chart

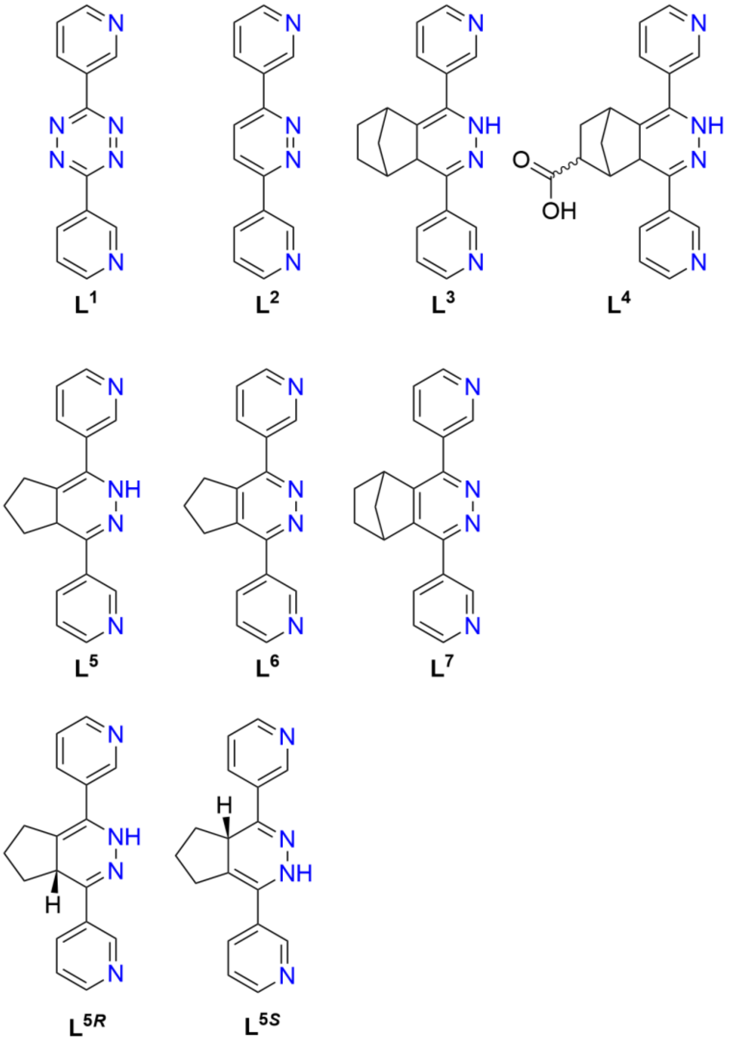

Figure S1: Ligand reference table

For ligand **L**^{4}, tautomerisation leads to two regioisomeric possibilities; only one tautomer is shown. Ligands **L**^{3}, **L**^{4}, and **L**^{5} exist as mixtures of stereoisomers. **L**^{5R} and **L**^{5S} are the two stereoisomers for ligand **L**^{5}.

S3.2 Cage Reference Chart

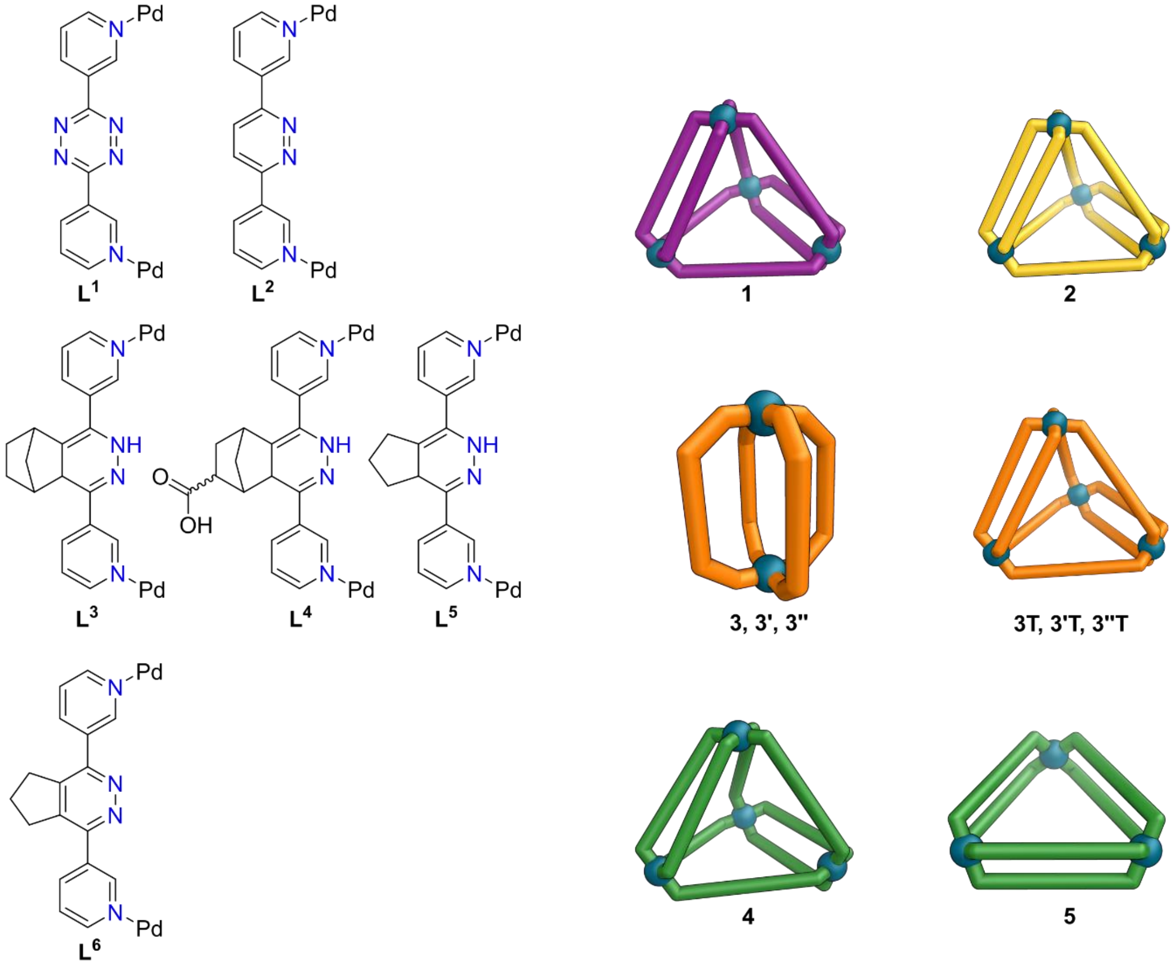

Figure S2: Cage reference chart

S4 Ligand Synthesis and Characterisation

S4.1 Synthesis of 3,6-di(pyridin-3-yl)-1,2,4,5-tetrazine L1

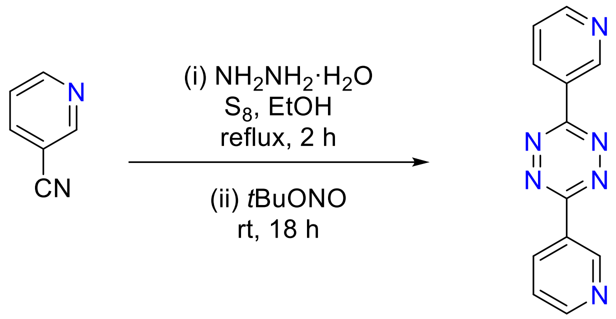

37 %

L1

Hydrazine monohydrate (912 mg, 19.2 mmol, 0.930 mL) was added slowly to a solution of 3-pyridine carbonitrile (1.00 g, 9.60 mmol) and sulfur (154 mg, 4.80 mmol) in EtOH (4.76 mL) at room temperature. The product was refluxed for 2 h and then cooled in ice bath. The precipitate was collected *via* suction filtration and washed with ice cold H_2O (64 mL) and ice cold EtOH (12 mL). The sample was then dried *in vacuo* and redissolved in $CHCl_3$/EtOH (12.4 mL / 7.70 mL), *tert*butyl nitrite (1.98 g, 19.2 mmol, 2.28 mL) was then added and the mixture stirred at room temperature for 18 h and then refluxed for 30 min. The precipitate was then collected and recrystallized from hot CH_3CN. The product was then collected *via* filtration, washed with Et_2O (10 mL), and then purified *via* flash column chromatography on silica gel (EtOAc) to give tetrazine **L1** as a purple crystalline solid (422 mg, 1.79 mmol, 37%). Data were consistent with those previously reported. [1]

^{1}H NMR (400 MHz, $CDCl_3$) δ_H: 9.88 (d, *J* = 3.1 Hz, 2H), 8.96-8.88 (m, 4H), 7.59 (dd, *J* = 8.0, 4.9 Hz, 2H).

^{13}C NMR (101 MHz, $CDCl_3$) δ_C: 163.6, 153.7, 149.7, 135.4, 127.7, 124.2.

ESI-MS (ESI, MeCN), *m/z*: calculated for $[M+H]^+$, $[C_{12}H_9N_6]^+$, 237.0883, found 237.0878.

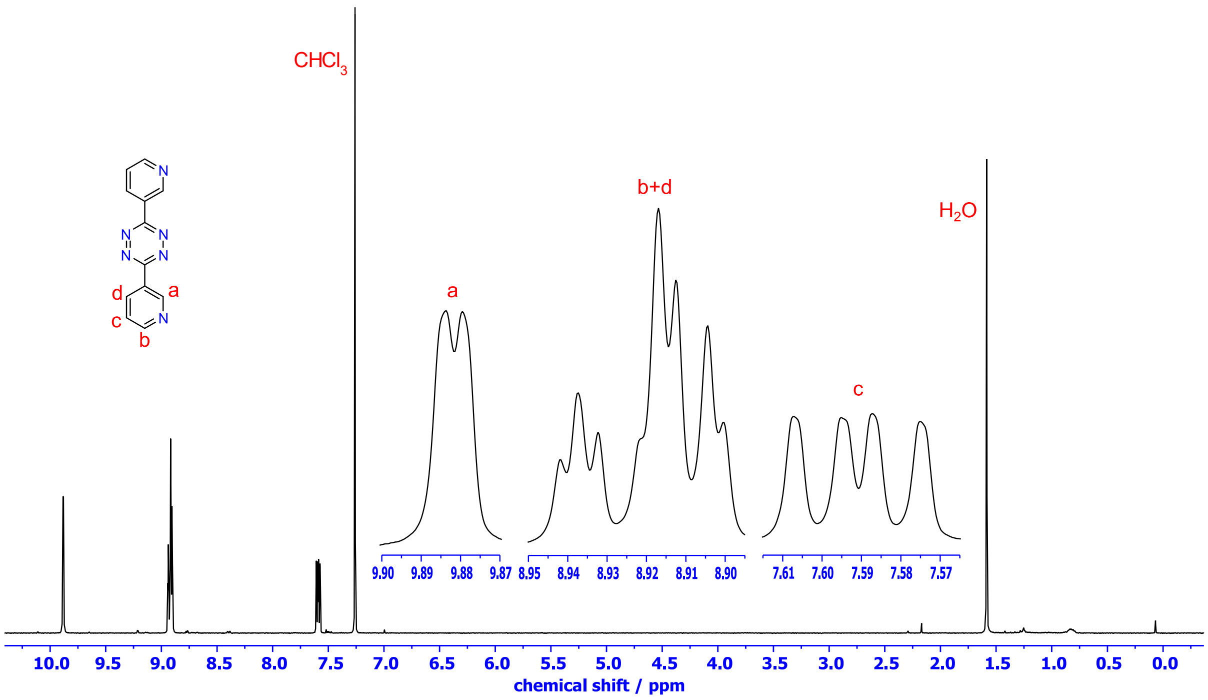

Figure S3: ^{1}H NMR spectrum of **L^{1}**
(400 MHz, 298 K, $CDCl_3$)

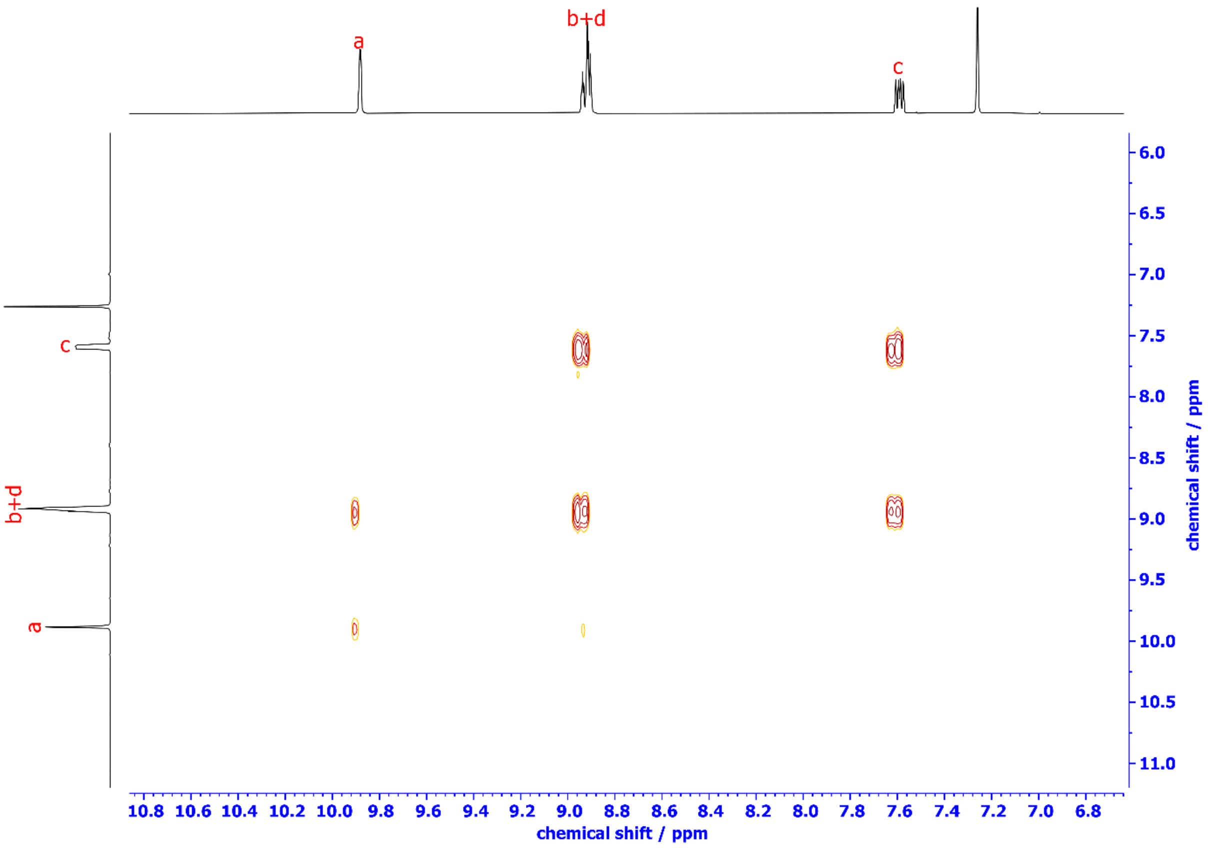

Figure S4: ^{1}H-^{1}H COSY spectrum of **L^{1}**
(400 MHz, 298 K, $CDCl_3$)

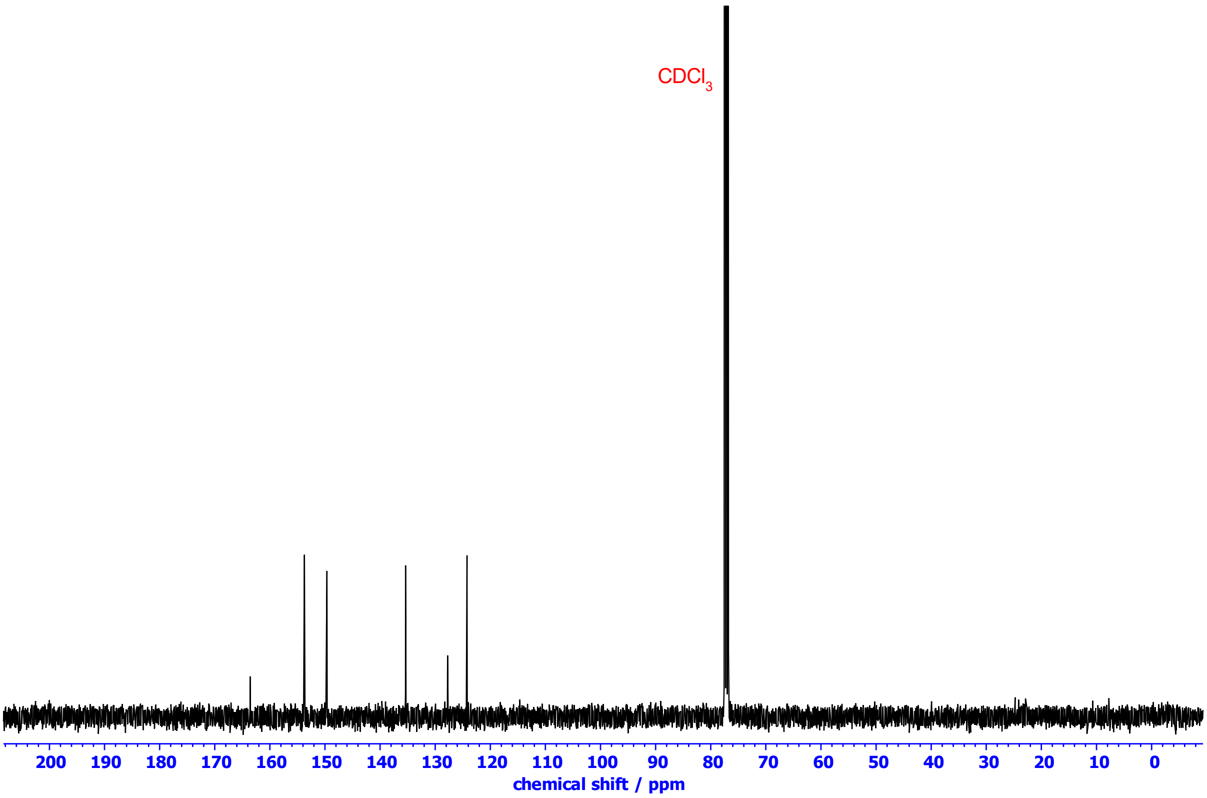

Figure S5: ^{13}C NMR spectrum of **L^1**
(101 MHz, 298 K, $CDCl_3$)

S4.2 Synthesis of 3,6-di(pyridin-3-yl)pyridazine L²

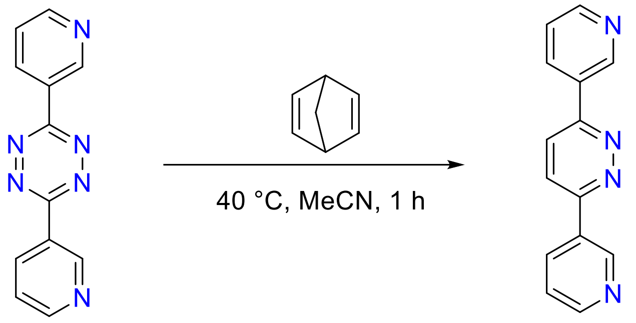

L¹ 94% **L²**

Tetrazine **L¹** (30.0 mg, 140 µmol) was dissolved in norbornadiene (38.7 mg, 420 µmol, 43 µL) and added to MeCN (0.6 mL) and the mixture stirred at 40 ˚C for 1 h. The mixture was then centrifuged (5 min, 3000 rpm), the liquid decanted. Ice cold MeCN (3 × 1.5 mL) was then added, and the mixture centrifuged (5 min, 3000 rpm) and the solvent decanted. The remaining solid was dried on a vacuum line to give pyridazine **L²** as a pale-brown powder (27.8 mg, 128 µmol, 94%).

^{1}H NMR (400 MHz, $CDCl_3$) δ_H: 9.31 (d, *J* = 1.5 Hz, 2H), 8.77 (dd, *J* = 4.9, 1.7 Hz, 2H), 8.56 (dt, *J* = 8.0, 2.0 Hz, 2H), 8.04 (s, 2H), 7.51 (dd, *J* = 8.0, 4.9 Hz, 2H).

^{1}H NMR (400 MHz, CD_3CN) δ_H: 9.35 (d, *J* = 2.3 Hz, 2H), 8.73 (dd, *J* = 4.9, 1.6 Hz, 2H), 8.52 (dt, *J* = 8.0, 2.0 Hz, 2H), 8.20 (s, 2H), 7.55 (dd, *J* = 8.0, 4.8 Hz, 2H).

^{13}C NMR (101 MHz, $CDCl_3$) δ_C: 156.2, 151.4, 148.2, 134.6, 131.8, 124.1, 124.1.

ESI-MS (ESI, MeCN), *m/z*: calculated for $[M+H]^+$, $[C_{14}H_{11}N_4]^+$, 235.0978, found 235.0976.

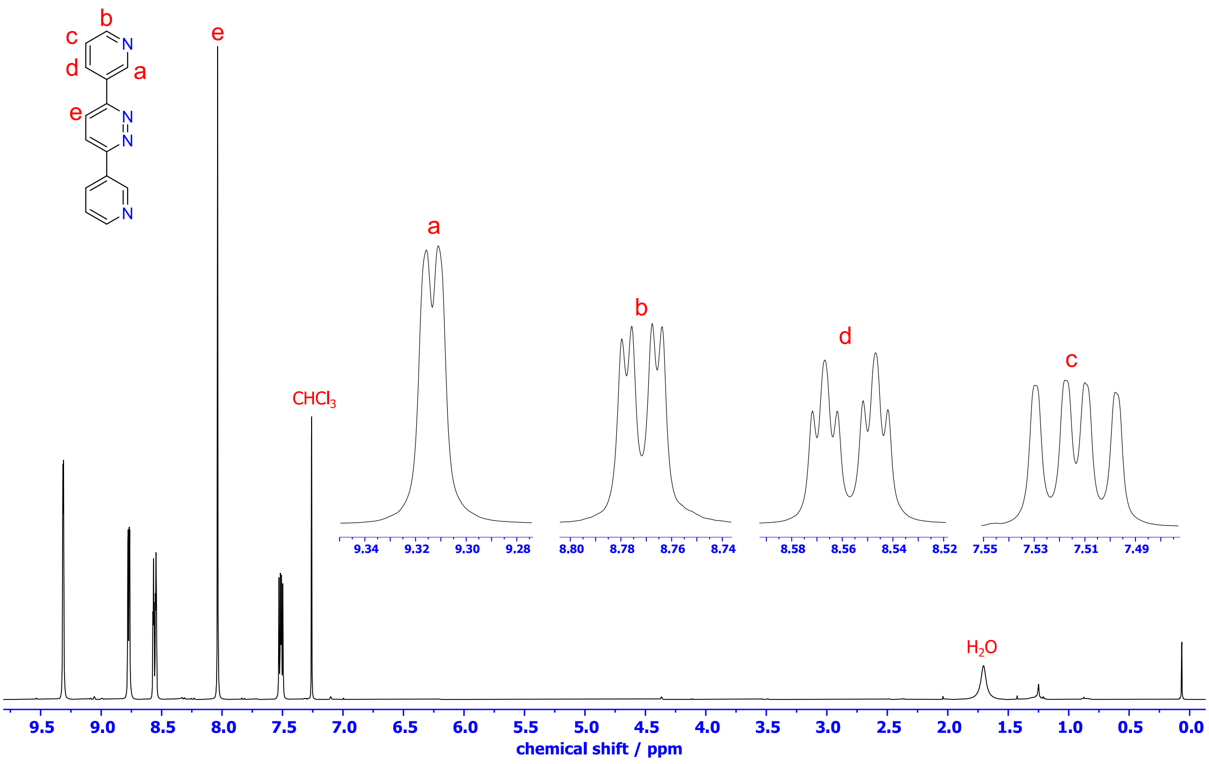

Figure S6: ^{1}H NMR spectrum of $\mathbf{L^2}$
(400 MHz, 298 K, $CDCl_3$)

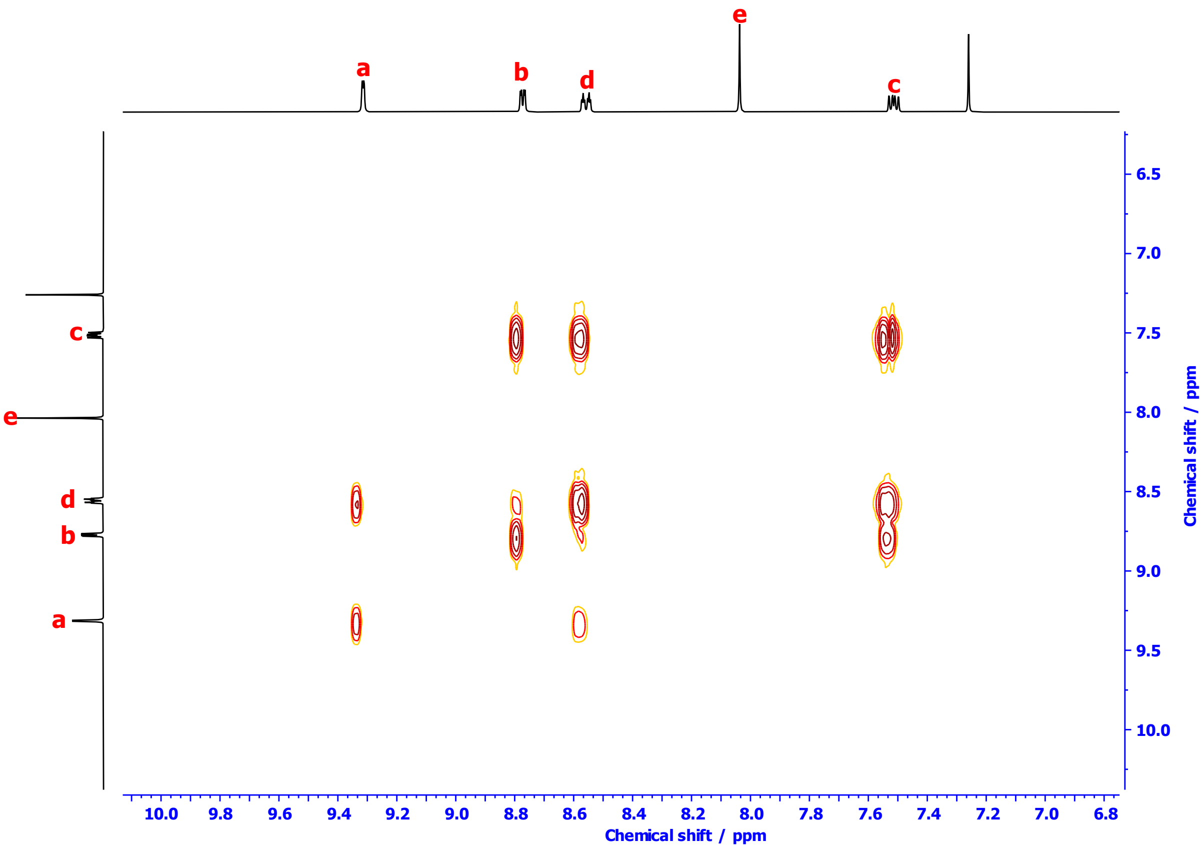

Figure S7: ^{1}H-^{1}H COSY spectrum of $\mathbf{L^2}$
(400 MHz, 298 K, $CDCl_3$)

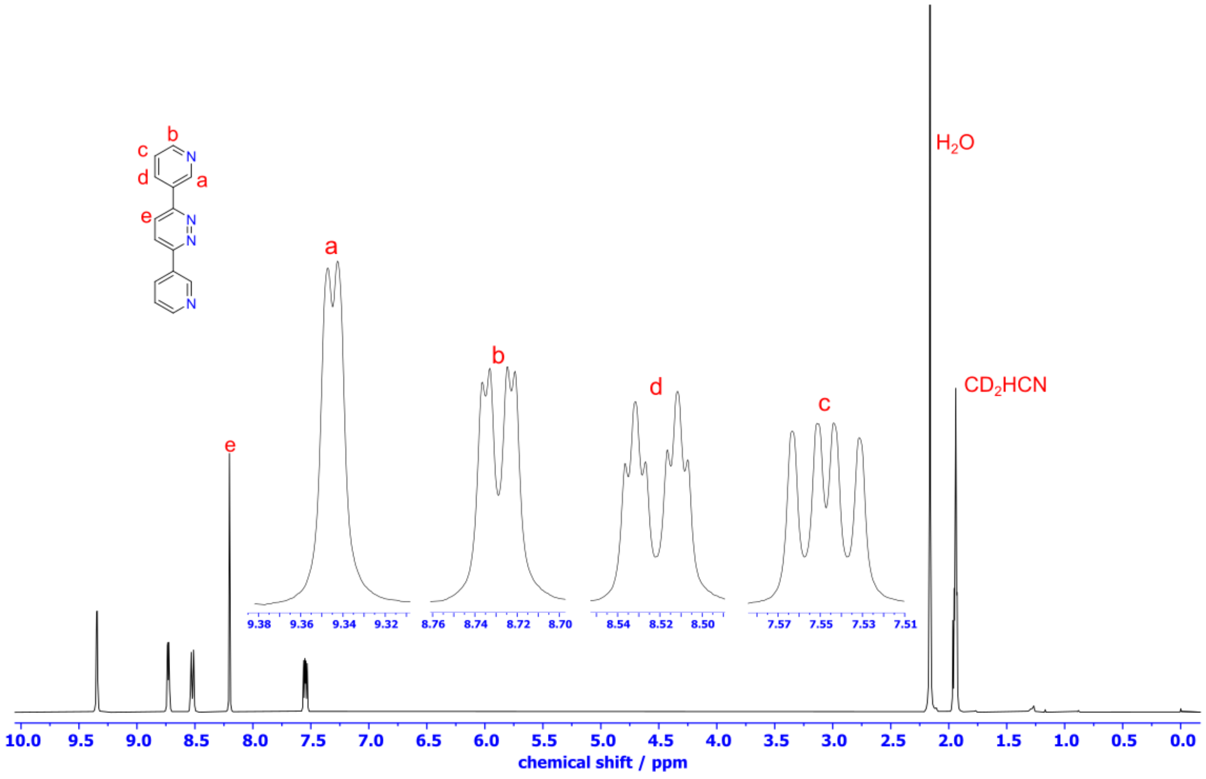

Figure S8: ^{1}H NMR spectrum of **L^{2}**
(400 MHz, 298 K, CD_3CN)

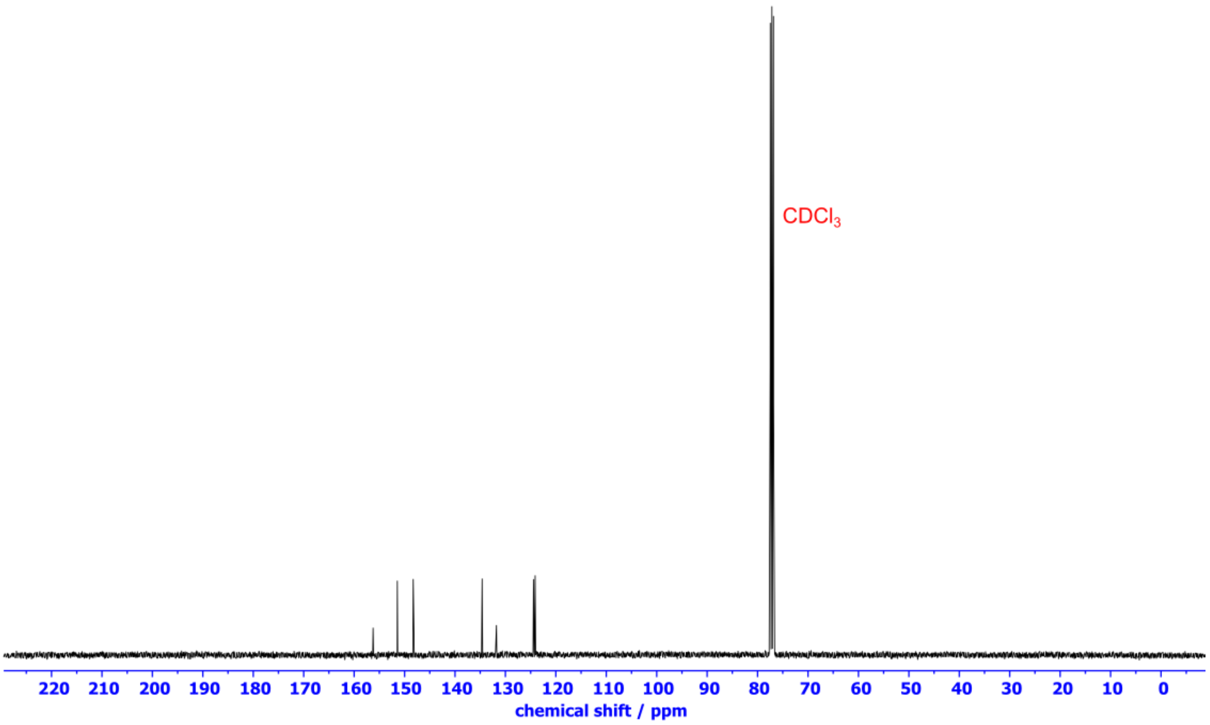

Figure S9: ^{13}C NMR spectrum of **L^{2}**
(101 MHz, 298 K, $CDCl_3$)

S4.3 Synthesis of 1,4-di(pyridin-3-yl)-6,7-dihydro-5H-cyclopenta[d]pyridazine L[6]

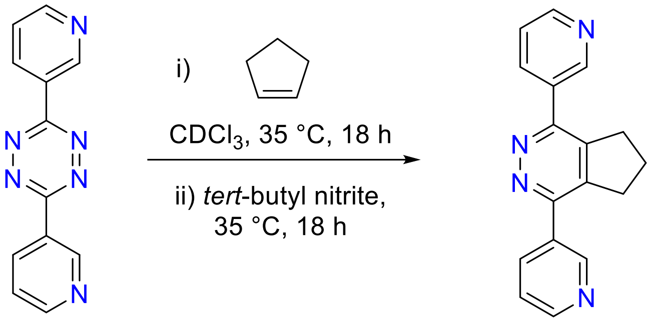

L[1] **L[6]**

Cyclopentene (17.0 mg, 250 µmol, 22.0 µL) was added to a solution of tetrazine **L[1]** (20 mg, 84.7 µmol) in $CDCl_3$ (0.6 mL) and heated at 35 °C for 18 h to give a yellow solution. To this was added *tert*-butyl nitrite (10.5 mg, 0.012 mL, 102 µmol) and the mixture was heated to 35 °C for 18 h. The solvent was then removed under reduced pressure and then washed with Et_2O (3 × 5 mL) and the precipitate dried *in vacuo* to give 1,4-di(pyridin-3-yl)-6,7-dihydro-5H-cyclopenta[d]pyridazine **L[6]** as a brown powder (22.8 mg, 83.0 µmol, 98%).

^{1}H NMR (400 MHz, $CDCl_3$) δ_H: 9.12 (d, J = 2.2 Hz, 2H), 8.72 (dd, J = 5.0, 1.6 Hz, 2H), 8.33 (dt, J = 7.9, 2.0 Hz, 2H), 7.49 (dd, J = 8.0, 4.9 Hz, 2H), 3.26 (t, J = 7.4 Hz, 4H), 2.21 (p, J = 7.5 Hz, 2H).

^{13}C NMR (101 MHz, $CDCl_3$) δ_C: 154.4, 150.5, 149.5, 143.7, 136.2, 132.8, 123.8, 33.0, 25.1.

ESI-MS (ESI, MeCN), *m/z*: calculated for $[M+H]^+$, $[C_{17}H_{15}N_4]^+$, 275.1291, found 275.1296.

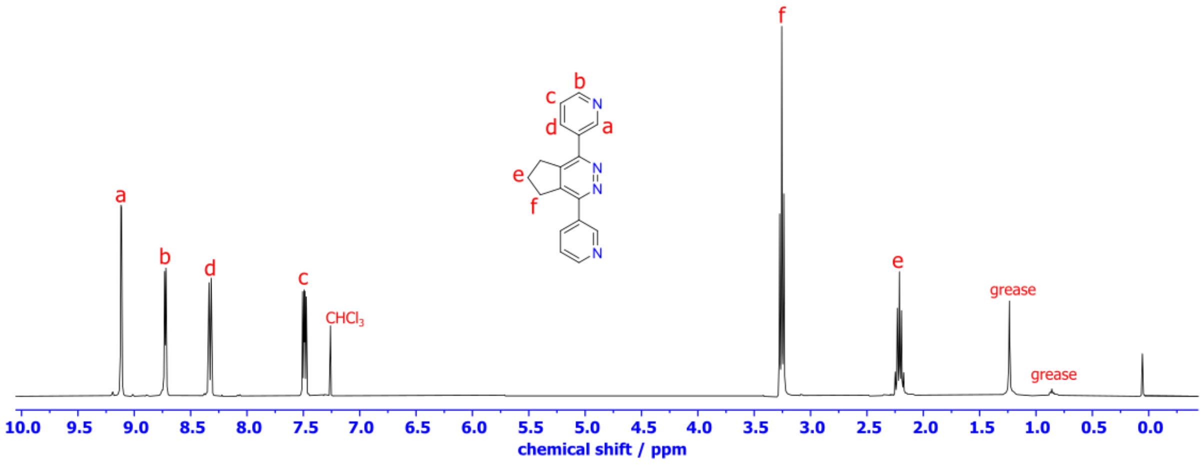

Figure S10: ^{1}H NMR spectrum of **L[6]**
(400 MHz, 298 K, $CDCl_3$)

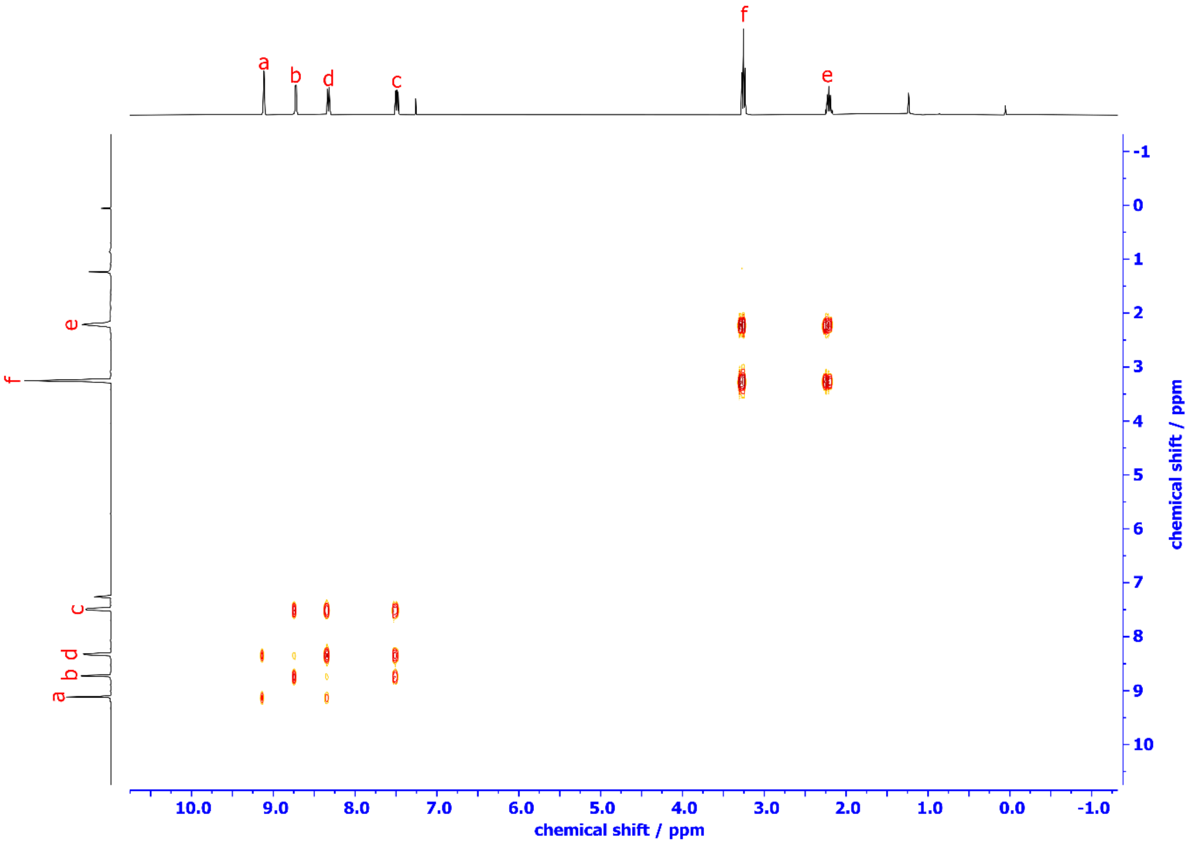

Figure S11: ^{1}H-^{1}H COSY spectrum of $\mathbf{L^6}$
(400 MHz, 298 K, $CDCl_3$)

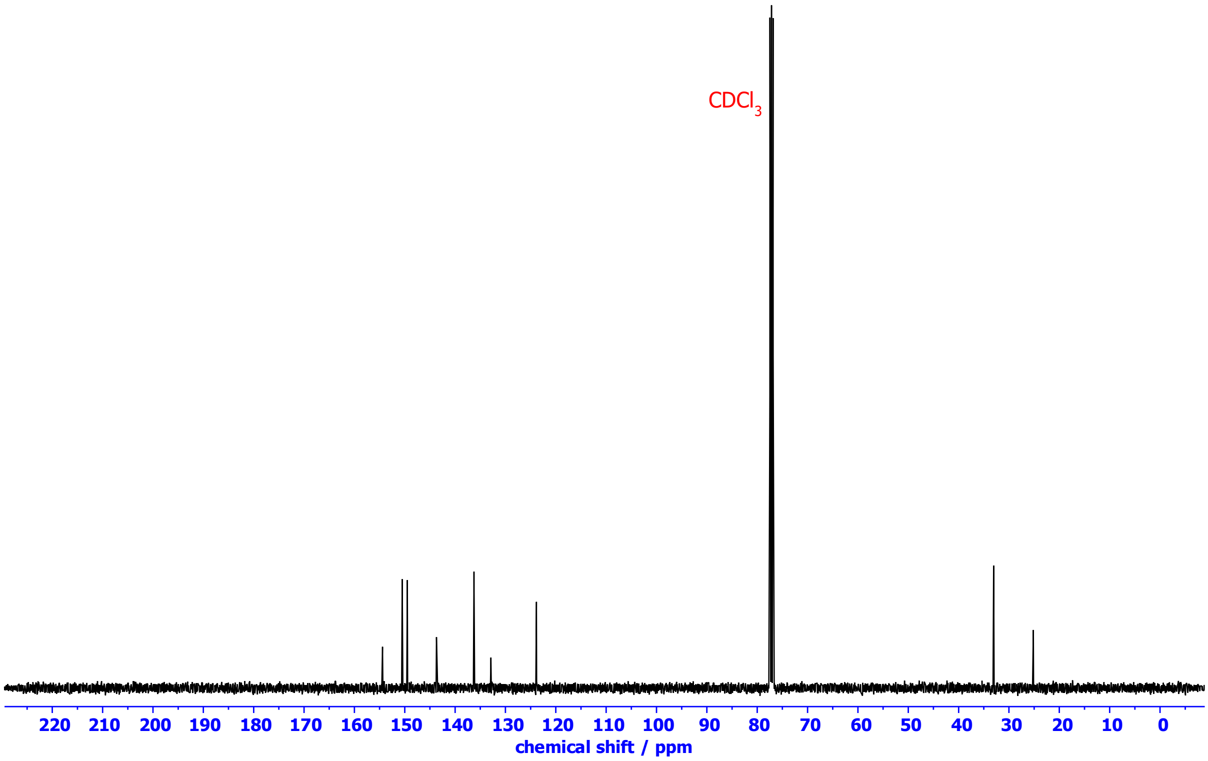

Figure S12: ^{13}C NMR spectrum of $\mathbf{L^6}$
(101 MHz, 298 K, $CDCl_3$)

S4.4 Synthesis of 1,4-di(pyridin-3-yl)-5,6,7,8-tetrahydro-5,8-methanophthalazine L[7]

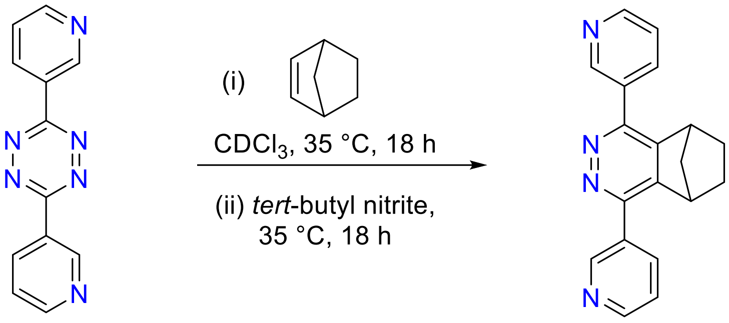

L¹ **L⁷**

Investigations into the *in situ* oxidation of norbornyl-ligand (**L³**) based lantern **3** and tetrahedron **3T** only gave insoluble precipitate upon oxidation. Ligand **L⁷** was synthesized separately to test whether the pyridazine product structures from this oxidized ligand could be accessed via direct self-assembly, and this synthetic procedure is reported here. Unfortunately when **L⁷** was mixed with $[Pd(CH_3CN)_4](BF_4)_2$ in MeCN an insoluble precipitate was formed, indicating that this product structure was inaccessible via direct assembly or post-assembly modification.

Norbornene (15.9 mg, 169 µmol) was added to a solution of tetrazine **L¹** (20 mg, 84.7 µmol) in $CDCl_3$ (1 mL) and the mixture heated to 35 °C for 18 h. To the yellow solution was added *tert*-butyl nitrite (10.5 mg, 0.012 mL, 102 µmol) and the solution was heated for a further 18 h. The solvent was then removed *in vacuo* and the crude product washed with Et_2O (3 × 3 mL) and hexane (3 × 5mL) to give 1,4-di(pyridin-3-yl)-5,6,7,8-tetrahydro-5,8-methanophthalazine **L⁷** (24.7 mg, 82.2 µmol, 97%) as a yellow powder.

^{1}H NMR (400 MHz, DMSO-*d*$_6$) δ_H: 9.28 (d, *J* = 2.1 Hz, 2H), 8.97 (dd, *J* = 5.4, 1.4 Hz, 2H), 8.72 (dt, *J* = 8.1, 1.8 Hz, 2H), 8.03 (dd, *J* = 8.1, 5.3 Hz, 2H), 3.80 (s, 2H) , 2.21 (d, *J* = 8.8 Hz, 2H), 1.85 (d, *J* = 9.5 Hz, 1H), 1.72 (d, *J* = 9.3 Hz, 1H), 1.46 (d, *J* = 7.6, 2.2 Hz, 2H).

^{13}C NMR (101 MHz, DMSO-*d*$_6$) δ_C: 149.7, 147.0, 146.1, 145.0, 141.2, 133.6, 126.0, 49.2, 41.5, 24.7

ESI-MS (ESI, DMSO), *m/z*: calculated for $[M+H]^+$, $[C_{19}H_{17}N_4]^+$, 301.1448, found 301.1452.

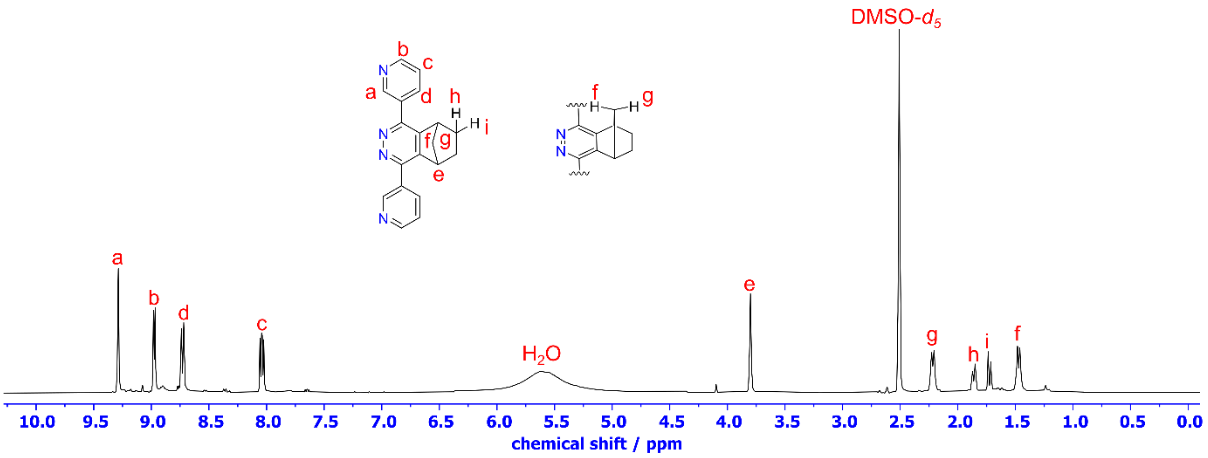

Figure S13: ^{1}H NMR spectrum of **L^{7}**
(400 MHz, 298 K, DMSO-d_6)

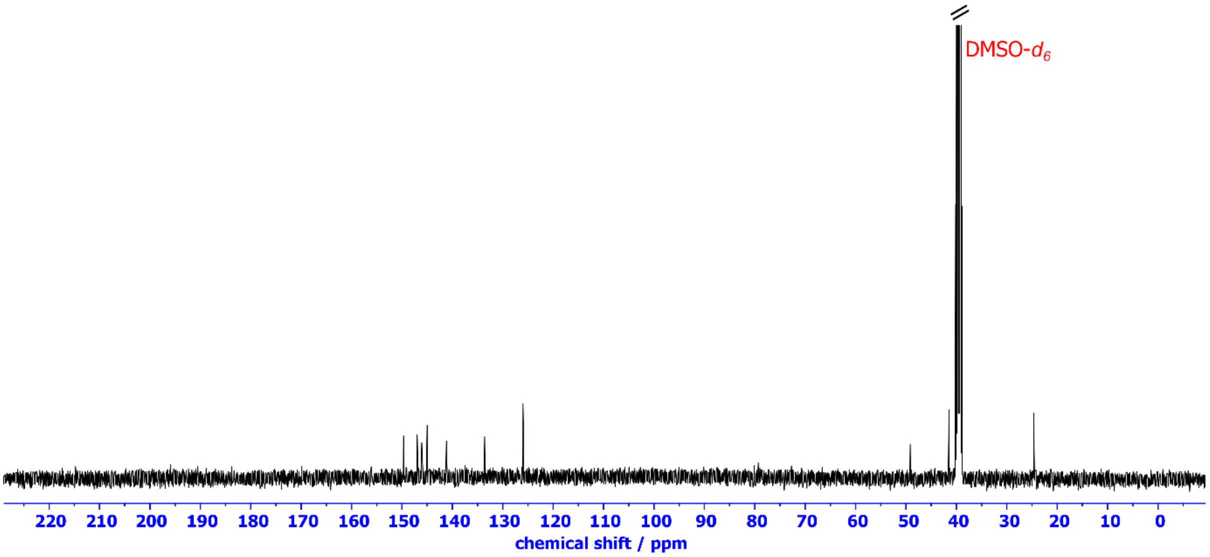

Figure S14: ^{13}C NMR spectrum of **L^{7}**
(101 MHz, 298 K, DMSO-d_6)

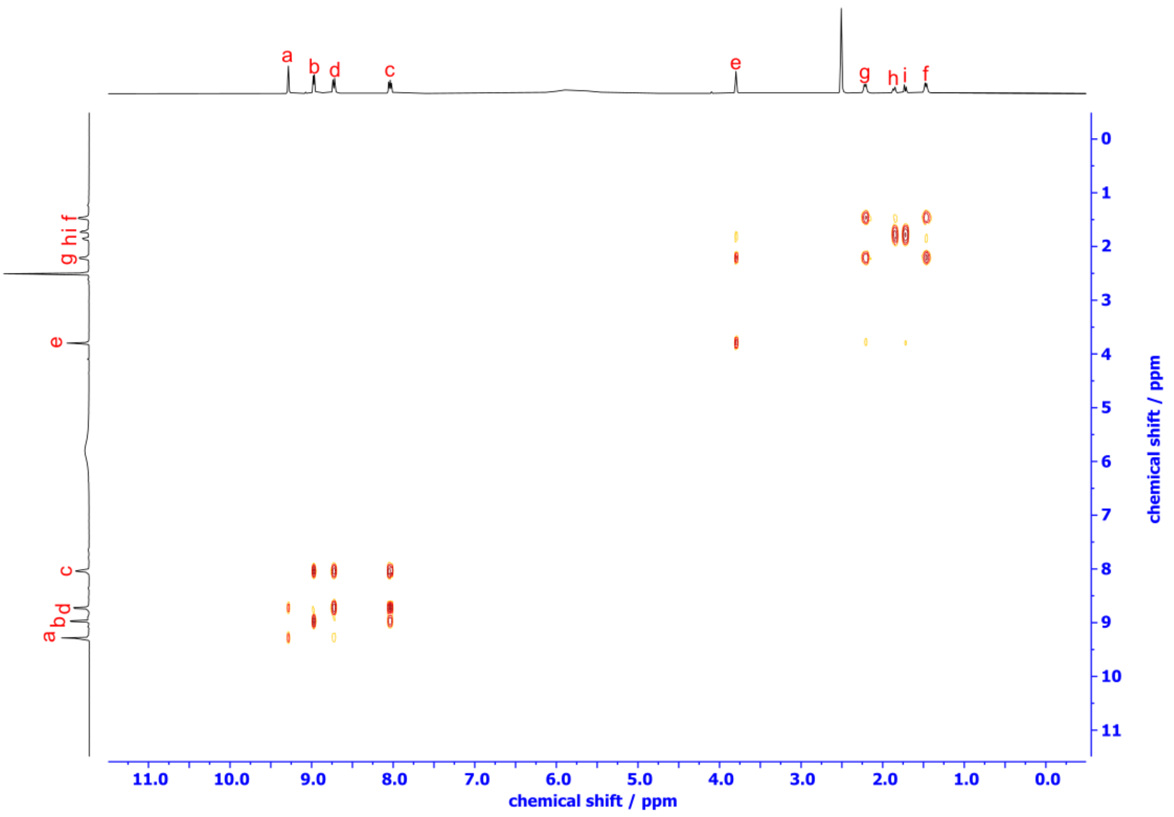

Figure S15: ^{1}H-^{1}H COSY spectrum of $\mathbf{L^7}$
(400 MHz, 298 K, DMSO-d_6)

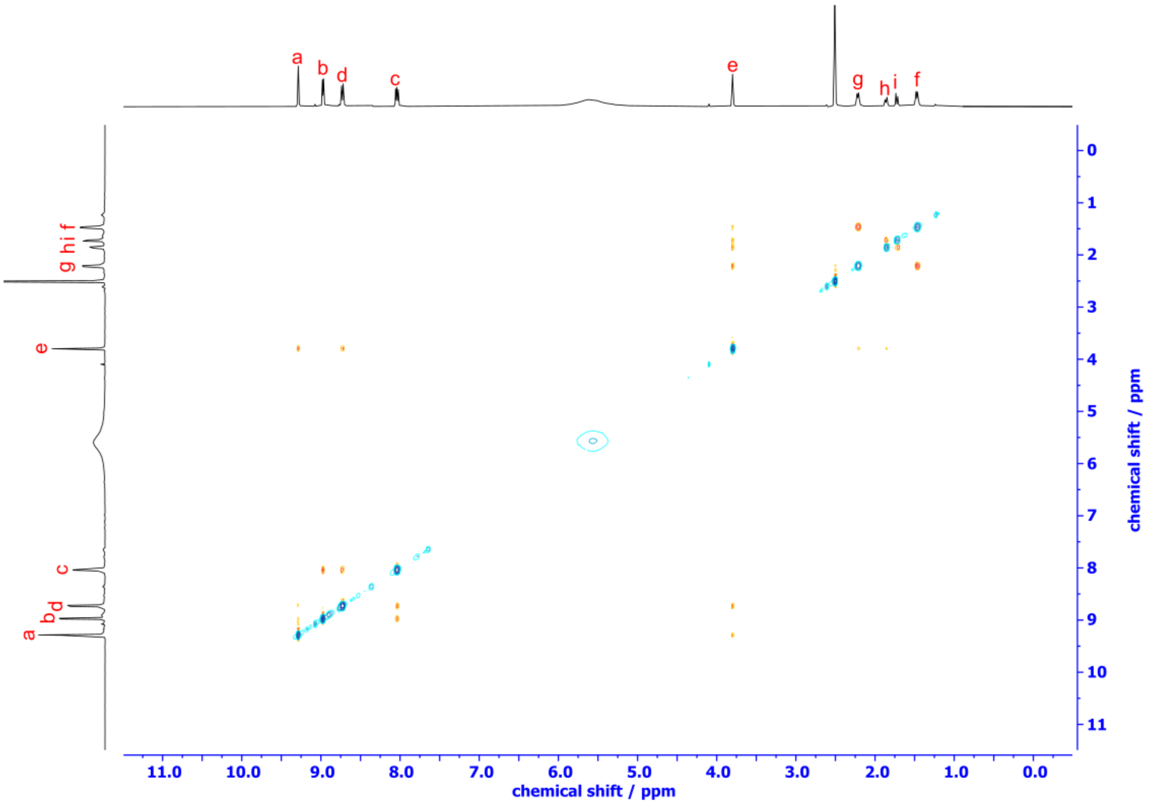

Figure S16: ^{1}H-^{1}H NOESY spectrum of $\mathbf{L^7}$
(400 MHz, 298 K, DMSO-d_6)

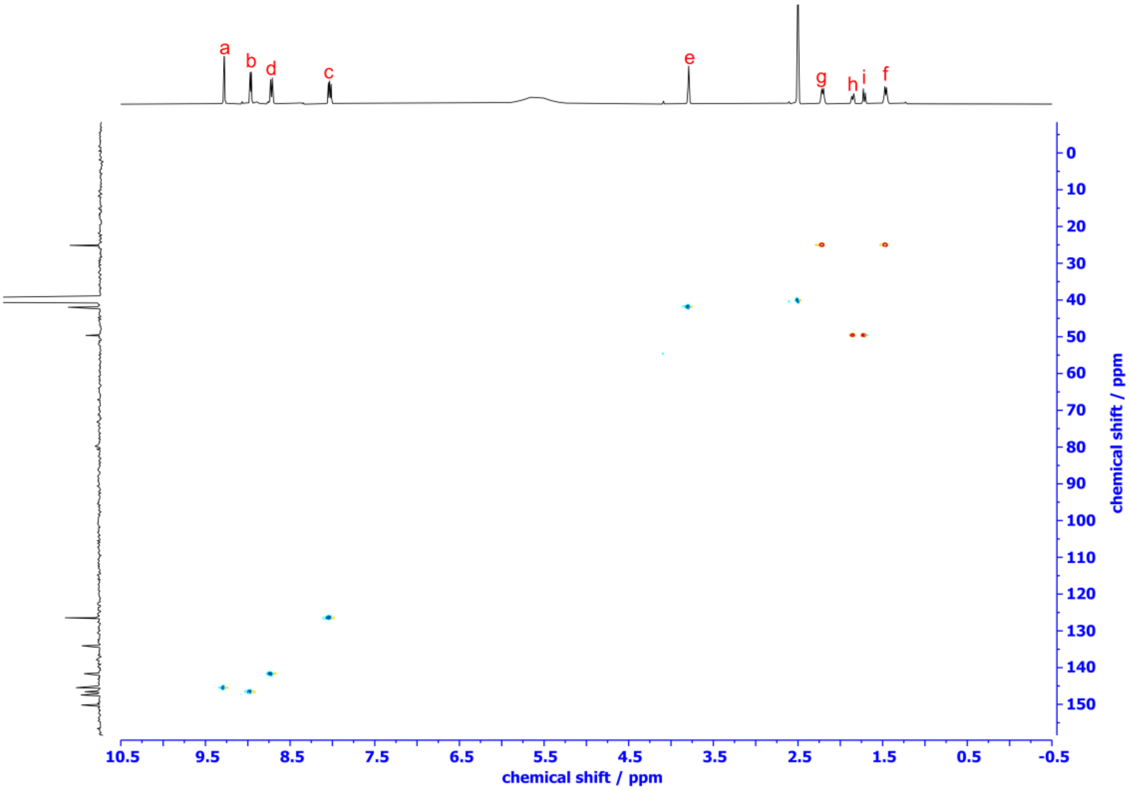

Figure S17: ^{1}H-^{13}C HSQC spectrum of **L**$^{\mathbf{7}}$
(400 MHz, 298 K, DMSO-d_6)

S5 Synthesis and Characterisation of Metal-Organic Cages

S5.1 Self-assembly of tetrahedral cage [1](BF_4)$_8$ / [$Pd_4L^1_8$](BF_4)$_8$

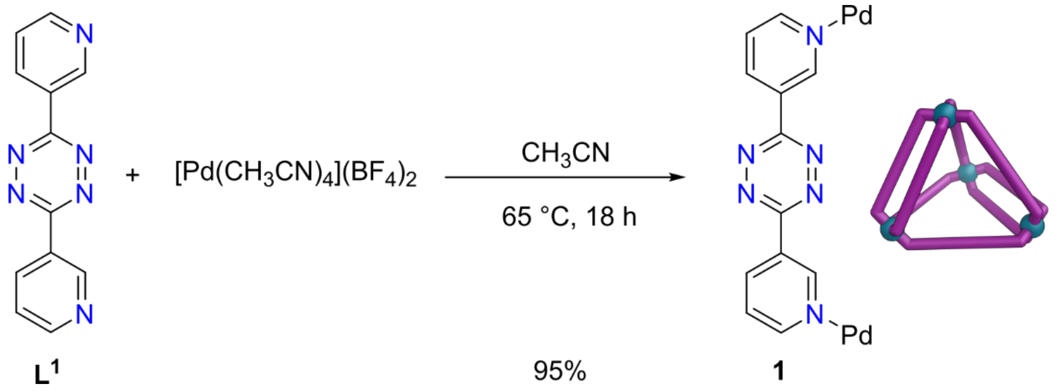

3,6-Di(pyridin-3-yl)tetrazine **L¹** (23.6 mg, 100 µmol) was dissolved in CH_3CN (4.5 mL). To this [Pd(CH_3CN)$_4$](BF_4)$_2$ (22.2 mg, 50.0 µmol) was added. The mixture was heated to 65 °C for 18 h, the solvent was then removed *in vacuo* and the solid washed with Et_2O (2 × 10 mL) to afford a pink powder of tetrahedral cage **1** (43.4 mg, 14.4 µmol, 95%).

^{1}H NMR (400 MHz, CD_3CN) δ_H: 10.88 (d, J = 2.0 Hz, 8H), 10.40 (d, J = 1.9 Hz, 8H), 9.73 (dd, J = 5.8, 1.4 Hz, 8H), 9.17 (dd, J = 5.9, 1.4 Hz, 8H), 9.11 (dt, J = 8.3, 1.6 Hz, 8H), 8.92 (dt, J = 8.2, 1.6 Hz, 8H), 8.11 (dd, J = 8.3, 5.7 Hz, 8H), 7.72 (dd, J = 8.2, 5.7 Hz, 8H).

^{13}C NMR (101 MHz, CD_3CN) δ_C: 163.4, 162.5, 155.6, 154.8, 153.0, 150.9, 141.1, 139.9, 132.8, 132.7, 129.6, 129.0.

ESI-MS (ESI, CD_3CN), *m/z*: [**1**(BF_4)$_4$]$^{4+}$ calculated 665.5742, found 665.5703; [**1**(BF_4)$_3$]$^{5+}$ calculated 515.0581, found 515.0570; [**1**(BF_4)$_2$]$^{6+}$ calculated 414.7140, found 414.7139; [**1**(BF_4)]$^{7+}$ calculated 343.1825, found 343.1814.

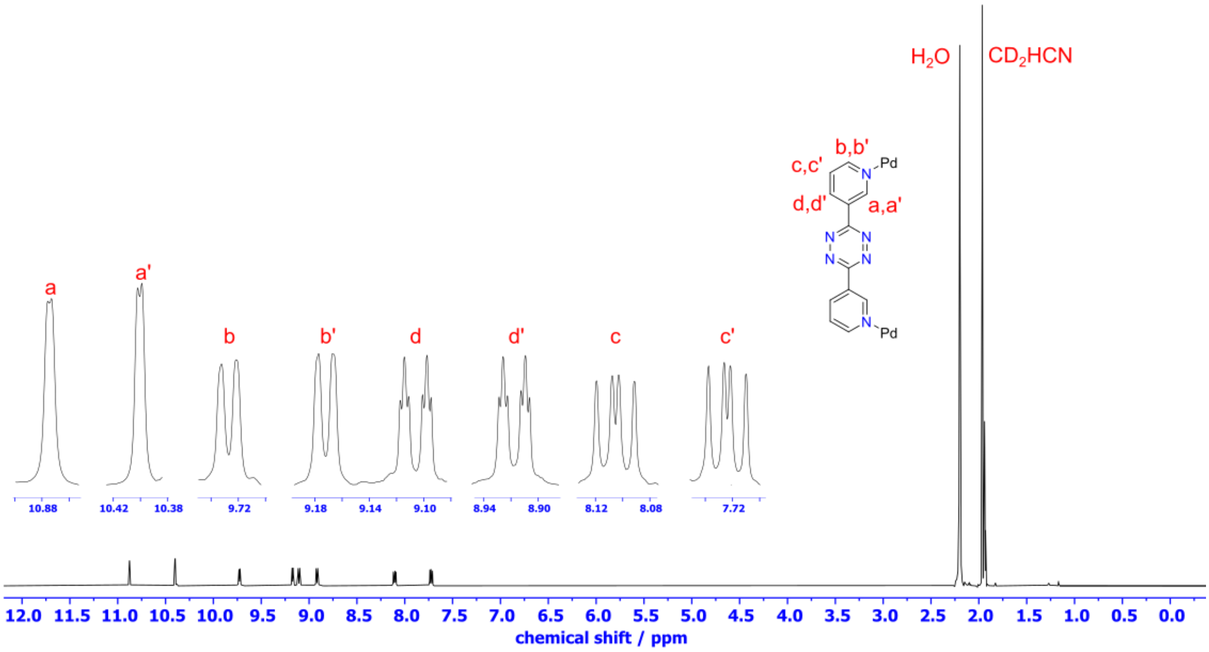

Figure S18: ^{1}H NMR spectrum of tetrahedral cage **1**
(500 MHz, 298 K, CD_3CN)

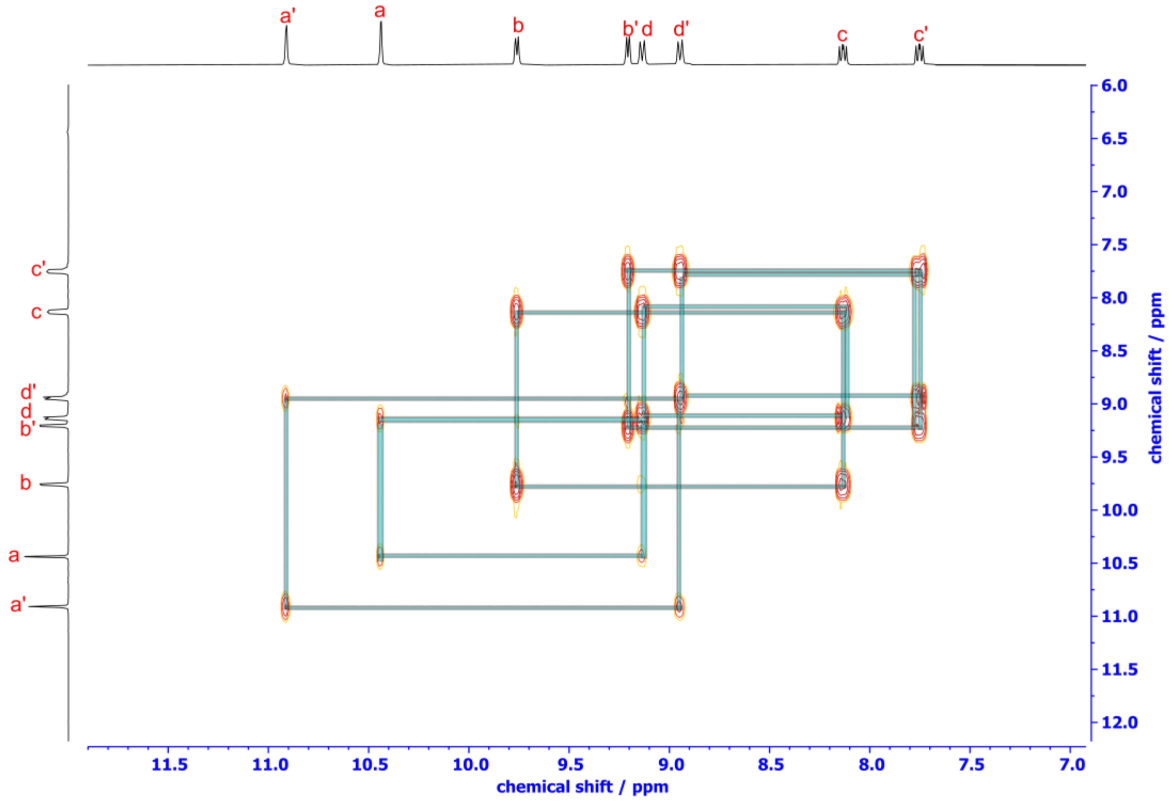

Figure S19: ^{1}H-^{1}H COSY spectrum of tetrahedral cage **1**
(500 MHz, 298 K, CD_3CN)

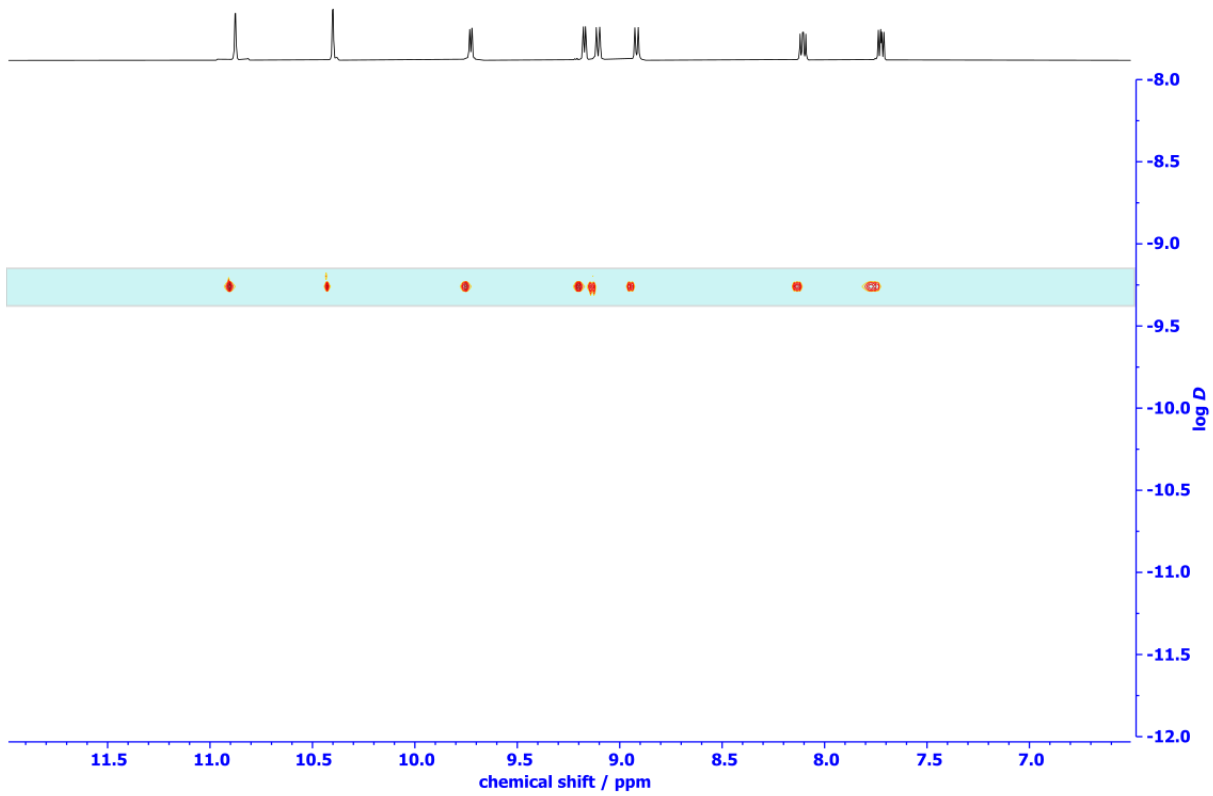

Figure S20: ^{1}H DOSY spectrum of tetrahedral cage **1**
(500 MHz, 298 K, CD_3CN)

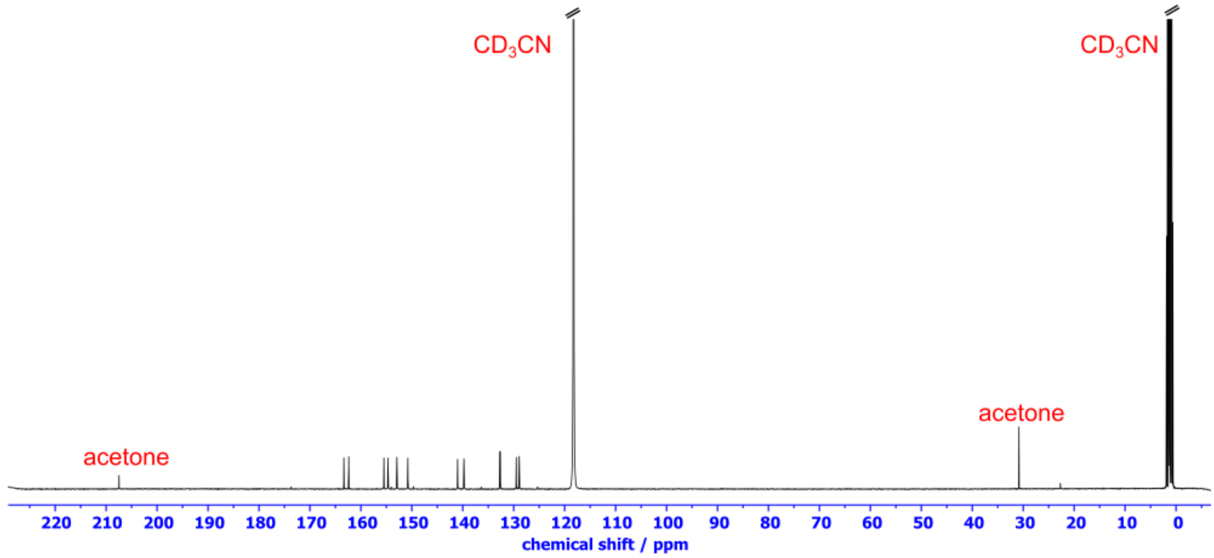

Figure S21: ^{13}C NMR spectrum of tetrahedral cage **1**
(101 MHz, 298 K, CD_3CN)

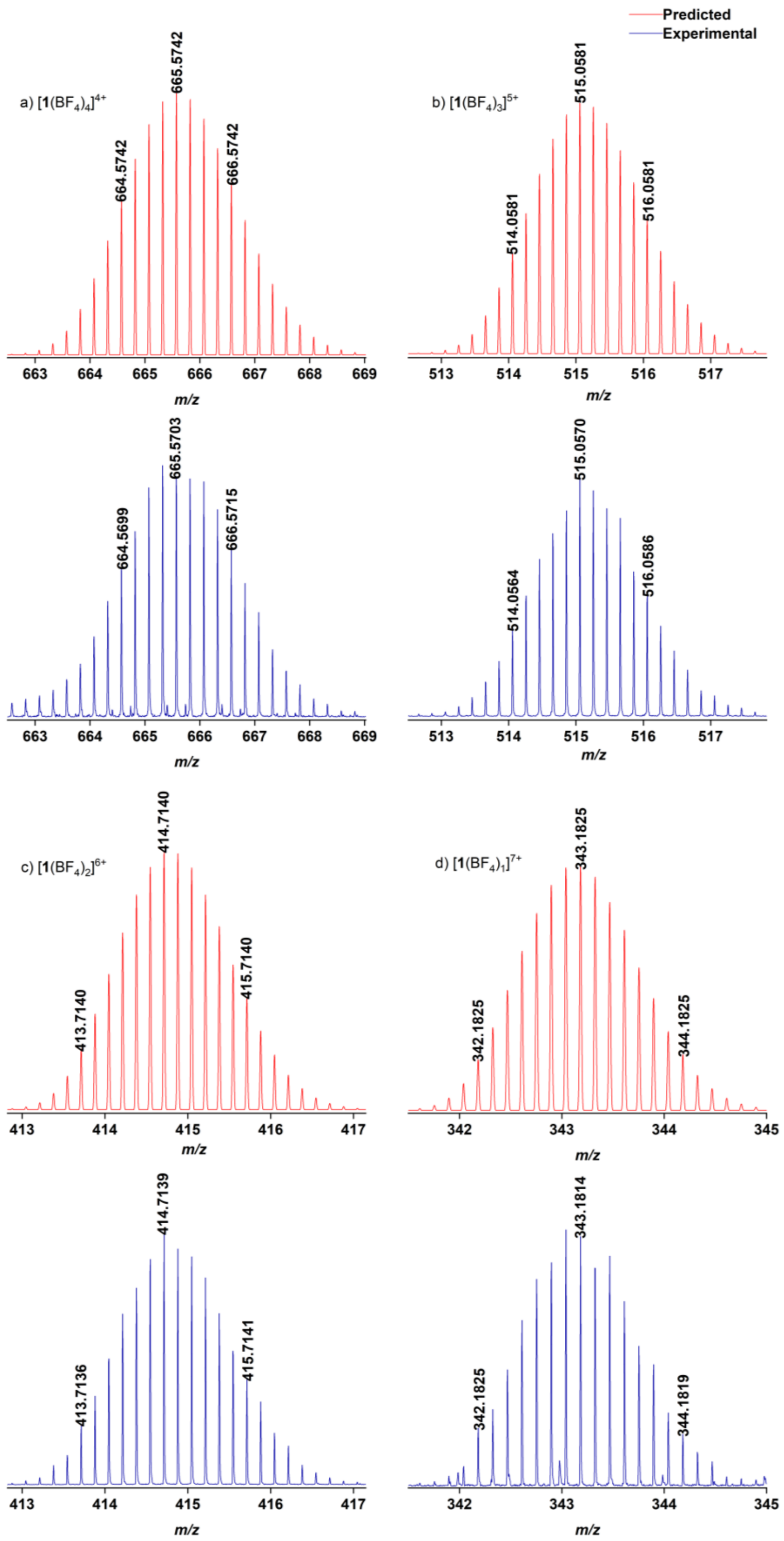

Figure S22: Isotopic distribution of selected peaks from tetrahedral cage **1** showing match between predicted and experimentally observed patterns (a) [**1**$(BF_4)_4]^{4+}$, (b) [**1**$(BF_4)_3]^{5+}$, (c) [**1**$(BF_4)_2]^{6+}$, (d) [**1**$(BF_4)]^{7+}$.

S5.2 Self-assembly of tetrahedral cage [2](BF_4)$_8$ / [$Pd_4L^2{}_8$](BF_4)$_8$

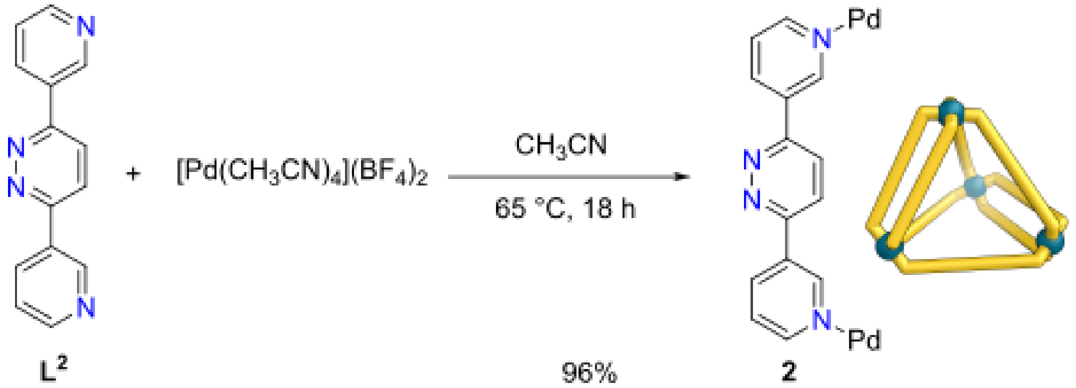

3,6-Di(pyridin-3-yl)pyridazine **L²** (9.37 mg, 40.0 µmol) was dissolved in CH_3CN (1.5 mL). To this [Pd(CH_3CN)$_4$](BF_4)$_2$ (8.88 mg, 20.0 µmol) was added. The mixture was heated to 65 °C for 18 h, the solvent was then removed *in vacuo* and the solid washed with Et_2O (2 × 10 mL) to afford a pale-yellow powder of **2** (17.5 mg, 5.85 µmol, 96%).

^{1}H NMR (500 MHz, CD_3CN) δ_H: 10.53 (d, *J* = 2.0 Hz, 8H), 9.85 (d, *J* = 2.0 Hz, 8H), 9.51 (dd, *J* = 5.7, 1.4 Hz, 8H), 9.05 (dd, *J* = 5.7, 1.3 Hz, 8H), 8.94 (dt, *J* = 8.2, 1.5 Hz, 8H), 8.58 (s, 8H), 8.45 (dt, *J* = 8.1, 1.4 Hz, 8H), 8.29 (s, 8H), 7.96 (dd, *J* = 8.2, 5.7 Hz, 8H), 7.64 (dd, *J* = 8.1, 5.8 Hz, 8H).

^{13}C NMR (126 MHz, CD_3CN) δ_C: 155.6, 155.5, 153.0, 152.8, 151.8, 150.1, 140.0, 139.4, 136.4, 136.1, 129.2, 128.3, 126.9, 126.5.

ESI-MS (ESI, MeCN), *m/z*: [**2**(BF_4)$_5$]$^{3+}$ calculated 911.1265, found 911.1179; [**2**(BF_4)$_4$]$^{4+}$ calculated 661.5932, found 661.5903.

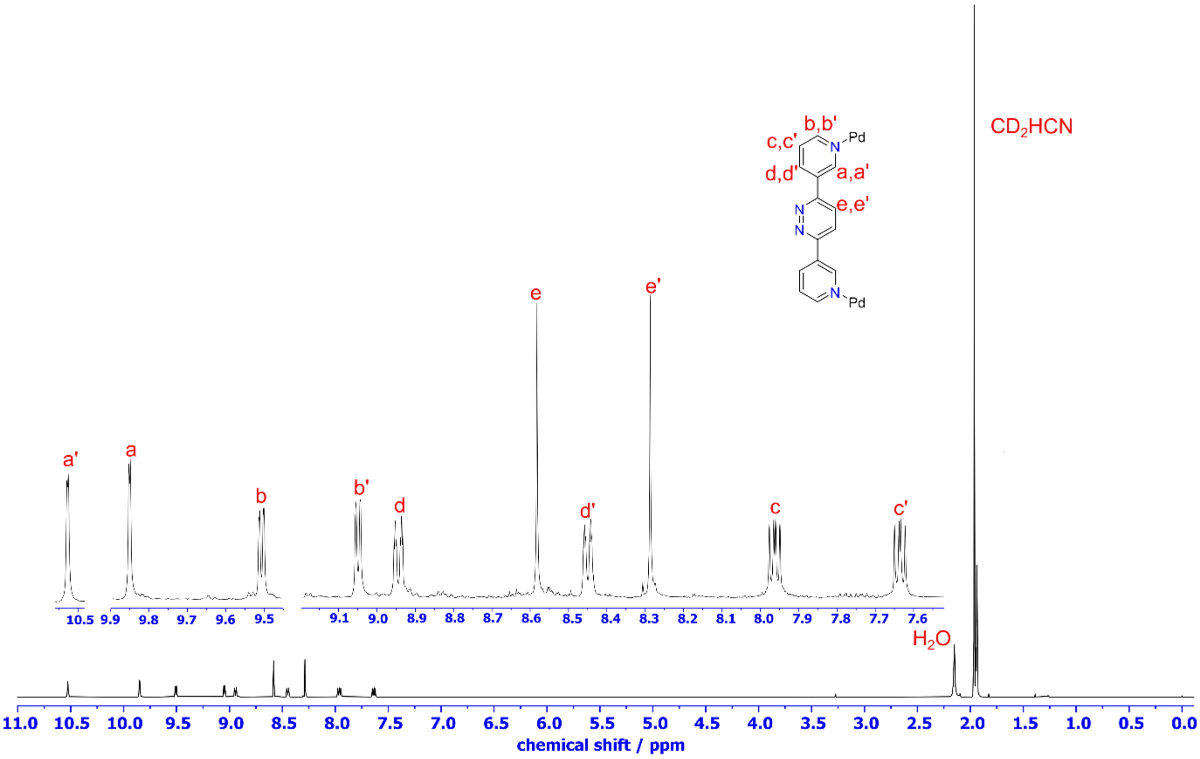

Figure S23: ^{1}H NMR spectrum of tetrahedral cage **2**
(500 MHz, 298 K, CD_3CN)

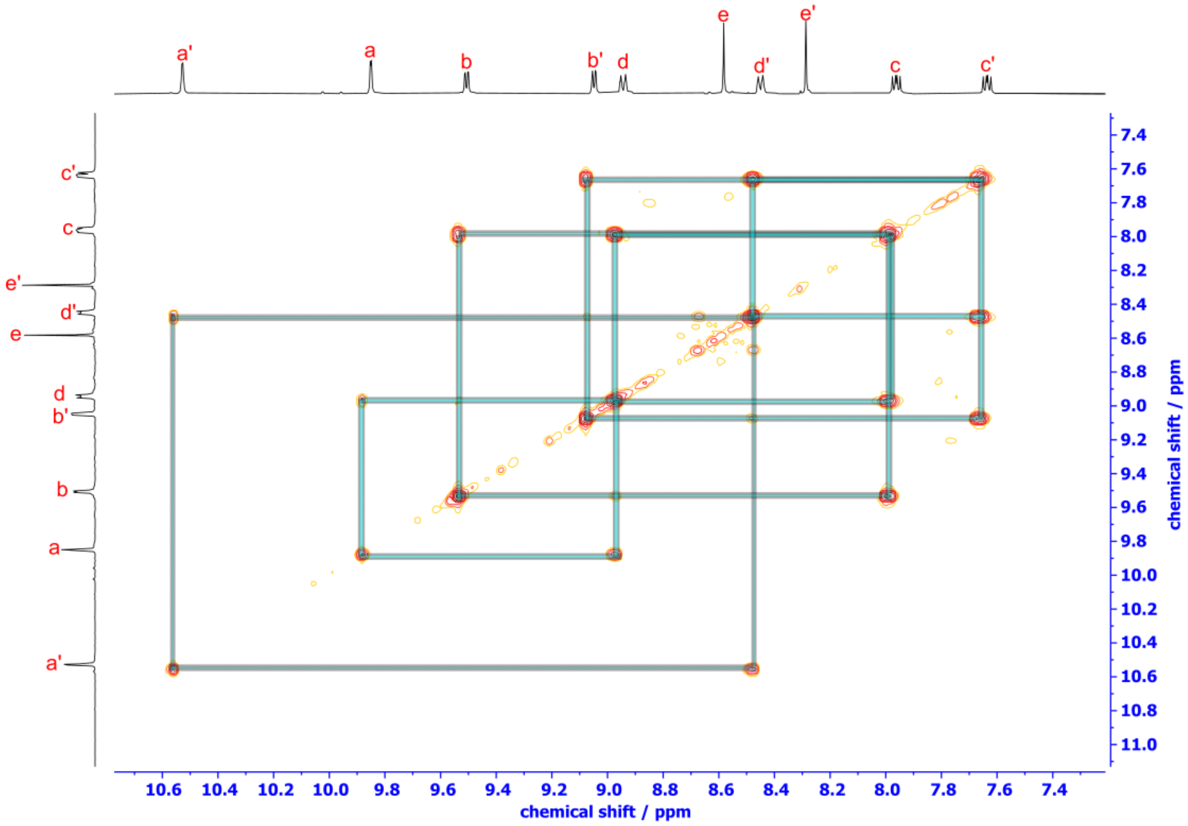

Figure S24: ^{1}H-^{1}H COSY spectrum of tetrahedral cage **2**
(500 MHz, 298 K, CD_3CN)

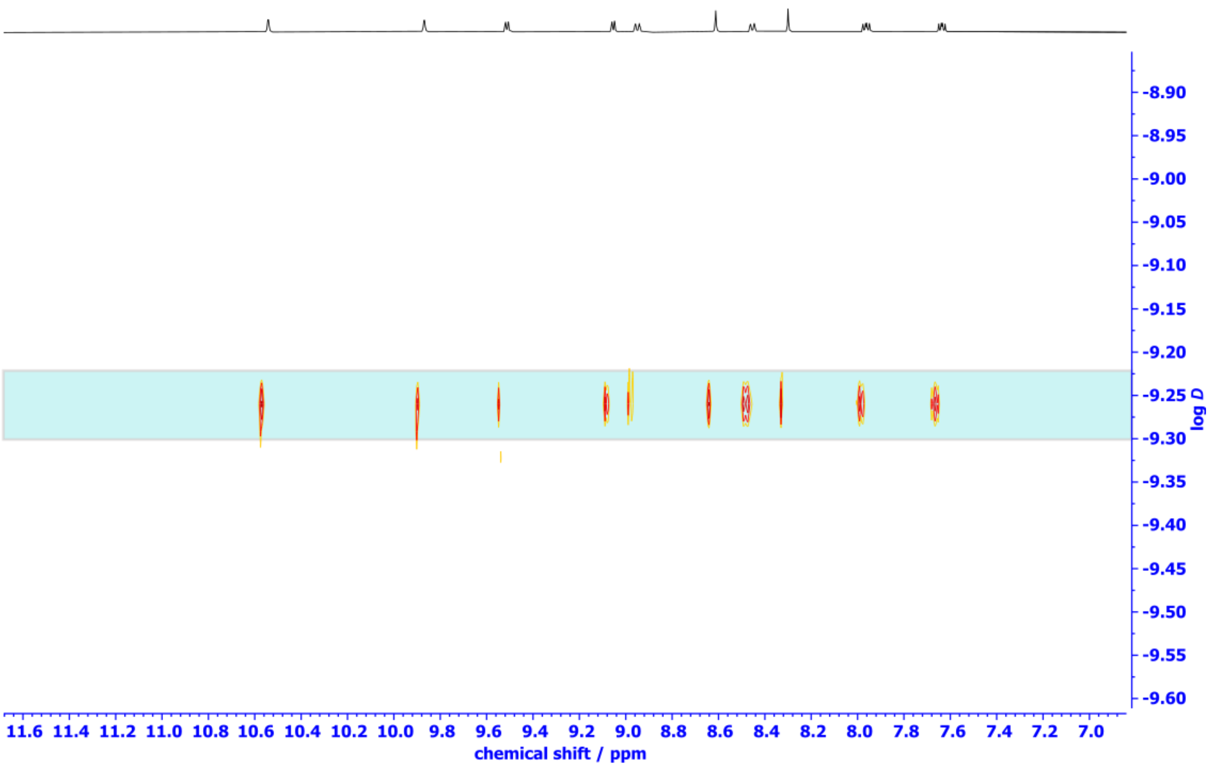

Figure S25: ^{1}H-DOSY spectrum of tetrahedral cage **2**
(500 MHz, 298 K, CD_3CN)

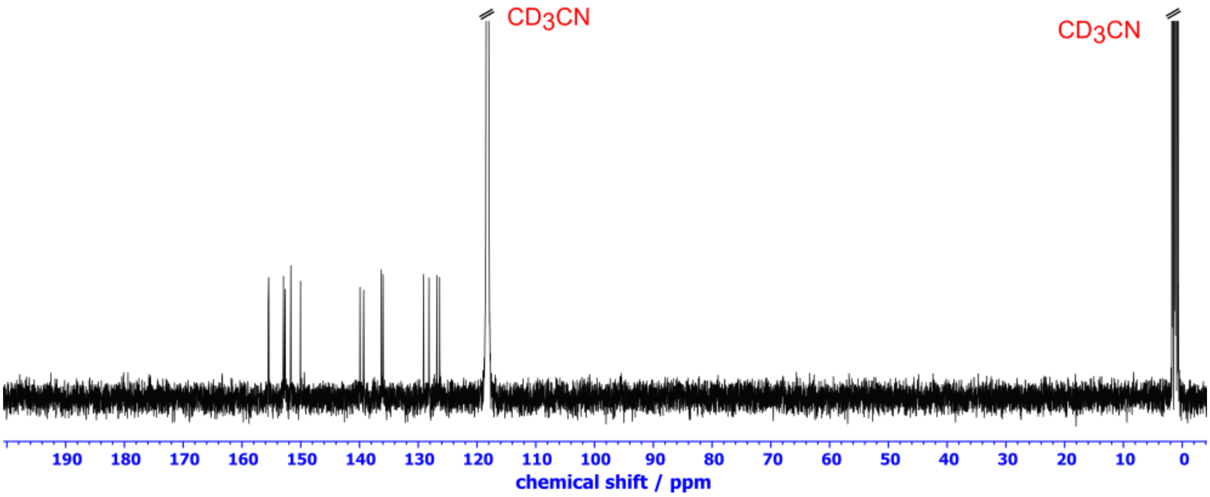

Figure S26: ^{13}C NMR spectrum of tetrahedral cage **2**
(126 MHz, 298 K, CD_3CN)

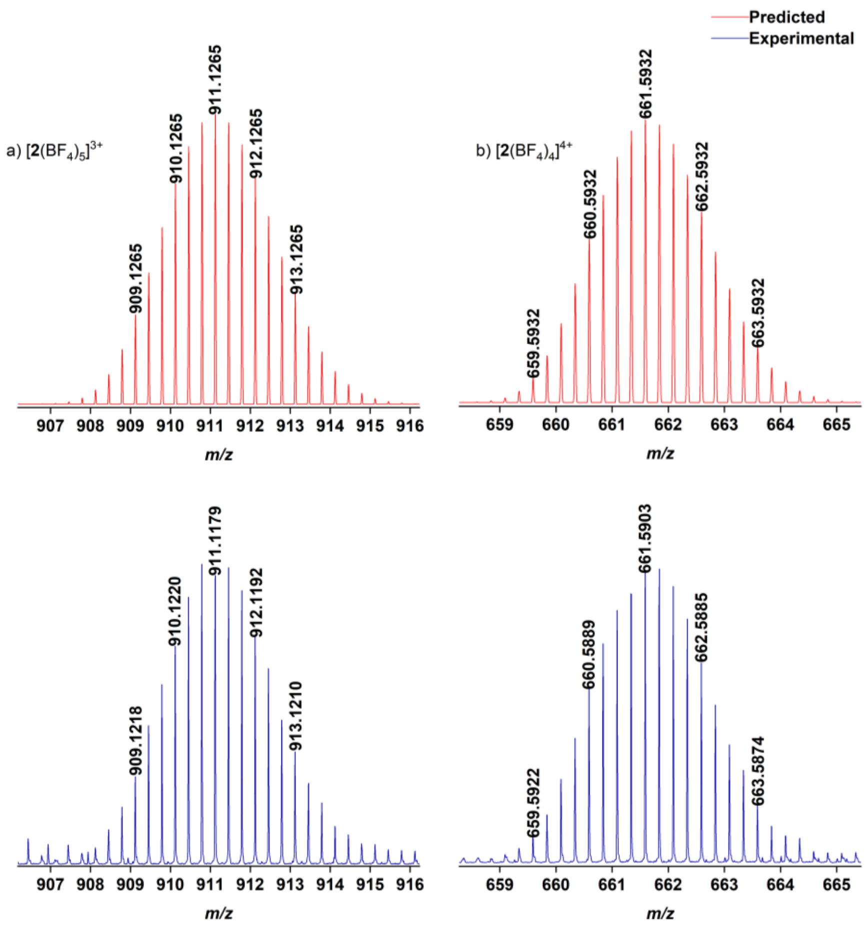

Figure S27: Isotopic distribution of selected peaks from tetrahedral cage **2** showing match between predicted and experimentally observed patterns (a) [**2**$(BF_4)_5]^{3+}$, (b) [**2**$(BF_4)_4]^{4+}$.

S5.3 Self-assembly of tetrahedral cage [4]$(BF_4)_8$ / $[Pd_4L^6{}_8](BF_4)_8$ and triangular cage [5]$(BF_4)_6$ / $[Pd_3L^6{}_6](BF_4)_6$

To further investigate whether the oxidation of cages **3''** and **3''T** via post-assembly modification led to a thermodynamic mixture of cages **4** and **5**, rather than a mixture containing any kinetically trapped products, ligand $\mathbf{L^6}$ was synthesised separately. Ligand $\mathbf{L^6}$ then underwent self-assembly with $[Pd(CH_3CN)_4](BF_4)_2$ over 18 h at 65 °C in CD_3CN to give the same mixture of tetrahedral cage **4** and triangular cage **5** as obtained via the post-assembly modification route (see Section S6.5), thus confirming a thermodynamic mixture of tetrahedral cage **4** and triangular cage **5** was being obtained via both routes.

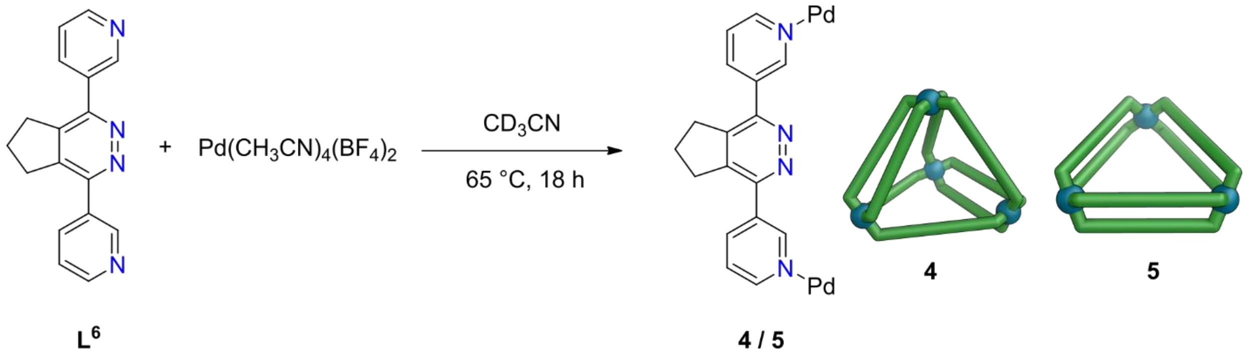

1,4-Di(pyridin-3-yl)-6,7-dihydro-5H-cyclopenta[d]pyridazine $\mathbf{L^6}$ (6.8 mg, 25 µmol) was dissolved in CD_3CN (0.6 mL). To this $[Pd(CH_3CN)_4](BF_4)_2$ (5.5 mg, 12.5 µmol) was added. The mixture was heated to 65 °C for 18 h, to afford a mixture of tetrahedral cage **4** and triangular cage **5** as a pale-yellow solution in a ratio of approximately 64.2% of cage **4** to 35.8% of cage **5**. This corresponds to 70.6% of ligand $\mathbf{L^6}$ being in cage **4** and 29.4% being in cage **5**.

The ratio of the two cages was determined through comparison of the integration of the signals from the 'a' protons in each cage. These protons were chosen as they were well separated from other signals. Cage **4** has a combined integral for protons **4**a + **4**a' of 16.26 with a total of 16 protons in both these environments in each cage, giving a relative proportion of cage **4** as $\frac{8.26+8}{16} = 1.01625$. Cage **5** has an integral for proton **5**a of 6.78, with a total of 12 protons in this environment in each cage, giving a relative proportion of cage **5** as $\frac{6.78}{12} = 0.565$ (**Figure S30**). Hence, the percentage of the two cages present can be calculated as $\frac{1.01625}{1.01625+0.565} \times 100\% = 64.2\%$ for cage **4** and $\frac{0.565}{1.01625+0.565} \times 100\% = 35.8\%$ for cage **5**.

The percentage of ligand in each cage can be determined by the proportion of the total integral (for protons **4**a, **4**a' and **5**a) that comes from either **4**a + **4**a', or from **5**a. The relative amount of ligand within environments **4**a/**4**a' is 16.26, and the relative amount of ligand within

environment **5**a is 6.78. Therefore, the percentage of ligand in cage **4** is $\frac{16.26}{6.78+8.00+8.26} \times 100\% = 70.6\%$ and the percentage of ligand in cage **5** is $\frac{6.78}{6.78+8.00+8.26} \times 100\% = 29.4\%$.

^{1}H NMR (500 MHz, CD_3CN) δ_H: 10.39 (s, 12H, **5**a), 10.22 (s, 8H, **4**a'), 9.45 (d, *J* = 5.7 Hz, 12H **5**b, 8H **4**b), 9.38 (s, 8H, **4**a), 9.11 (d, *J* = 5.6 Hz, 8H, **4**b'), 8.55 (dt, *J* = 8.2, 1.7 Hz, 8H, **4**d), 8.32 (ddt, *J* = 9.7, 8.1, 1.6 Hz, 12H **5**d, 8H **4**d'), 7.98 (dd, *J* = 8.1, 5.7 Hz, 8H **4**c), 7.84 (dd, *J* = 8.0, 5.7 Hz, 8H, **5**c), 7.71 (dd, *J* = 8.1, 5.7 Hz, 8H, **4**c'), 3.12 – 2.78 (m, 16H **4**f, 16H **4**f', 24H **5**f), 2.42 –2.37 (m, 8H **4**e, 8H **4**e', 12H **5**e). Note, the integrals stated correspond to one equivalent of each cage.

^{13}C NMR (126 MHz, CD_3CN) δ_C: 152.9, 152.3, 152.2, 151.4, 151.3, 151.2, 150.9, 150.8, 150.0, 145.4 (2 signals), 145.2, 140.8, 140.0, 139.8, 136.1, 136.0, 135.9, 128.2, 127.0 (2 signals), 32.2 (2 signals), 31.9, 24.8, 24.4. One aliphatic signal not observed (presumably due to overlap).

ESI-MS for [4]: (ESI, CD_3CN), *m/z*: $[\mathbf{4}(BF_4)_5]^{3+}$ calculated 1017.8766, found 1017.8648; $[\mathbf{4}(BF_4)_3]^{5+}$ calculated 576.1234, found 576.1157; $[\mathbf{4}(BF_4)]^{7+}$ calculated 386.6577, found 386.6546

ESI-MS for [5]: (ESI, CD_3CN), *m/z*: $[\mathbf{5}(BF_4)_2]^{4+}$ calculated 534.6152, found 534.6103; $[\mathbf{5}(BF_4)]^{5+}$ calculated 410.2909, found 410.2878; $[\mathbf{5}]^{6+}$ calculated 327.4080, found 327.4055.

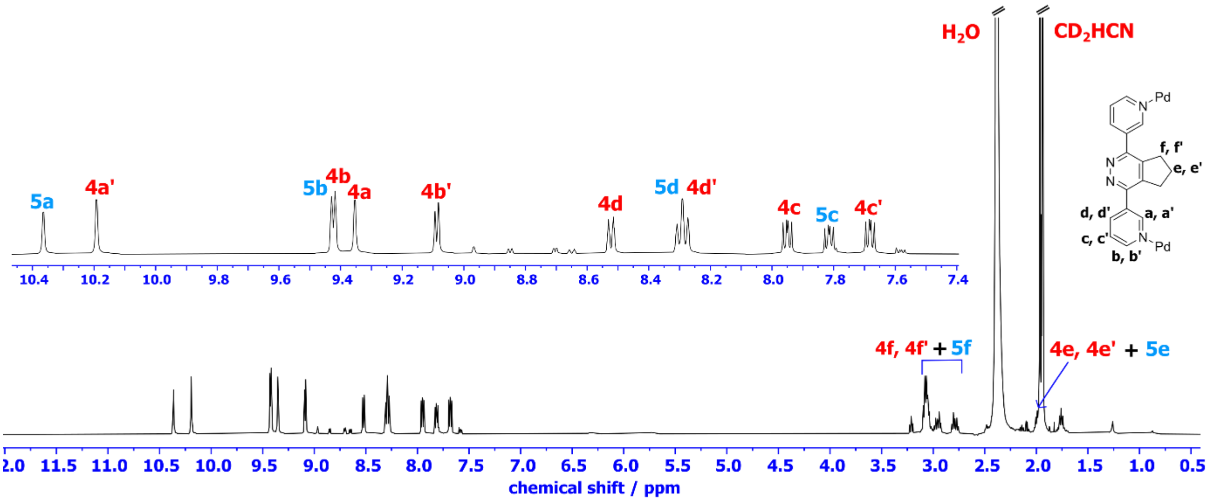

Figure S28: ^{1}H NMR spectrum of tetrahedral cage **4** and triangular cage **5** (500 MHz, 298 K, CD_3CN)

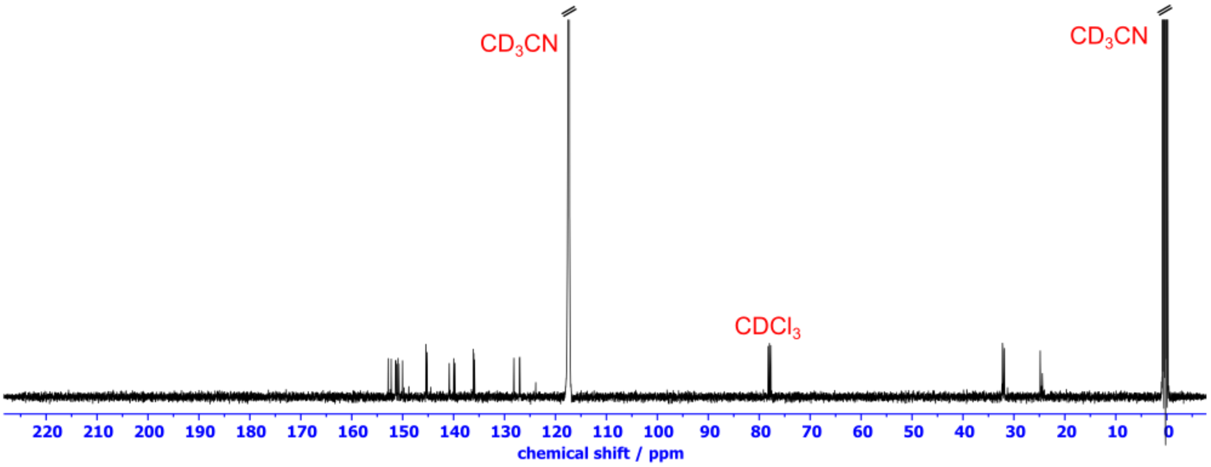

Figure S29: ^{13}C NMR spectrum of tetrahedral cage **4** and triangular cage **5** (126 MHz, 298 K, CD_3CN)

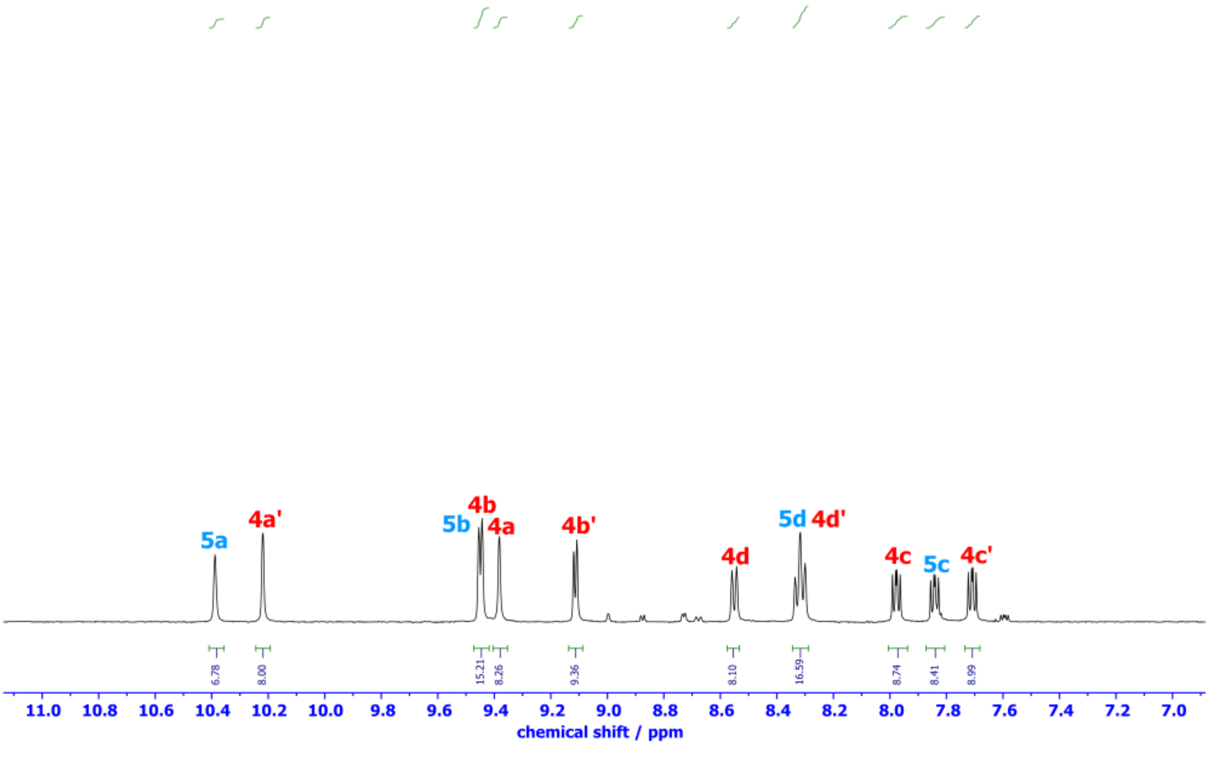

Figure S30: Integration of the aromatic region of the ^{1}H NMR spectrum of tetrahedral cage **4** and triangular cage **5**
(400 MHz, 298 K, CD_3CN)

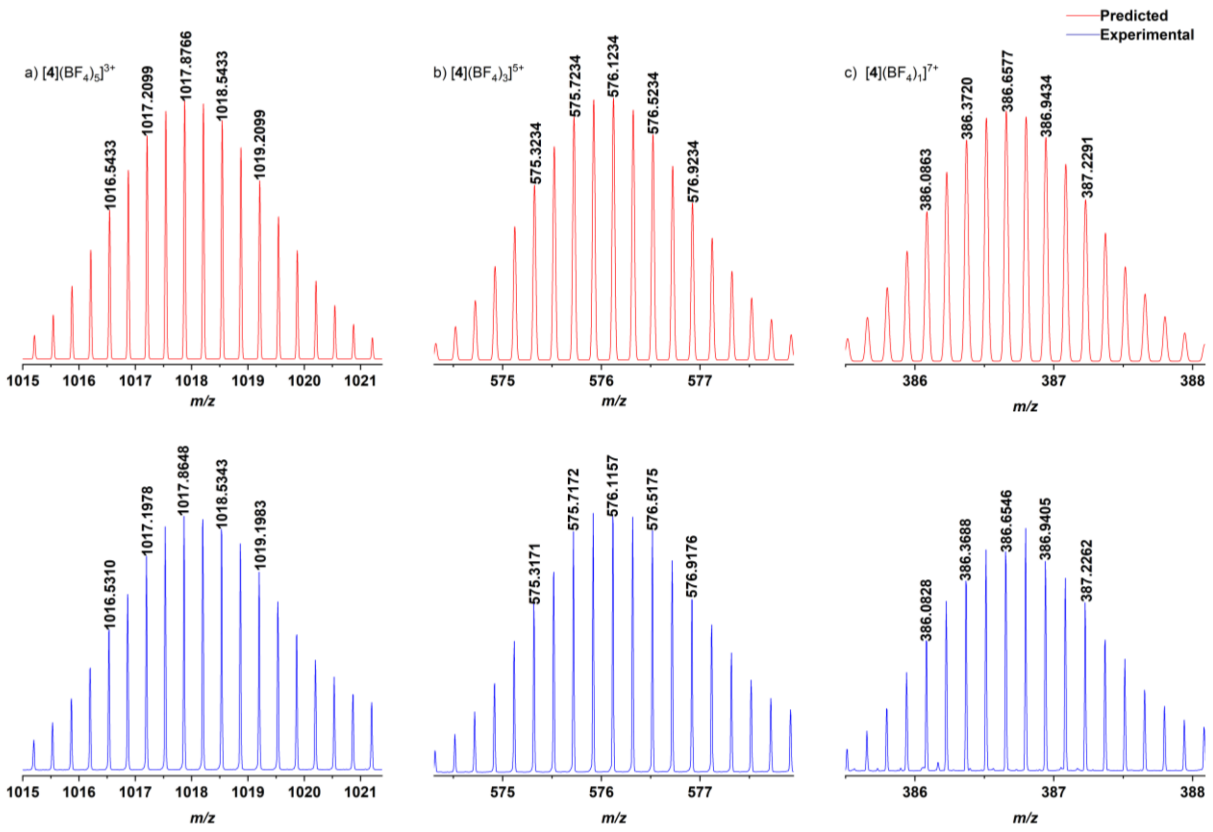

Figure S31: Isotopic distribution of selected peaks from tetrahedral cage **4** showing match between predicted and experimentally observed patterns (a) [**4**$(BF_4)_5]^{3+}$, (b) [**4**$(BF_4)_3]^{5+}$, (c) [**4**$(BF_4)]^{7+}$

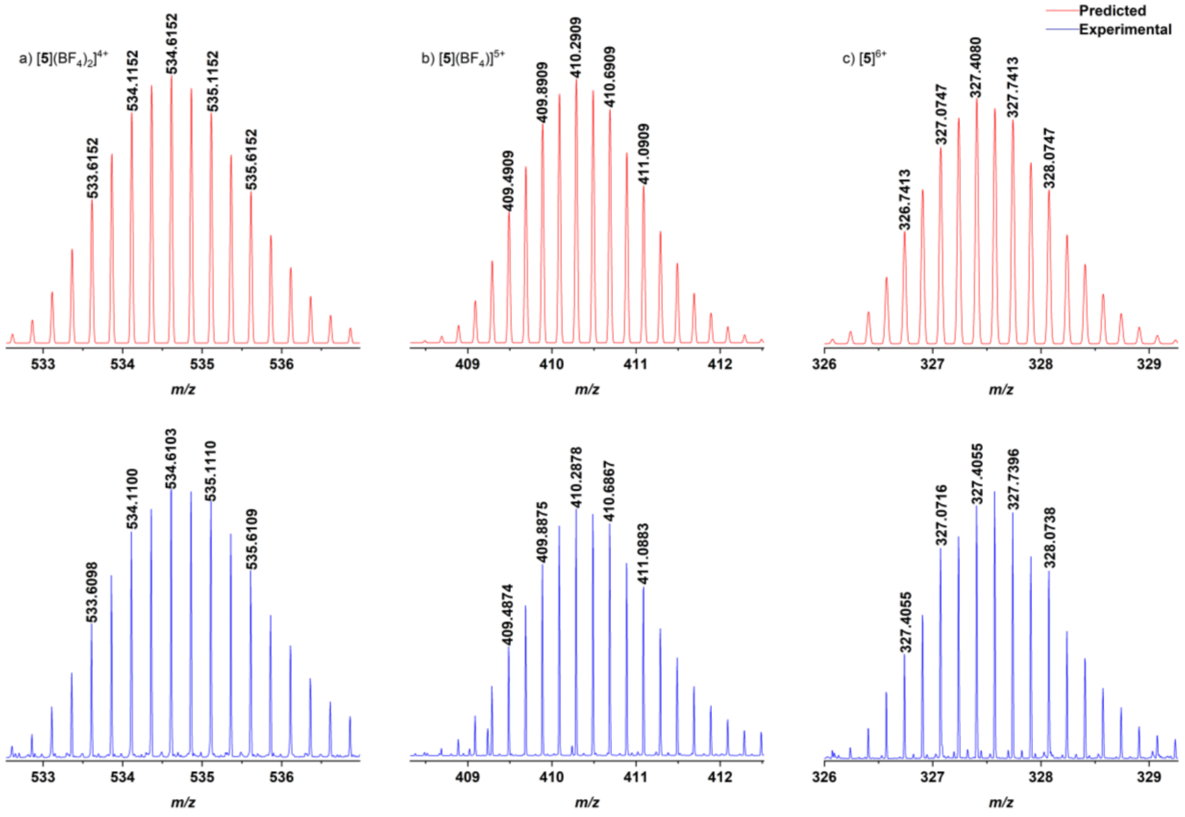

Figure S32: Isotopic distribution of selected peaks from triangular cage **5** showing match between predicted and experimentally observed patterns.
(a) [**5**(BF_4)$_2$]$^{4+}$, (b) [**5**(BF_4)]$^{5+}$, (c) [**5**]$^{6+}$

S6 Post-Assembly Modification of Cages

S6.1 Post-assembly modification route to tetrahedral cage [2]$(BF_4)_8$ / $[Pd_4L^2_8](BF_4)_8$

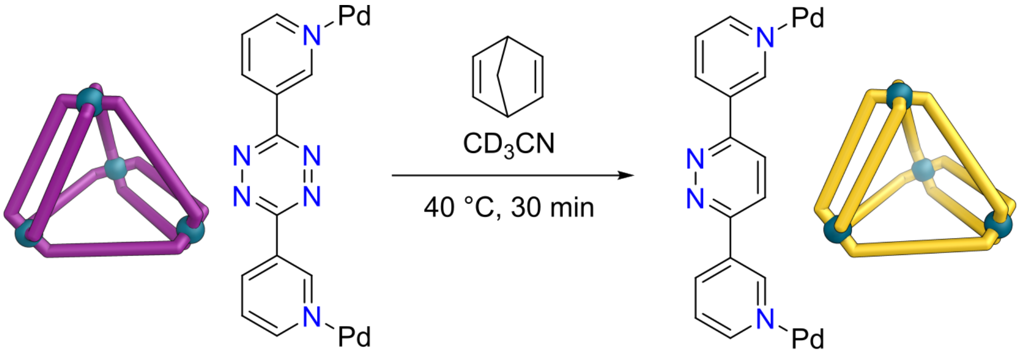

1 **2**

Tetrazine cage **1** (4.72 mg, 1.57 µmol) was dissolved in CD_3CN (0.6 mL). Norbornadiene (6.08 mg, 66.2 µmol) was added to the solution which was stirred at 40 ˚C for 30 min to give a pale-yellow solution of pyridazine cage **2**.

^1H NMR and ESI ESI-MS data matched that of the sample prepared from self-assembly of ligand $\mathbf{L^2}$ with $[Pd(CH_3CN)_4](BF_4)_2$ (see Section S5.2).

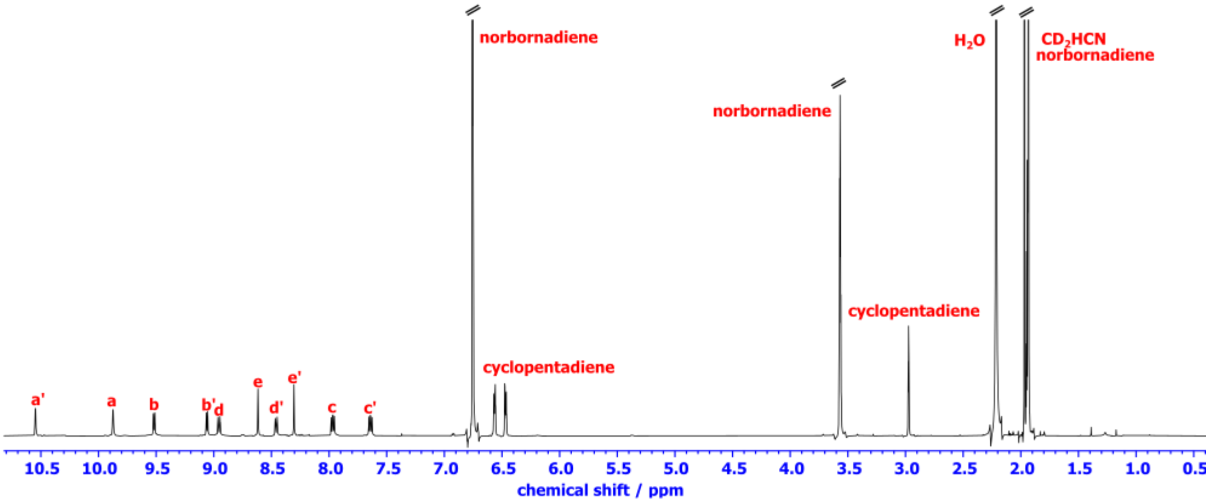

Figure S33: ^1H NMR spectrum of tetrahedral cage **2** produced by post-assembly modification
(400 MHz, 298 K, CD_3CN)

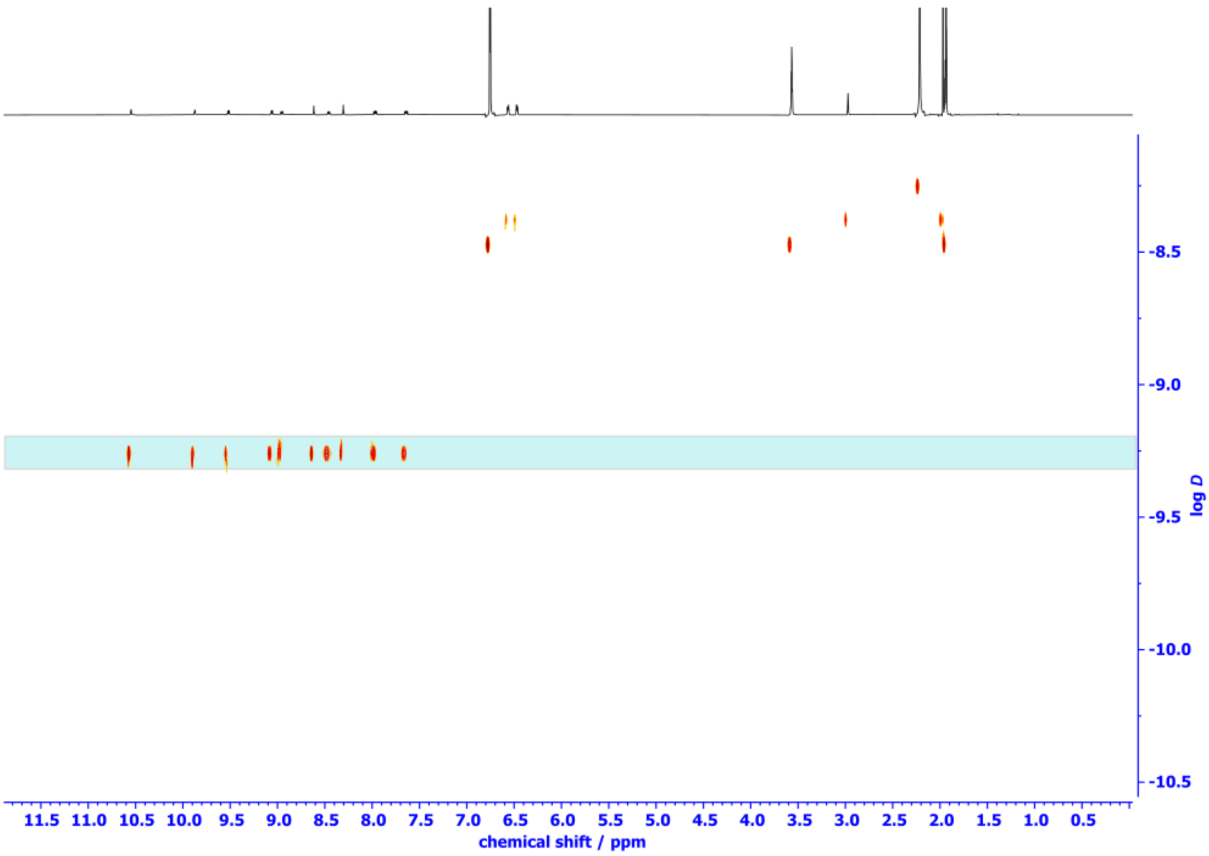

Figure S34: ^{1}H DOSY spectrum of tetrahedral cage **2**
(500 MHz, 298 K, CD_3CN)

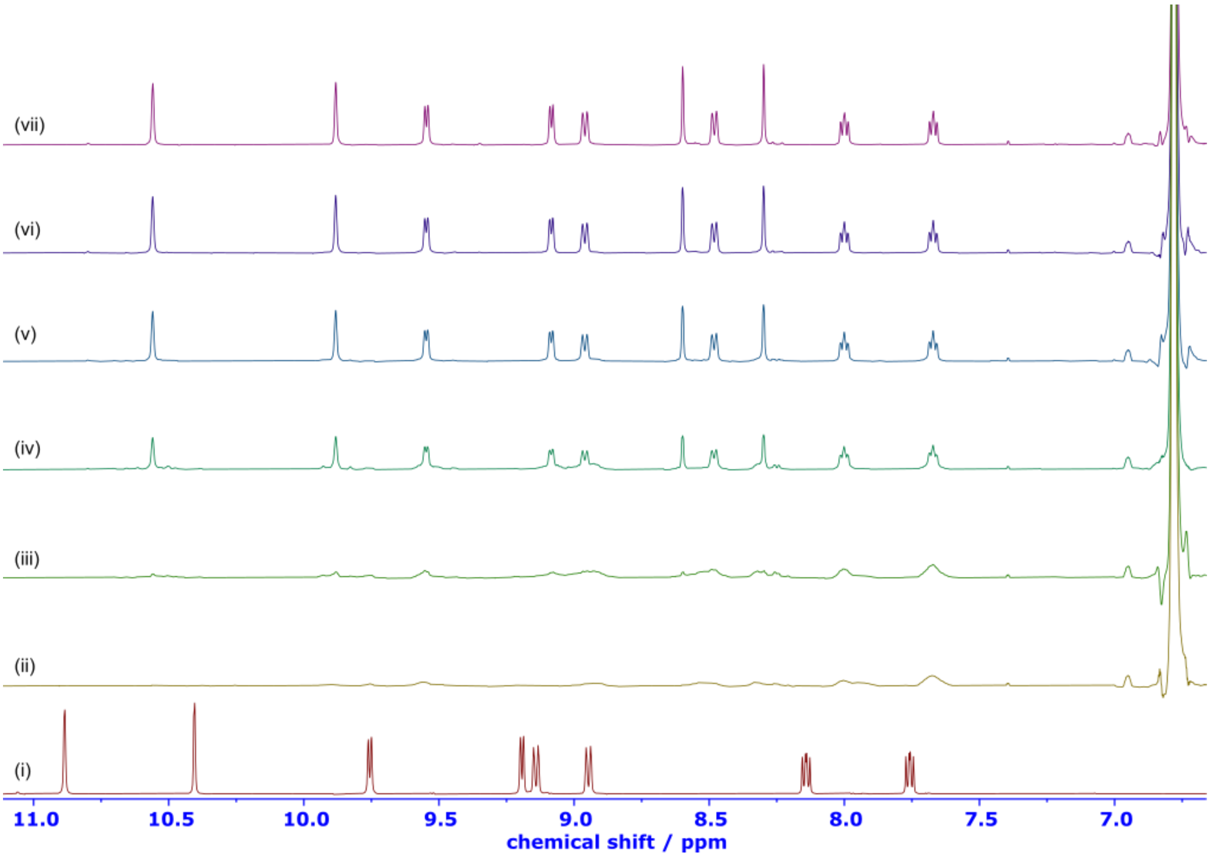

Figure S35: Stacked plot of ^1H NMR spectra of transformation of tetrahedral cage **1** to tetrahedral cage **2** at 313 K. (i) Tetrahedral cage **1** before addition of norbornadiene (NBD); (ii) 2 min after NBD addition; (iii) 5 min after NBD addition; (iv) 10 min after NBD addition; (v) 15 min after NBD addition; (vi) 20 min after NBD addition; (vii) 30 min after NBD addition. (500 MHz, 313 K, CD_3CN)

S6.2 Post-assembly modification route to lantern cage [3](BF_4)$_4$ / [$Pd_2L^3_4$](BF_4)$_4$ and tetrahedral cage [3T](BF_4)$_8$ / [$Pd_4L^3_8$](BF_4)$_8$

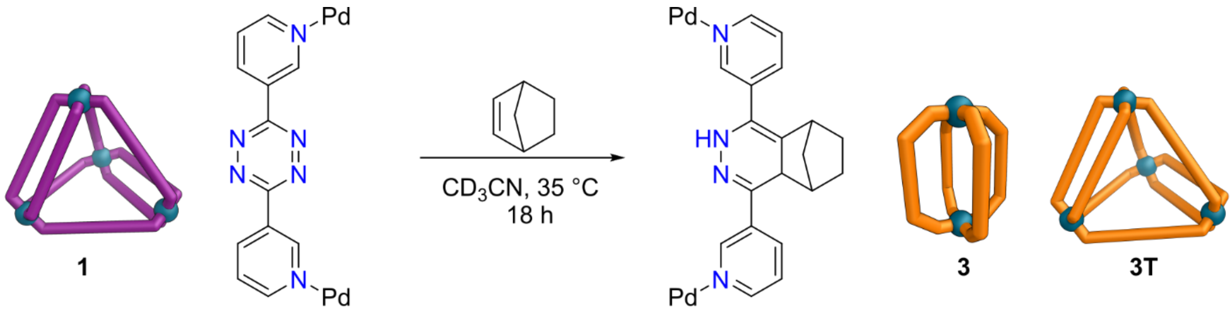

1 **3 / 3T**

Tetrazine cage **1** (4.7 mg, 1.57 µmol) was dissolved in CD_3CN (0.6 mL). To this norbornene (2.9 mg, 30.9 µmol) was added and the solution stirred at 35 ˚C for 18 h to produce a pale brown solution of **3** and **3T**.

^{1}H NMR (500 MHz, CD_3CN) Due to the complex nature of the spectrum no assignments were made.

^{13}C NMR (126 MHz, CD_3CN) Due to the complex nature of the ^{1}H NMR spectrum no ^{13}C NMR spectrum was attempted.

ESI-MS for [3]: (ESI, CD_3CN), *m/z*: [**3**(BF_4)$_3$]$^+$ calculated 1682.4434, found 1682.4349; [**3**(BF_4)$_2$]$^{2+}$ calculated 798.2184, found 798.2170; [**3**(BF_4)]$^{3+}$ calculated 503.1434, found 503.1420.

ESI-MS for [3T] (ESI, CD_3CN), *m/z*: [**3T**(BF_4)$_6$]$^{2+}$ calculated 1682.4434, found 1682.4349; [**3T**(BF_4)$_5$]$^{3+}$ calculated 1092.6267, found 1092.6191; [**3T**(BF_4)$_3$]$^{5+}$ calculated 620.9734, found 620.9683.

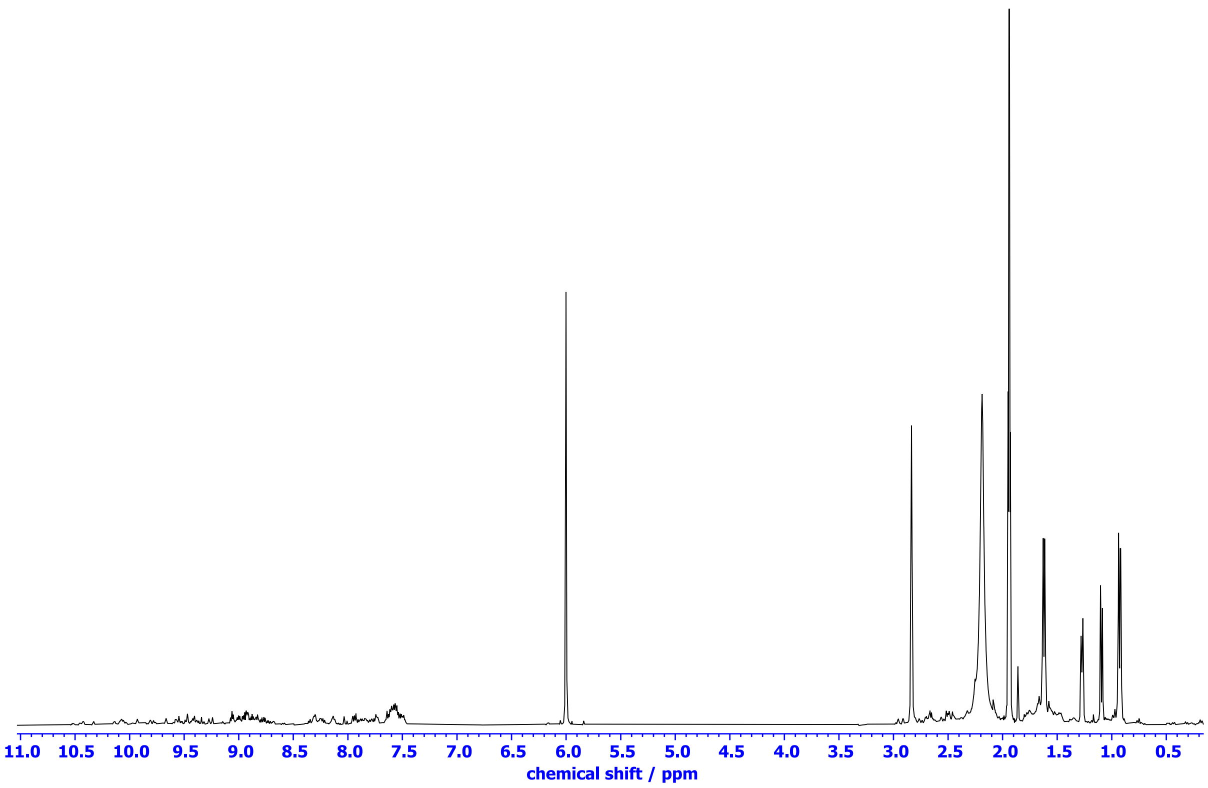

Figure S36: ^{1}H NMR spectrum of lantern cage **3** and tetrahedral cage **3T** (400 MHz, 298 K, CD_3CN)

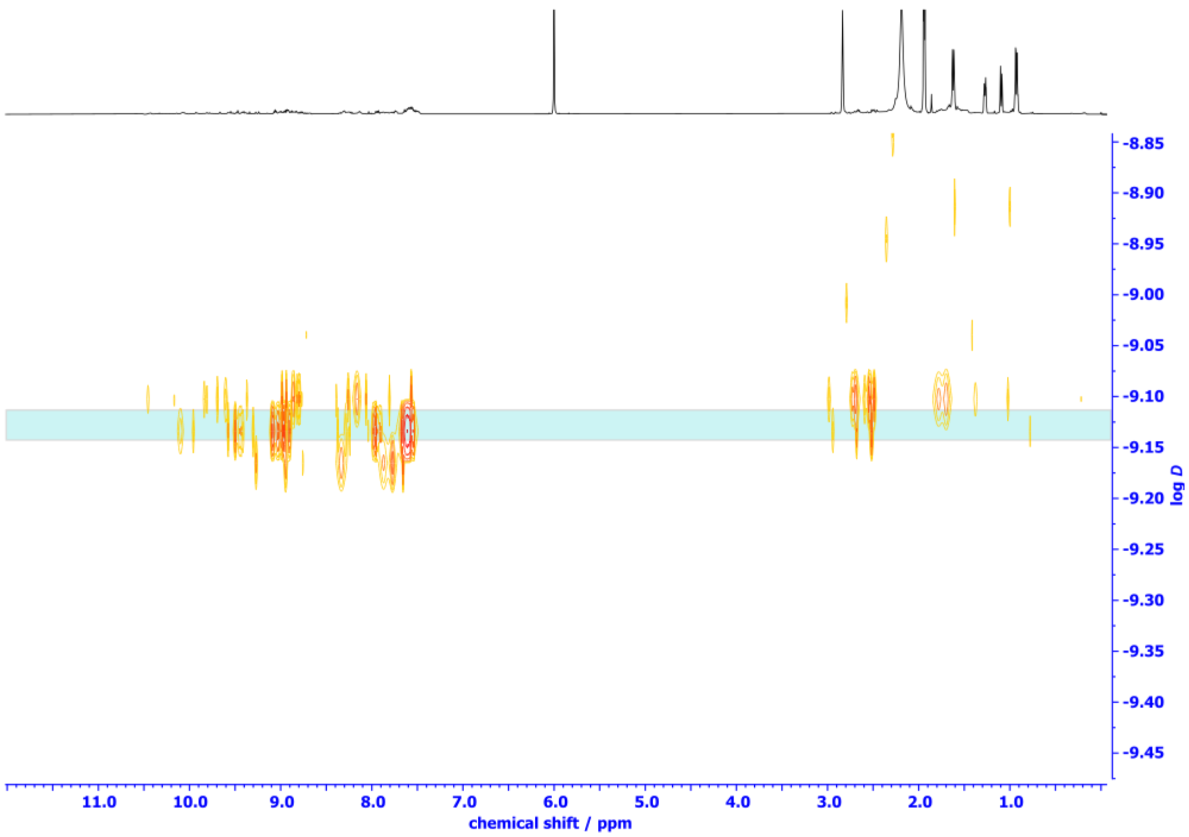

Figure S37: ^{1}H DOSY spectrum of lantern cage **3** and tetrahedral cage **3T** (500 MHz, 298 K, CD_3CN)

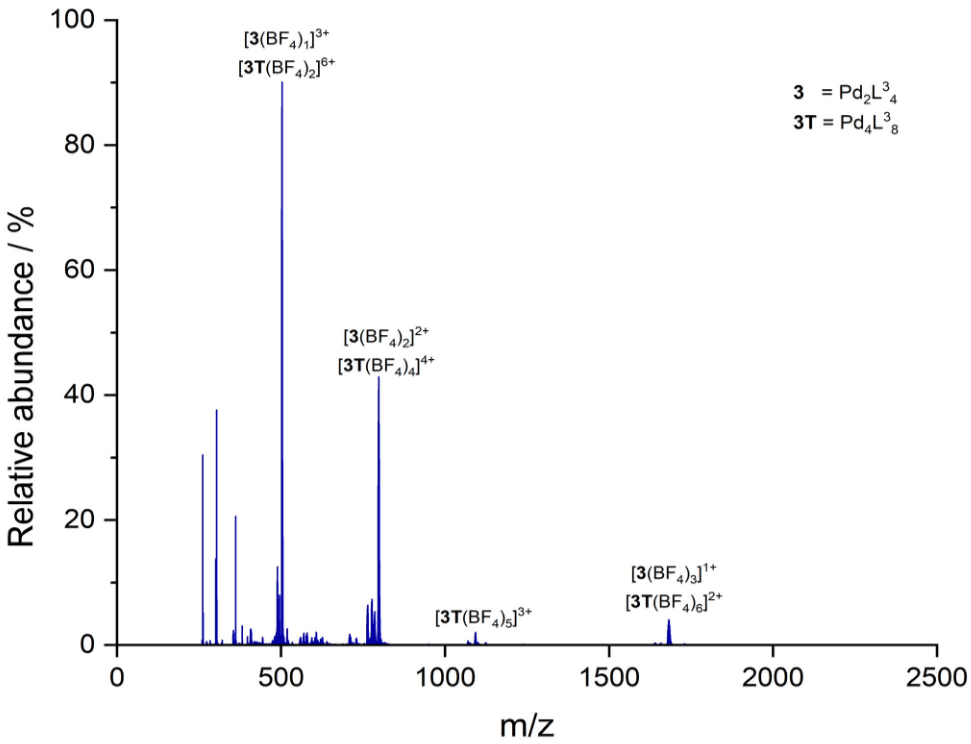

Figure S38: ESI-MS of lantern cage **3** and tetrahedral cage **3T**

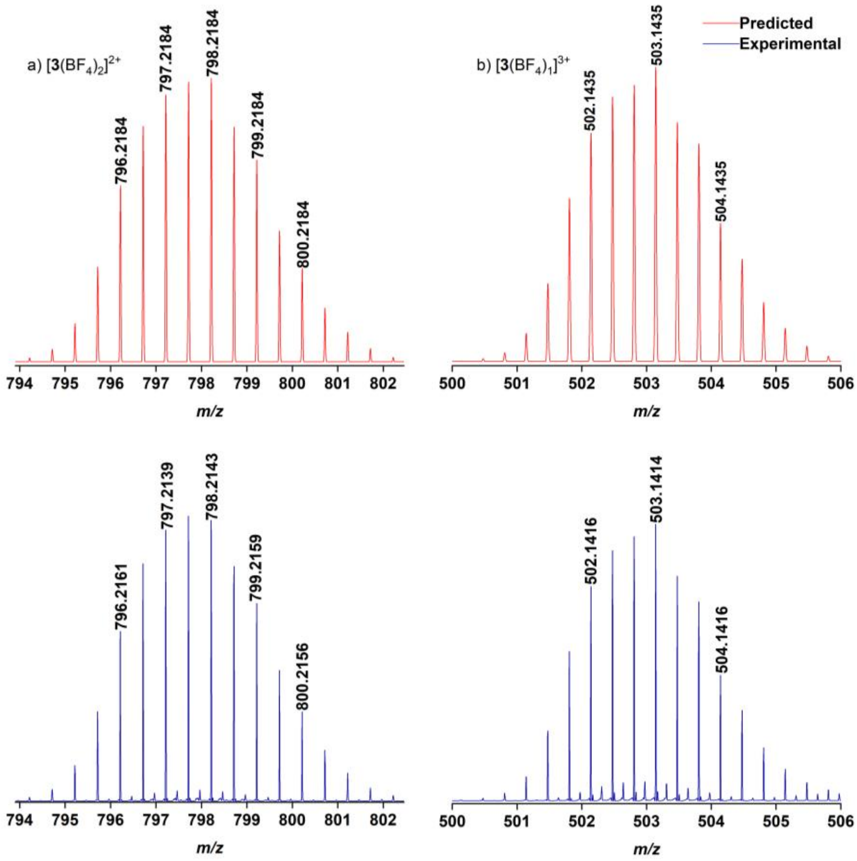

Figure S39: Isotopic distribution of selected peaks from lantern cage **3** showing match between predicted and experimentally observed patterns

(a) [**3**(BF_4)$_2$]$^{2+}$, (b) [**3**(BF_4)]$^{3+}$

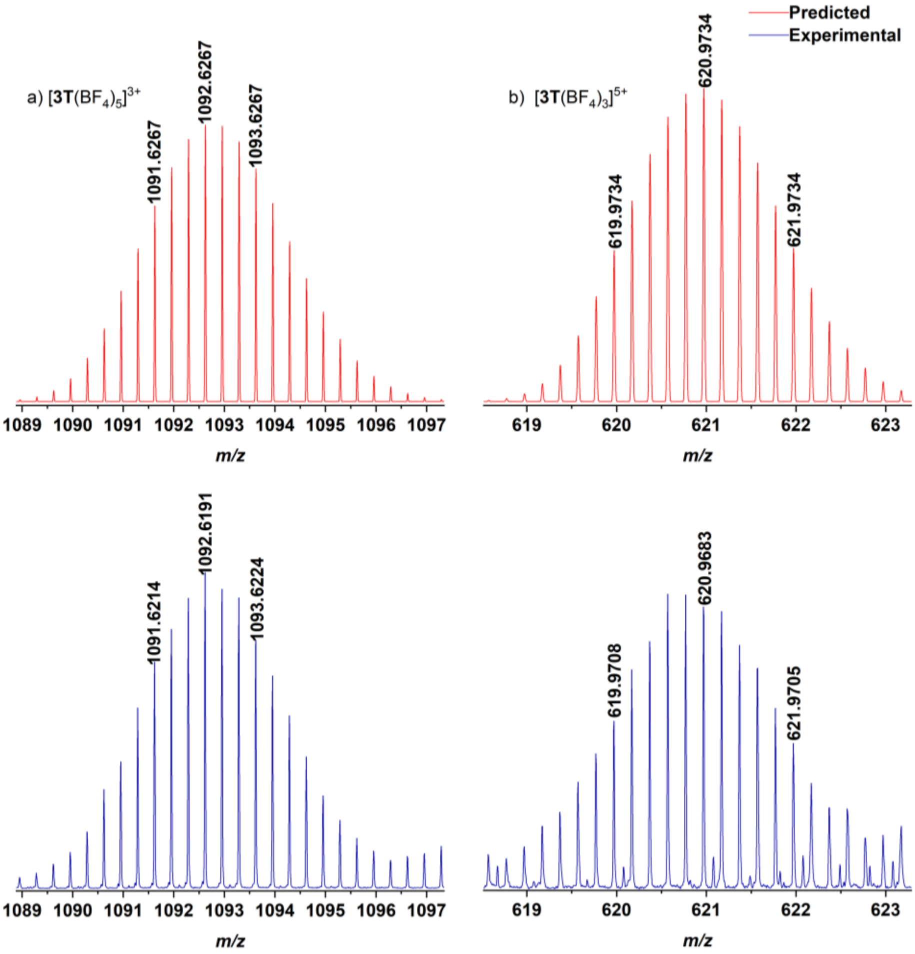

Figure S40: Isotopic distribution of selected peaks from tetrahedral cage **3T** showing match between predicted and experimentally observed patterns.
(a) [**3T**$(BF_4)_5$]$^{3+}$, (b) [**3T**$(BF_4)_3$]$^{5+}$

S6.3 Post-assembly modification route to lantern cage $[3'](BF_4)_4$ / $[Pd_2L^4{}_4](BF_4)_4$ and tetrahedral cage $[3'T](BF_4)_8$ / $[Pd_4L^4{}_8](BF_4)_8$

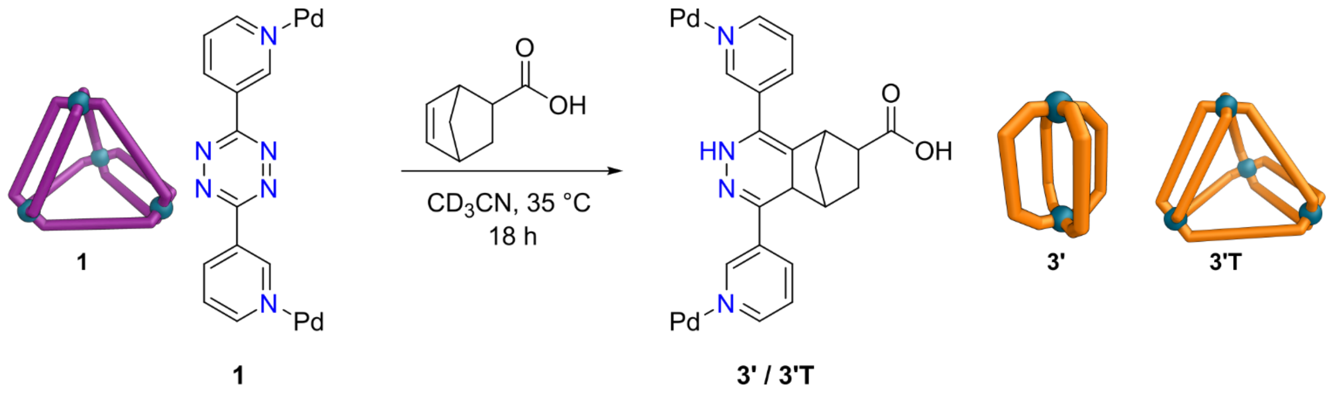

Tetrazine cage **1** (4.7 mg, 1.57 µmol) was dissolved in CD_3CN (0.6 mL) and added to bicycle[2.2.1]hept-5-ene-2-carboxylic acid (7.2 mg, 52.1 µmol) and the solution stirred at 35 ˚C for 18 h to produce a pale brown solution of **3'** and **3'T**.

^{1}H NMR (500 MHz, CD_3CN) Due to the complex nature of the spectrum no assignments were made.

^{13}C NMR (126 MHz, CD_3CN) Due to the complex nature of the ^{1}H NMR spectrum no ^{13}C NMR spectrum was attempted.

ESI-MS for [3']: (ESI, CD_3CN), *m/z*: [**3'**$(BF_4)_2]^{2+}$ calculated 886.1980, found 886.1957; [**3'**$(BF_4)]^{3+}$ calculated 561.7966, found 561.7965; [**3'**]$^{4+}$ calculated 399.5958, found 399.5954.

ESI-MS for [3'T]: (ESI, CD_3CN), *m/z*: [**3'T**$(BF_4)_5]^{3+}$ calculated 1210.2663, found 1210.2432; [**3'T**$(BF_4)_3]^{5+}$ calculated 691.3572, found 691.3556.

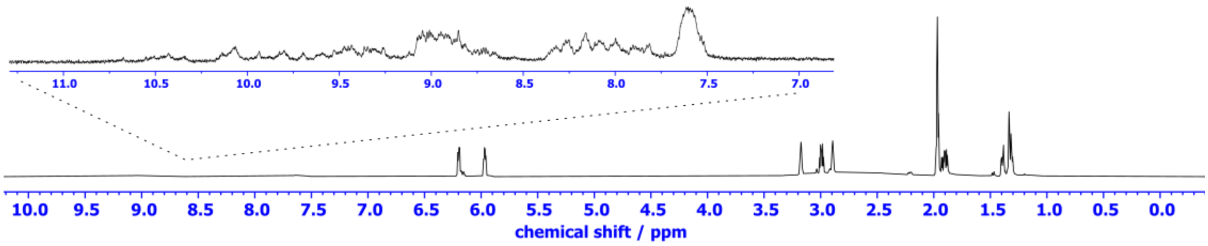

Figure S41: ^{1}H NMR spectrum of lantern cage **3'** and tetrahedral cage **3'T** (500 MHz, 298 K, CD_3CN)

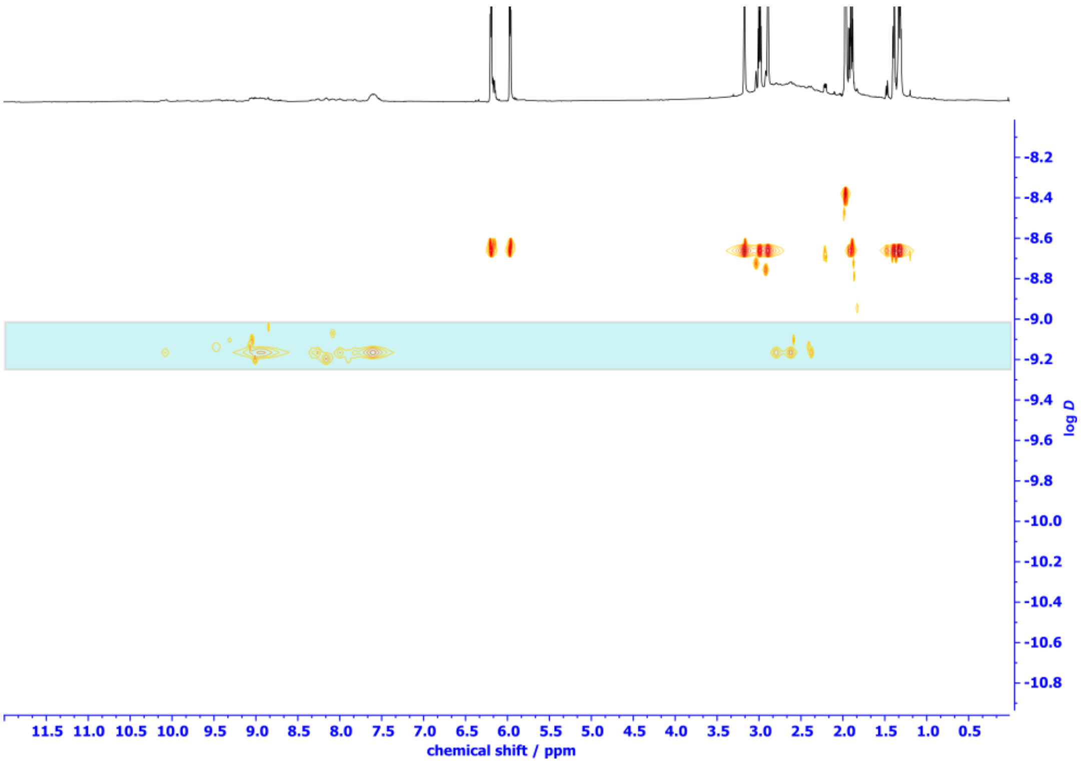

Figure S42: ^{1}H DOSY spectrum of lantern cage **3’** and tetrahedral cage **3’T** (500 MHz, 298 K, CD_3CN)

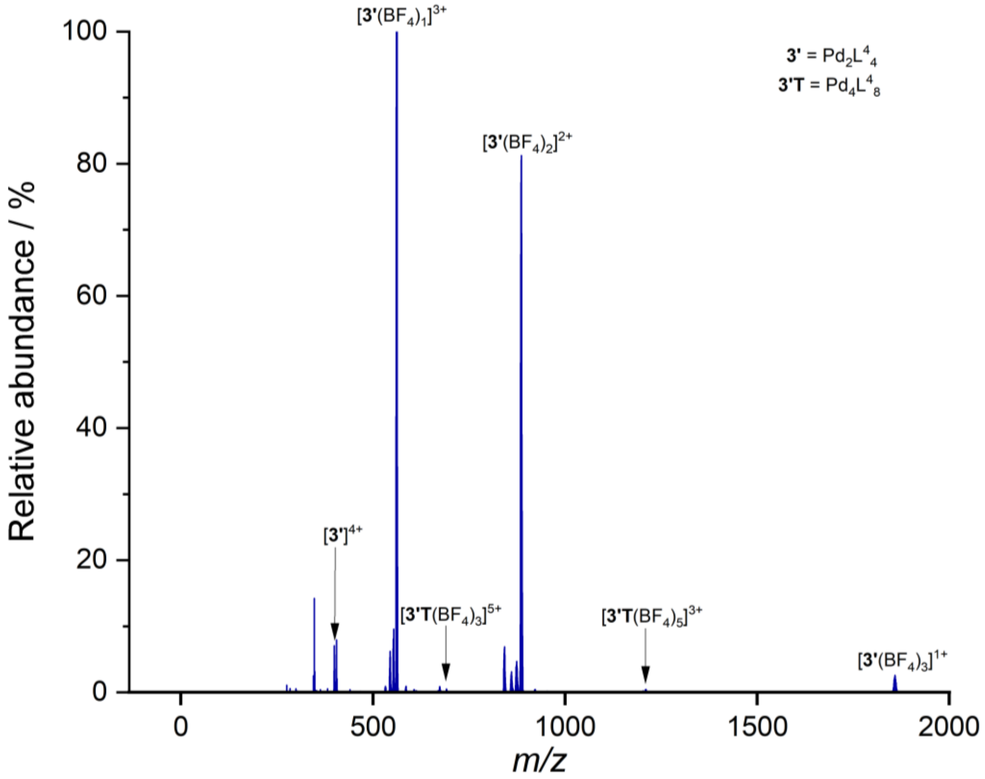

Figure S43: ESI-MS of lantern cage **3’** and tetrahedral cage **3’T**

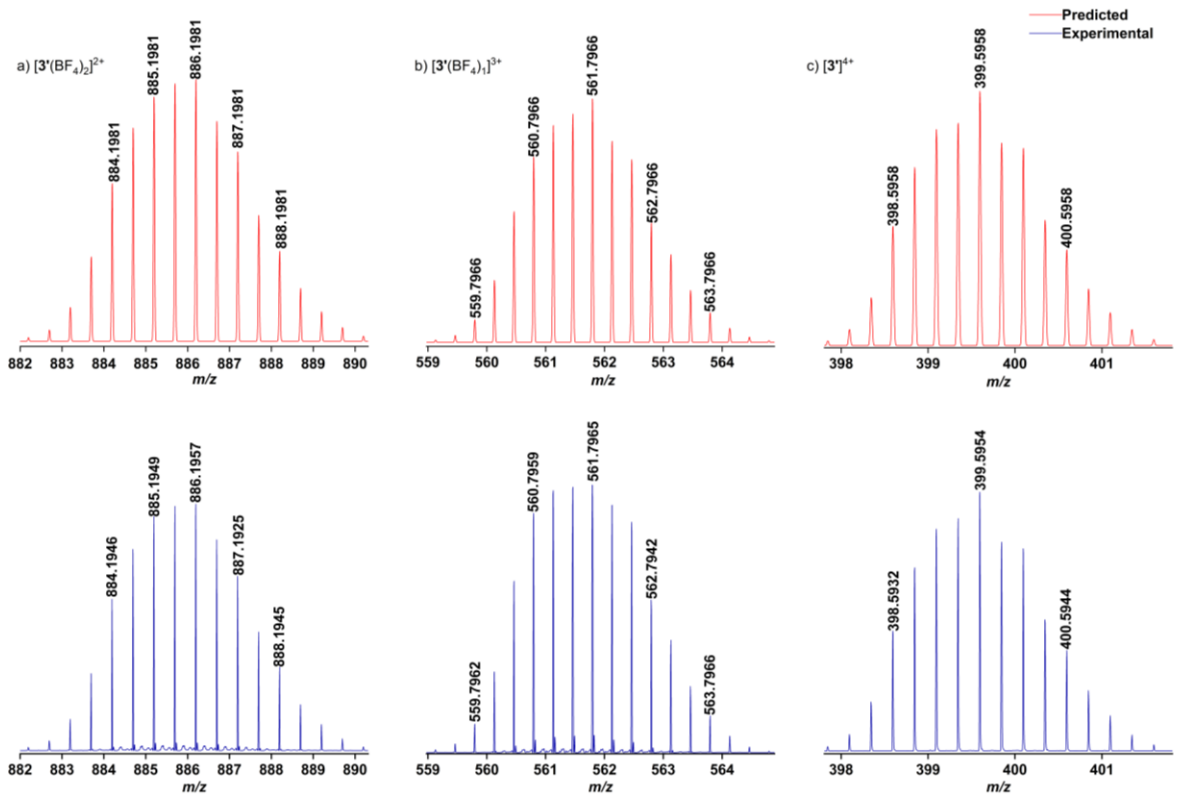

Figure S44: Isotopic distribution of selected peaks from lantern cage **3’** showing match between predicted and experimentally observed patterns
(a) [**3’**$(BF_4)_2]^{2+}$, (b) [**3’**$(BF_4)]^{3+}$, (c) [**3’**$]^{4+}$

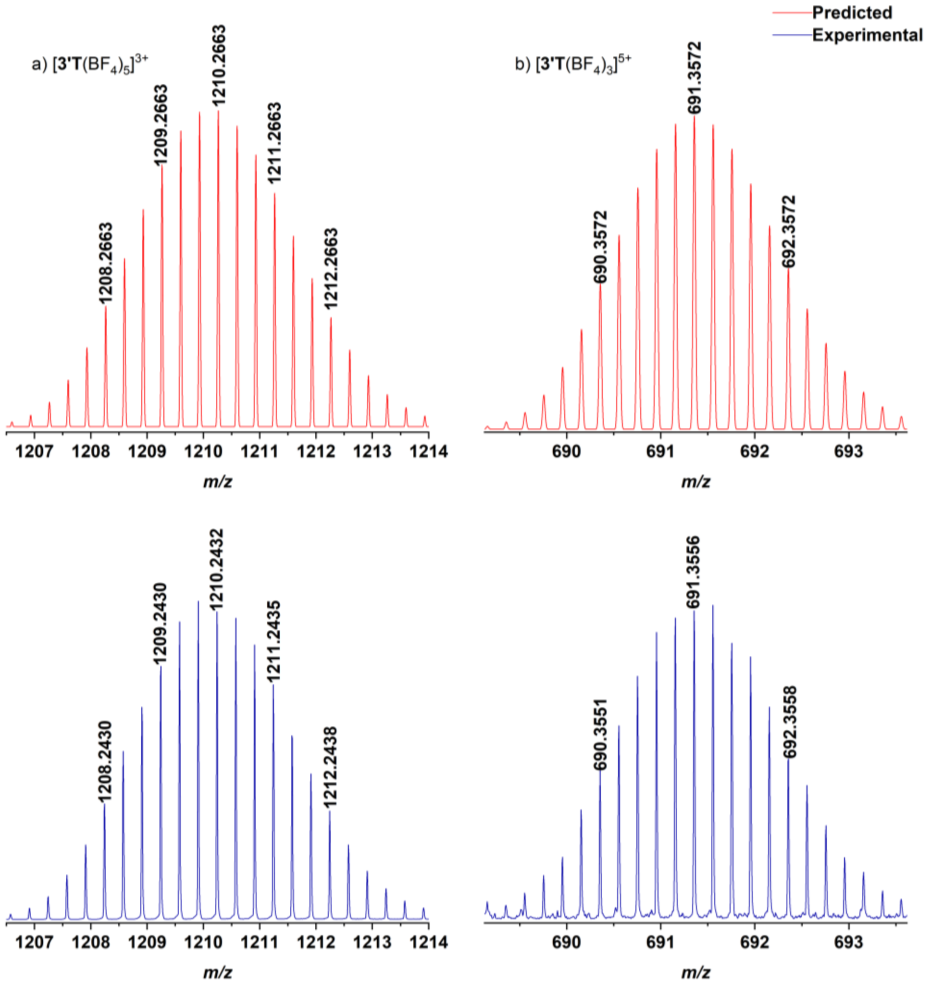

Figure S45: Isotopic distribution of selected peaks from tetrahedral cage **3'T** showing match between predicted and experimentally observed patterns
(a) [**3'T**(BF_4)$_5$]$^{3+}$, (b) [**3'T**(BF_4)$_3$]$^{5+}$

S6.4 Post-assembly modification route to lantern cage [3''](BF$_4$)$_4$ / [Pd$_2$L^5$_4$](BF$_4$)$_4$ and tetrahedral cage [3''T](BF$_4$)$_8$ / [Pd$_4$L^5$_8$](BF$_4$)$_8$

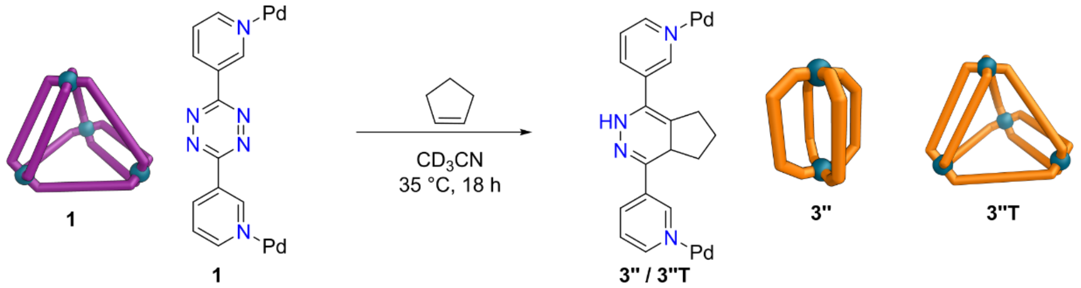

Tetrazine cage **1** (4.7 mg, 1.57 µmol) was dissolved in CD_3CN (0.6 mL) and added to cyclopentene (2.6 mg, 3.3 µL, 37.5 µmol) and the solution stirred at 35 ˚C for 18 h to produce a pale-yellow solution of **3''** and **3''T**.

^{1}H NMR (500 MHz, CD_3CN) Due to the complex nature of the spectrum no assignments were made. Upon cooling the solution down and running a ^{1}H NMR spectrum at a lower temperature of 245 K no significant changes / sharpening was seen in the spectrum, indicating the complexity was likely due to mixtures of structures and mixtures of isomers of each structure, rather than fluxional processes.

^{13}C NMR (126 MHz, CD_3CN) Due to the complex nature of the ^{1}H NMR spectrum no ^{13}C NMR spectrum was attempted.

ESI-MS for [3'']: (ESI, CD_3CN), *m/z*: $[Pd_2L^5_4(BF_4)_2]^{2+}$ calculated 746.1871, found 746.1794; $[Pd_2L^5_4(BF_4)]^{3+}$ calculated 468.4559, found 468.4527.

ESI-MS for [3''T]: (ESI, CD_3CN), *m/z*: $[Pd_4L^5_8(BF_4)_5]^{3+}$ calculated 1023.2517, found 1023.2316; $[Pd_4L^5_8(BF_4)_4]^{4+}$ calculated 745.6871, found 745.6803; $[Pd_4L^5_8(BF_4)_3]^{5+}$ calculated 579.3484, found 579.3377; $[Pd_4L^5_8(BF_4)_2]^{6+}$ calculated 468.2893, found 468.2866.

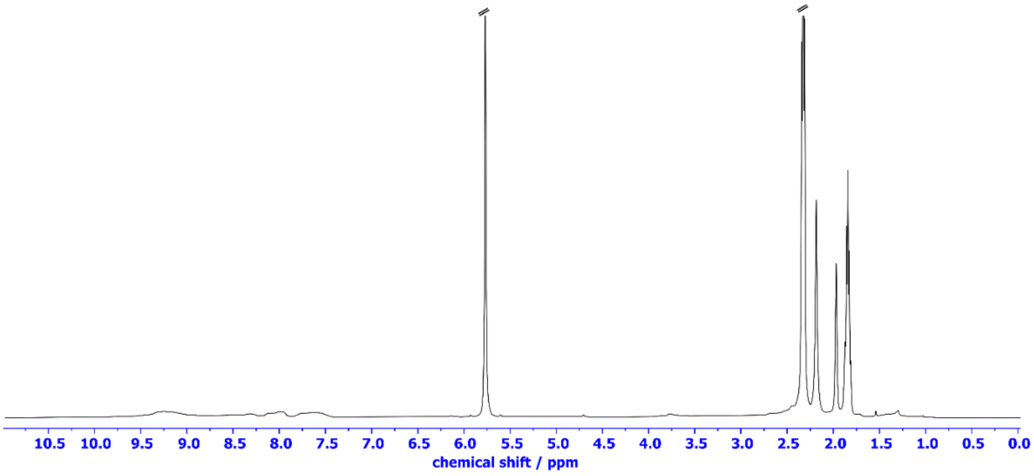

Figure S46: ^{1}H NMR spectrum of lantern cage **3''** and tetrahedral cage **3''T** (500 MHz, 298 K, CD_3CN)

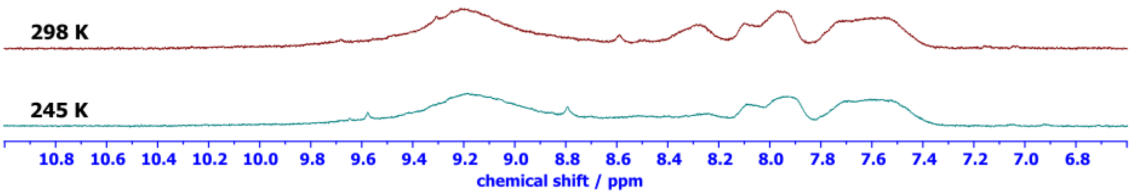

Figure S47: Low temperature ^{1}H NMR spectra of the mixture of **3''** and **3''T** (spectra at 245 K compared to 298 K)

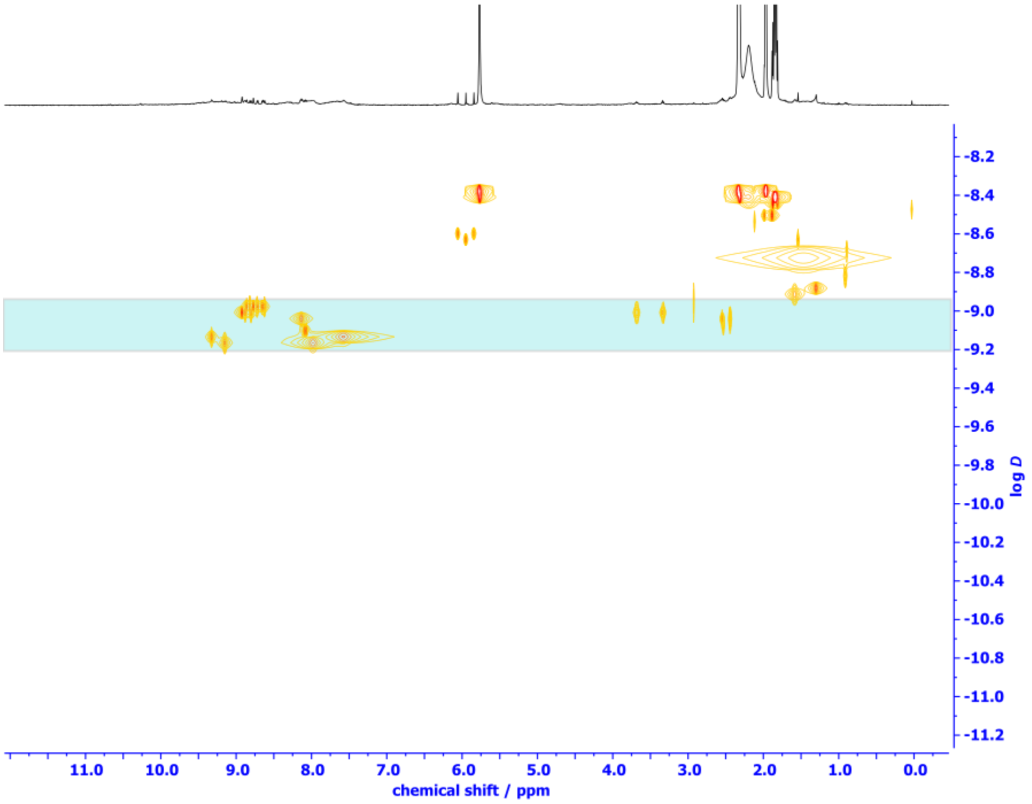

Figure S48: ^{1}H DOSY spectrum of lantern cage **3''** and tetrahedral cage **3''T** (500 MHz, 298 K, CD_3CN)

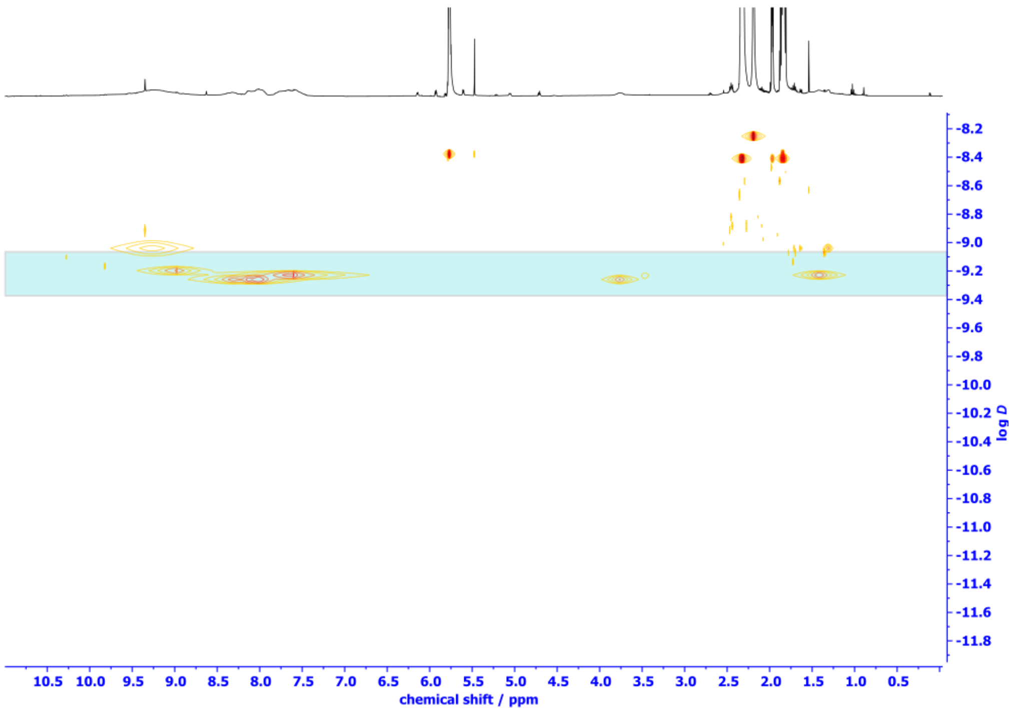

Figure S49: ^{1}H DOSY spectrum of tetrahedral cage **3''T** immediately after synthesis under inert conditions
(500 MHz, 298 K, CD_3CN)

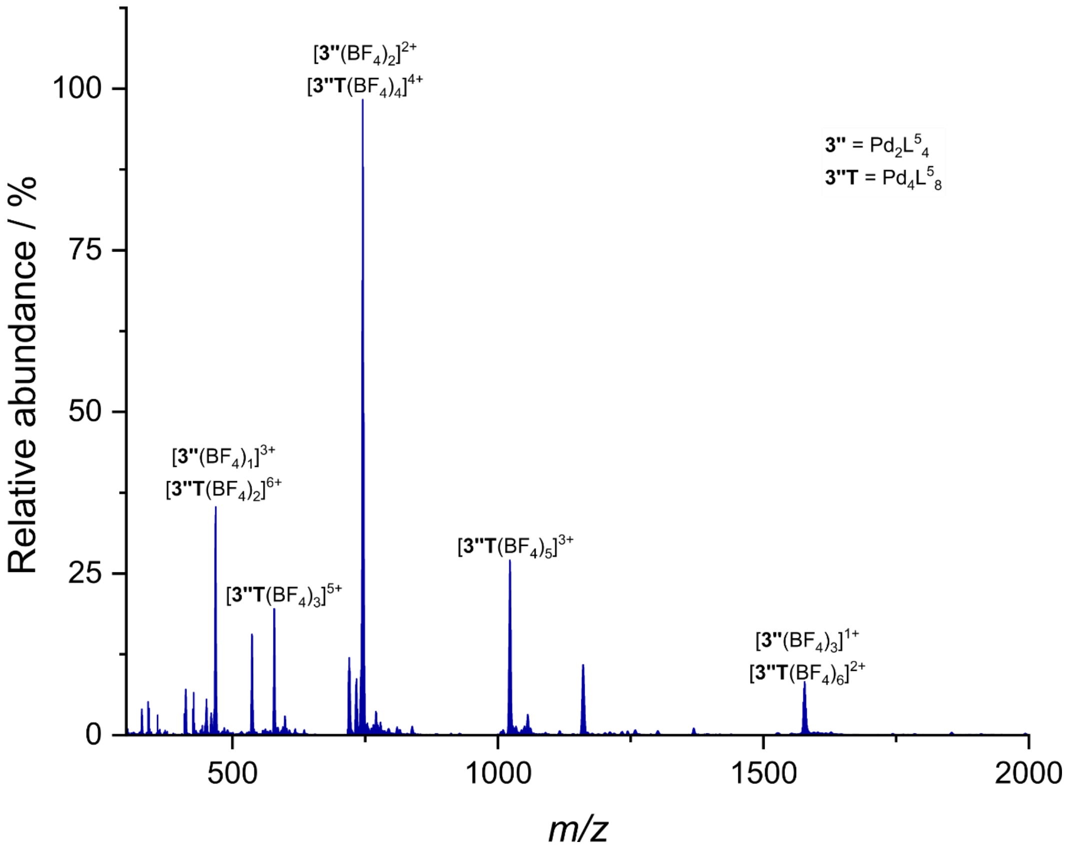

Figure S50: ESI-MS of lantern cage **3''** and tetrahedral cage **3''T**

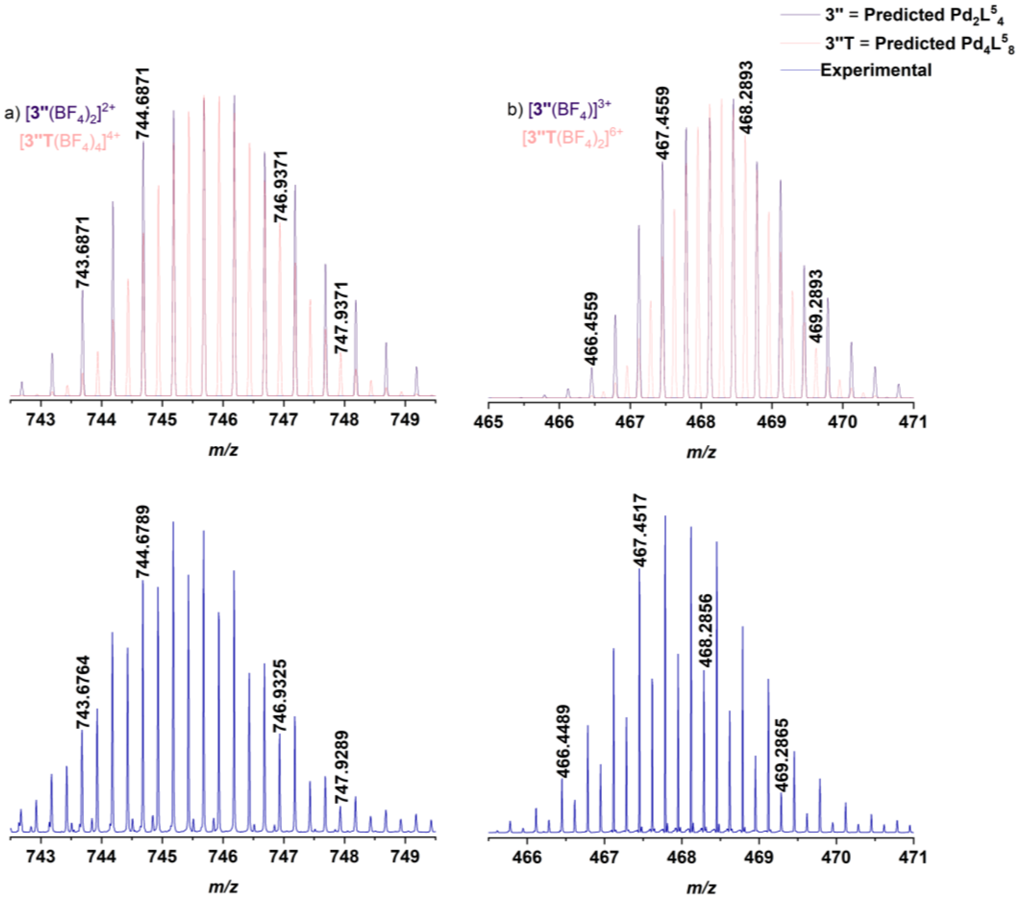

Figure S51: Isotopic distribution of selected peaks from lantern cage **3’’** overlaid with selected peaks from tetrahedral cage **3’’T** showing match between predicted and experimentally observed patterns.
(a) [**3’’**$(BF_4)_2$]$^{2+}$ overlaid with [**3’’T**$(BF_4)_4$]$^{4+}$, (b) [**3’’**(BF_4)]$^{3+}$ overlaid with [**3’’T**$(BF_4)_2$]$^{6+}$. No lone signals for [**3’’**] species were recorded without overlapping signals from [**3’’T**] species.

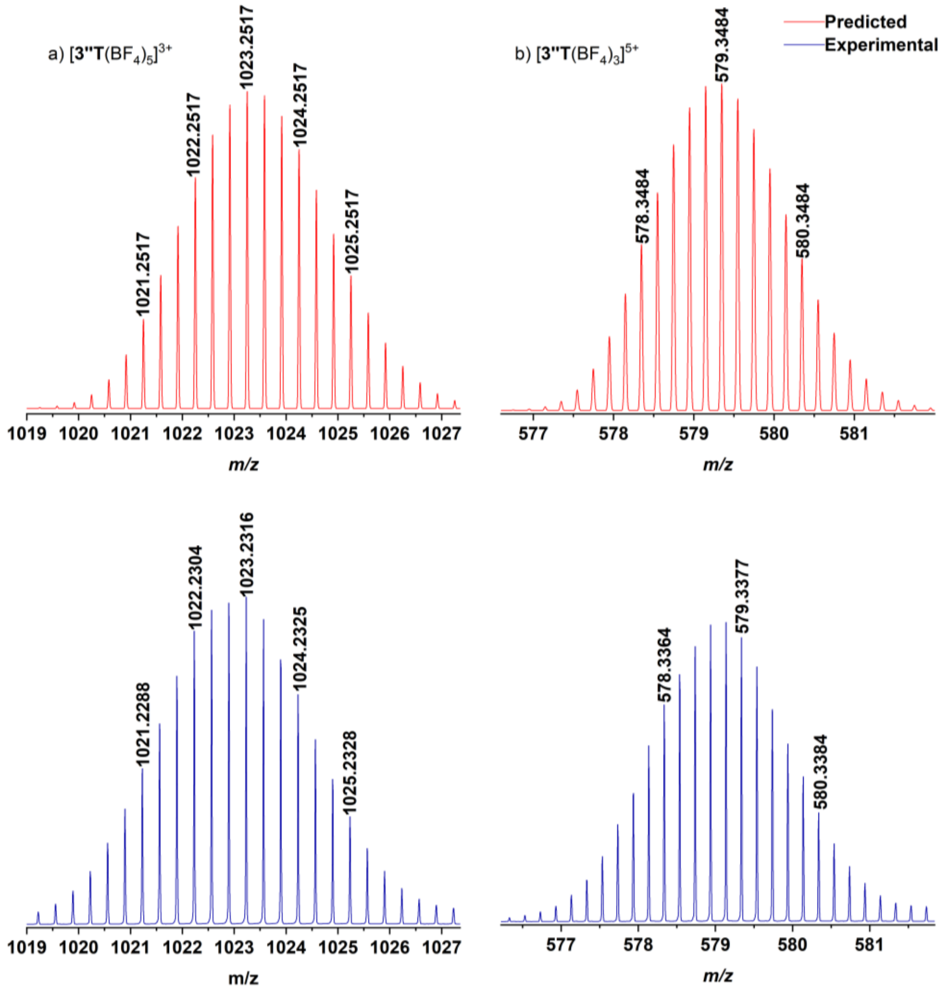

Figure S52: Isotopic distribution of selected peaks from tetrahedral cage **3''T** showing match between predicted and experimentally observed patterns.
(a) [**3''T**$(BF_4)_5]^{3+}$, (b) [**3''T**$(BF_4)_3]^{5+}$

S6.5 Post-assembly modification route to tetrahedral cage [4]$(BF_4)_8$ / $[Pd_4L^6{}_8](BF_4)_8$ and double-walled triangle cage [5]$(BF_4)_6$ / $[Pd_3L^6{}_6](BF_4)_6$

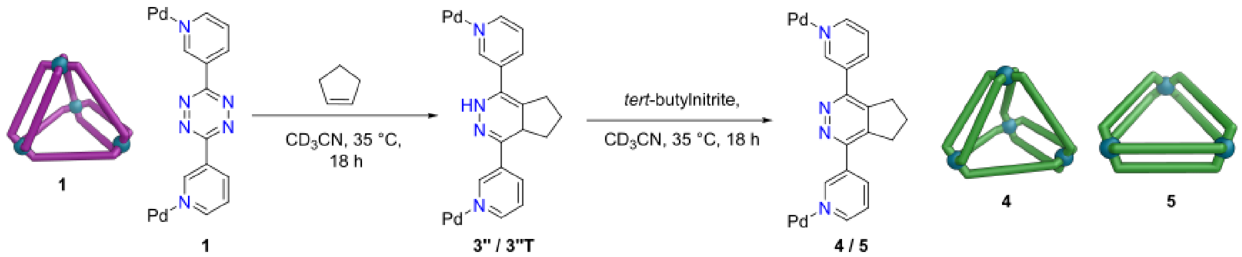

Method 1: Tetrazine cage **1** (4.7 mg, 1.57 µmol) was dissolved in CD_3CN (0.6 mL) and added to cyclopentene (2.6 mg, 3.3 µL, 37.5 µmol) and the solution stirred at 35 ˚C for 18 h to produce a pale-yellow solution of **3''** and **3''T**. To this solution of lantern **3''** and tetrahedron **3''T** in CD_3CN was added *tert*-butylnitrite (0.386 mg, 0.446 µL, 3.75 µmol) and the mixture stirred at 35 ˚C for 18 h. Afterwards the mixture was precipitated with Et_2O (5 mL), washed with Et_2O (3 × 1.5 mL) and then dried *in vacuo* to give a mixture of cages **4** and **5**.

Method 2: Tetrazine cage **1** (4.7 mg, 1.57 µmol) was dissolved in CD_3CN (0.6 mL) and added to cyclopentene (2.6 mg, 3.3 µL, 37.5 µmol) and the solution stirred at 35 ˚C for 18 h to produce a pale-yellow solution of **3''** and **3''T**. The solution was then heated for a further 8 days at 35 ˚C. Afterwards the mixture was precipitated with Et_2O (5 mL), washed with Et_2O (3 × 1.5 mL) and then dried *in vacuo* to give a mixture of cages **4** and **5**.

^1H NMR and ^{13}C NMR, ESI-MS data matched that of the sample prepared from self-assembly of ligand $\mathbf{L^6}$ with $[Pd(CH_3CN)_4](BF_4)_2$ (see Section S5.3).

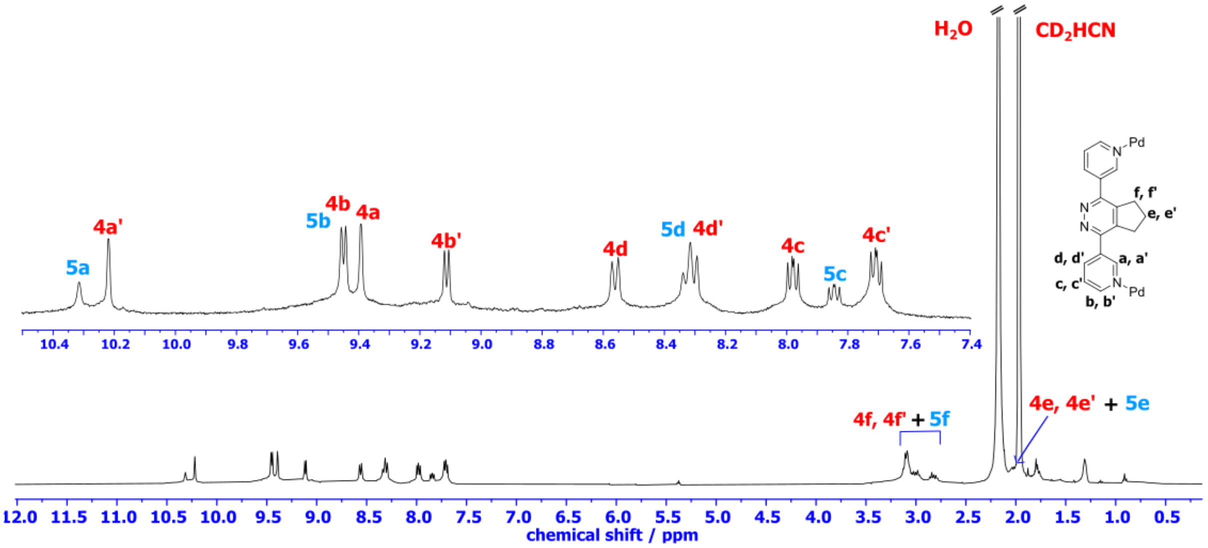

Figure S53: ^1H NMR spectrum of tetrahedral cage **4** and triangular cage **5** produced by *Method 1* (chemical oxidation)

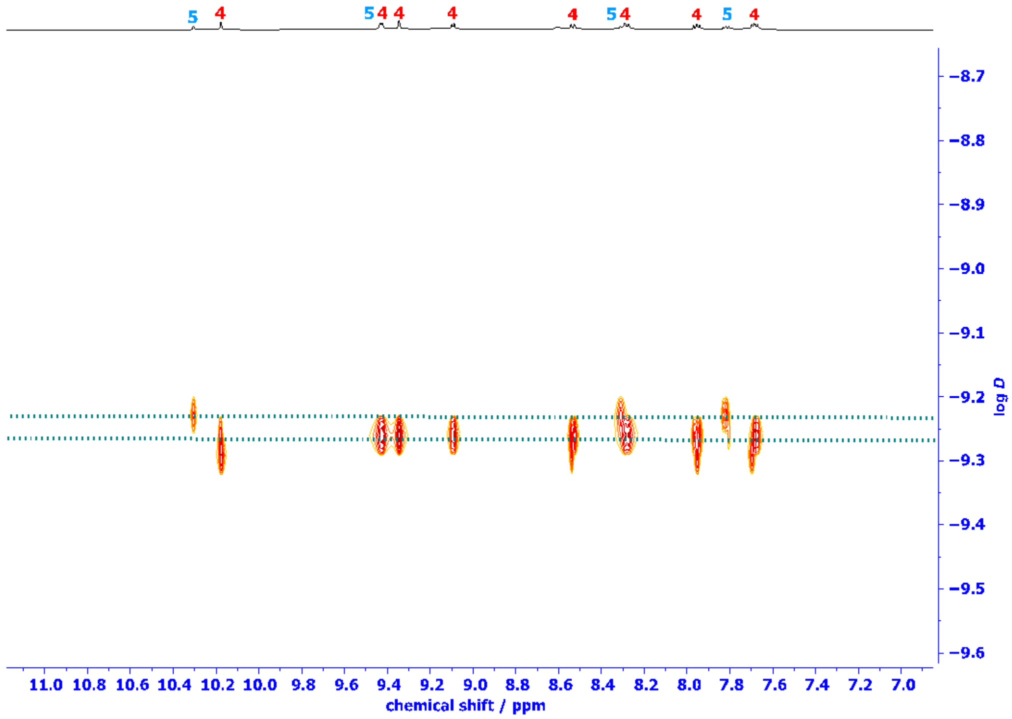

Figure S54: ^{1}H DOSY spectrum of tetrahedral cage **4** and triangular cage **5** (126 MHz, 298 K, CD_3CN)

S7 ESI-MS Studies of Cage Systems

S7.1 ESI-MS studies of the oxidation of dihydropyridazine ligands

The literature consensus was that dihydropyridazines typically undergo a slow oxidation in air to pyridazines, although there has been little study of this process. At the outset of this work, there was also no information on how this process would be affected by the incorporation of this motif in metal-organic cages. As the ^{1}H NMR spectra of the mixtures of lantern and tetrahedral cages were intractably complex, there was little prospect of tracking the oxidation process via ^{1}H NMR spectroscopy. We wondered whether it would be possible to gain any insights from ESI-MS.

Oxidation of a dihydropyridazine ligand to a pyridazine ligand causes a reduction in mass by two units. However, there are several reasons why deducing the number of oxidised ligands present in a sample is challenging. The oxidised ligand is planar, meaning it can only fit into the tetrahedron structure. Firstly, we had to analyse signals from these Pd_4L_8 tetrahedra which were not coincident with the typically much more intense signals from the Pd_2L_4 lanterns, e.g., from the $[Pd_4L_8(BF_4)_5]^{3+}$ ion. Often these signals were only present at lower intensities. Whilst for each oxidised ligand, the mass of L reduces by 2 units, the separation of the peaks where one ligand oxidation has occurred would only be $^{2}/_{3}$ an *m/z* unit given the +3 charge on this this ion. This difference of $^{2}/_{3}$ an *m/z* unit must also be decoupled from the natural isotopic distributions of $^{12}C/^{13}C$, $^{10}B/^{11}B$, and $^{102}Pd/^{104}Pd/^{105}Pd/^{106}Pd/^{108}Pd/^{110}Pd$. It is also possible that structures with varying proportions of oxidised ligands may all be in coexistence as a statistical mixture, rather than a predominant composition.

With these caveats in mind, we compared ESI-MS taken at different points. The maxima in these spectra did reduce over time. Looking at a representative series of the mixture of lantern cage **3''** and tetrahedral cage **3''T** and examining the ESI-MS of the ion $[Pd_4L_8(BF_4)_5]^{3+}$ ion, this oxidation process can be tracked. If the post-assembly modification reaction is setup and performed under Schlenk conditions and a sample only taken out of the J-Young NMR tube immediately before running the MS, the maximum in the experimentally observed ion distribution for the $[Pd_4L_8(BF_4)_5]^{3+}$ ion, corresponds to a mass of where all the ligand is in the dihydropyridazine state, i.e., $[Pd_4L^{5}{}_8(BF_4)_5]^{3+}$ (**Figures S55a**, **S56a**). Where the reaction had been performed in a standard NMR tube, but no rigorous exclusion of oxygen was undertaken, and the ESI-MS was run one day after completion of the reaction, the maxima in the distribution was consistent with the predominant species containing six dihydropyridazine ligands and two pyridazine ligands, i.e., $[Pd_4L^{5}{}_6L^{6}{}_2(BF_4)_5]^{3+}$. In samples that had been left for up to a week before ESI-MS, the maxima in the distribution were consistent with the predominant species containing four dihydropyridazine ligands and four pyridazine ligands,

i.e., $[Pd_4L^5{}_4L^6{}_4(BF_4)_5]^{3+}$ (**Figures S55b**, **S56b**). This composition was consistent with the average composition determined in SCXRD analysis of the crystal of **3''T** (see Section S10.7). One sample monitored over the summer for two weeks (during which time temperatures exceeded 30 °C) appeared to have fully oxidised to the product mixture of cages **4** and **5**, with the maxima in the distribution for this ion now consistent with a $[Pd_4L^6{}_8(BF_4)_5]^{3+}$ composition (**Figure S55c**, **S56c**).

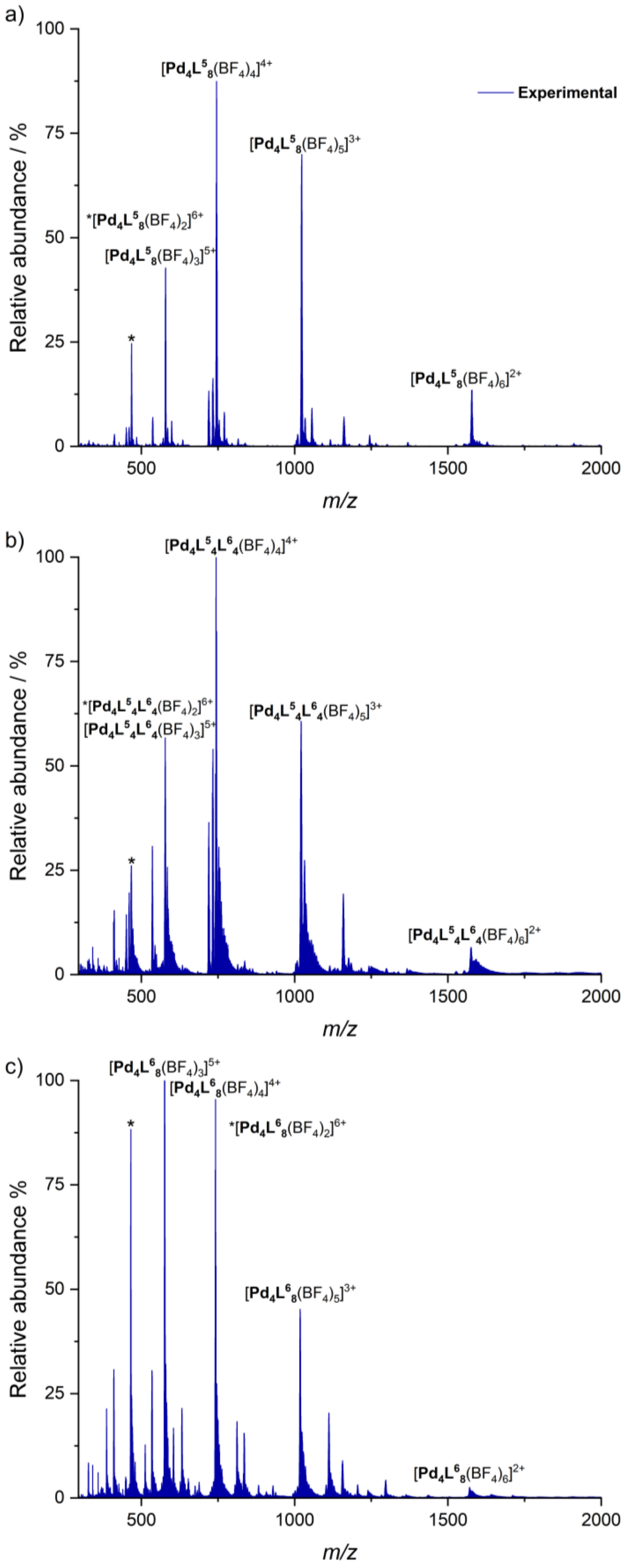

Figure S55: ESI-MS tracking the oxidation of ligand $\mathbf{L^5}$ to $\mathbf{L^6}$ over time.
(a) sample only taken out of the J-Young NMR tube immediately before running MS; (b) sample left for a week before MS; (c) sample left for two weeks over summer at 30 °C before MS

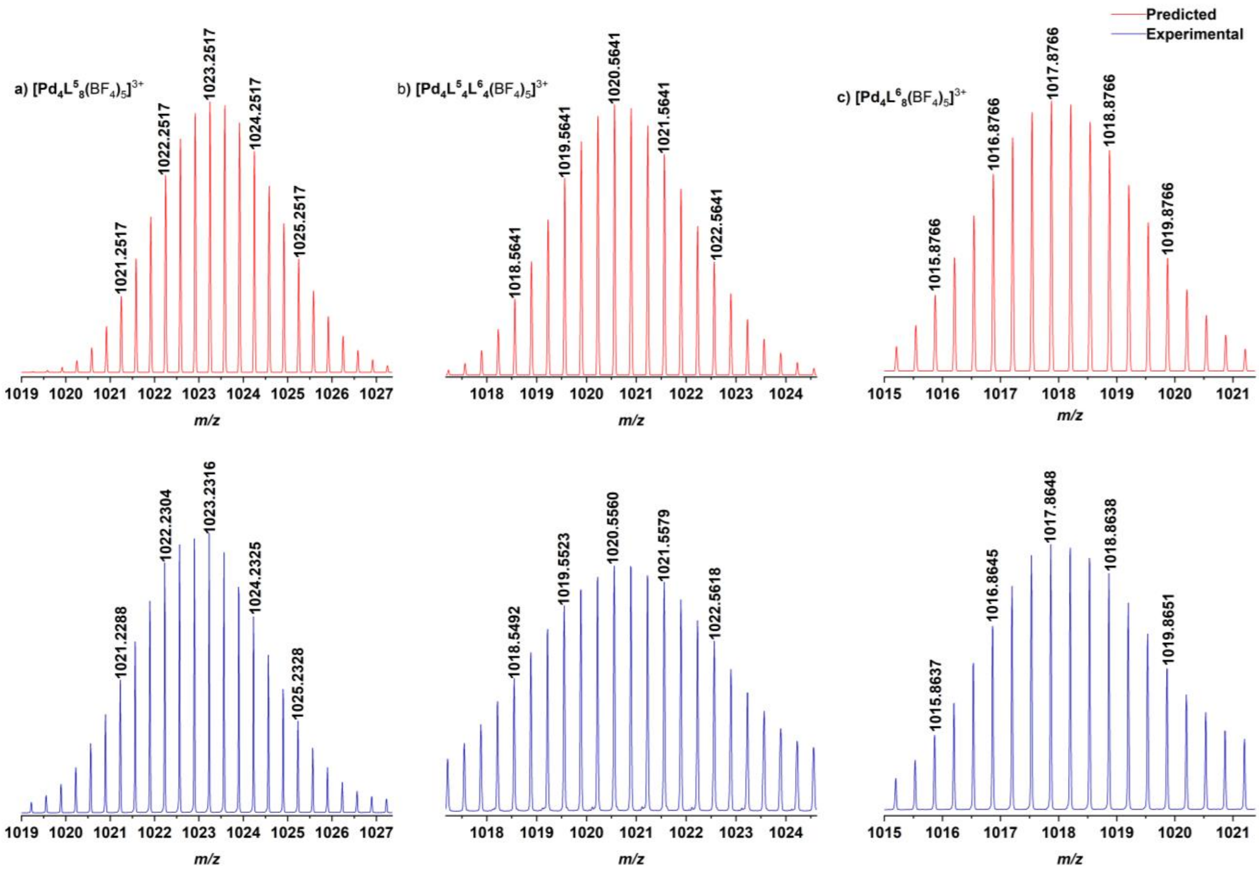

Figure S56: ESI-MS tracking the oxidation of ligand **L^5** to **L^6** over time. Isotopic distribution patterns showing average composition of $[Pd_4L_8(BF_4)_5]^{3+}$ peak approximating to: (a) $[Pd_4L^5{}_8(BF_4)_5]^{3+}$ in sample only taken out of the J-Young NMR tube immediately before running MS; (b) $[Pd_4L^5{}_4L^6{}_4(BF_4)_5]^{3+}$ in sample left for a week before MS; (c) $[Pd_4L^6{}_8(BF_4)_5]^{3+}$ in sample left for two weeks over summer at 30 °C before MS

S7.2 ESI-MS studies on whether post-assembly modification led to thermodynamic mixtures of cages or kinetically trapped mixtures

Post-assembly modification with an alkene leads to a dihydropyridazine ligand which is significantly more flexible than the starting tetrazine ligand **L^1**. Whilst there is an entropic driving force to form structures of lower nuclearity, the greater flexibility in the metal-ligand coordination vector angles in dihydropyridazine ligands **L^3**, **L^4**, and **L^5** mean that these ligands can also adopt the geometry required for tetrahedral cages. Our working hypothesis was thus that the reaction between the tetrazine and alkene was a post-assembly modification on complex, to give initially the dihydropyridazine tetrahedra (**3T**, **3'T**, or **3''T**). Whilst the ^{1}H NMR spectra were too complex to determine any ratios between structures, we looked at changes in the ESI-MS peak distributions over time, to determine whether the proportion of structures was changing, and if this gave any insights into whether the observed structures were kinetically trapped products or thermodynamic mixtures. Whilst ESI-MS is not quantitative due

to possible different response factors, relative changes in spectra can indicate changes in mixture composition.

Given our earlier observations that slow oxidation of the dihydropyridazine ligands $\mathbf{L^3}$, $\mathbf{L^4}$, and $\mathbf{L^5}$ occurred in air, the post-assembly modification of the following samples was performed under inert conditions in Schlenk tubes in degassed solvents, with the samples only removed from the Schlenk tubes immediately before running the ESI-MS. For reference, complete oxidation of ligand $\mathbf{L^5}$ to $\mathbf{L^6}$ occurs over a period of seven days at 35 ˚C, for a sample left in a standard NMR tube (not in a Schlenk tube) due to oxidation from adventitious oxygen in the air, leading to a transformation of the mixture of lantern cage **3''** and tetrahedral cage **3''T** to a mixture of tetrahedral cage **4** and lantern cage **5**.

S7.2.1 Lantern cage 3 and tetrahedral cage 3T

ESI-MS analysis immediately after post-assembly modification of tetrahedral cage **1** with norbornene to give lantern cage **3** and tetrahedral cage **3T**, showed a mixture containing both lantern cage **3** and tetrahedral cage **3T** (**Figure S57**, top). This was consistent with the hypothesis that post-assembly modification occurred on complex to initially convert all tetrahedral cage **1** to tetrahedral cage **3T**, but that there was a reasonably low barrier to interconversion, allowing significant amounts of material to have converted to lantern cage **3** before the ESI-MS analysis was run. Further ESI-MS analysis after 13 days at 298 K under inert conditions, indicated that the mixture had converted almost entirely to lantern cage **3**, with only trace baseline signals remaining from tetrahedral cage **3T** (**Figure S57**, bottom). This is consistent with the smaller nuclearity lantern cage **3** being the thermodynamic product of this system (entropic driving force to from assemblies with fewer components). The steric bulk of the added substituent from the norbornene would likely help drive this change, as the double-walled edges in tetrahedral cage **3T** would possess two of these added substituents in close proximity.

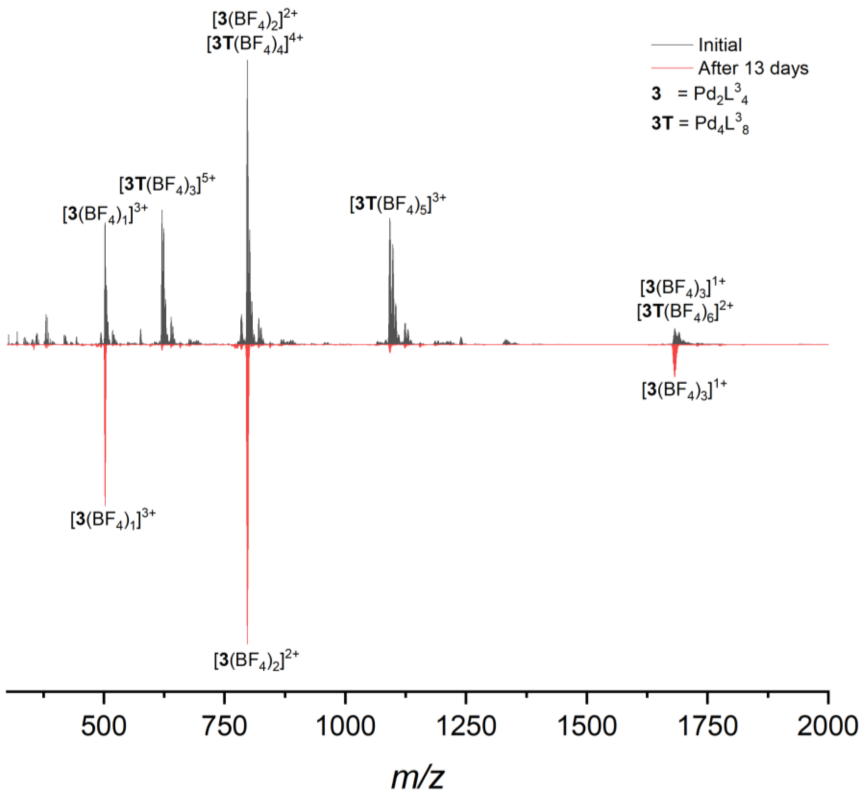

Figure S57: ESI-MS of norbornene functionalised lantern cage **3** and tetrahedral cage **3T** Spectra taken immediately after post-assembly modification under inert conditions (black, top) and 13 days after post-assembly modification under inert conditions (red, bottom)

S7.2.2 Lantern cage 3' and tetrahedral cage 3'T

ESI-MS analysis immediately after post-assembly modification of tetrahedral cage **1** with 5-norbornene-2-carboxylic acid to give lantern cage **3'** and tetrahedral cage **3'T**, showed a mixture containing almost entirely signals from lantern cage **3'**, with only baseline signals from tetrahedral cage **3'T** (**Figure S58**, top). We attribute this again to post-assembly modification having occurred on complex, initially converting all tetrahedral cage **1** to tetrahedral cage **3'T**, but that the greater steric bulk of the added norbornene acid substituent driving a quick rearrangement to lantern cage **3'**, over a low energetic barrier before the ESI-MS analysis was run. Further ESI-MS analysis after 13 days at 298 K under inert conditions, gave a spectrum that looked almost identical (**Figure S58**, bottom). This indicated that the thermodynamic equilibrium had likely already been reached in the initial ESI-MS, and that this equilibrium composition had most of the material in the smaller nuclearity lantern **3'** as would be expected. The difference between the norbornene system (**3**/**3T**) and the norbornene acid system (**3'**/**3'T**) was thus that much faster rearrangement seemed to be occurring in the norbornene acid system, with the equilibrium also likely favouring a greater proportion of the lantern. Both can be rationalised by the greater steric bulk of the norbornene acid.

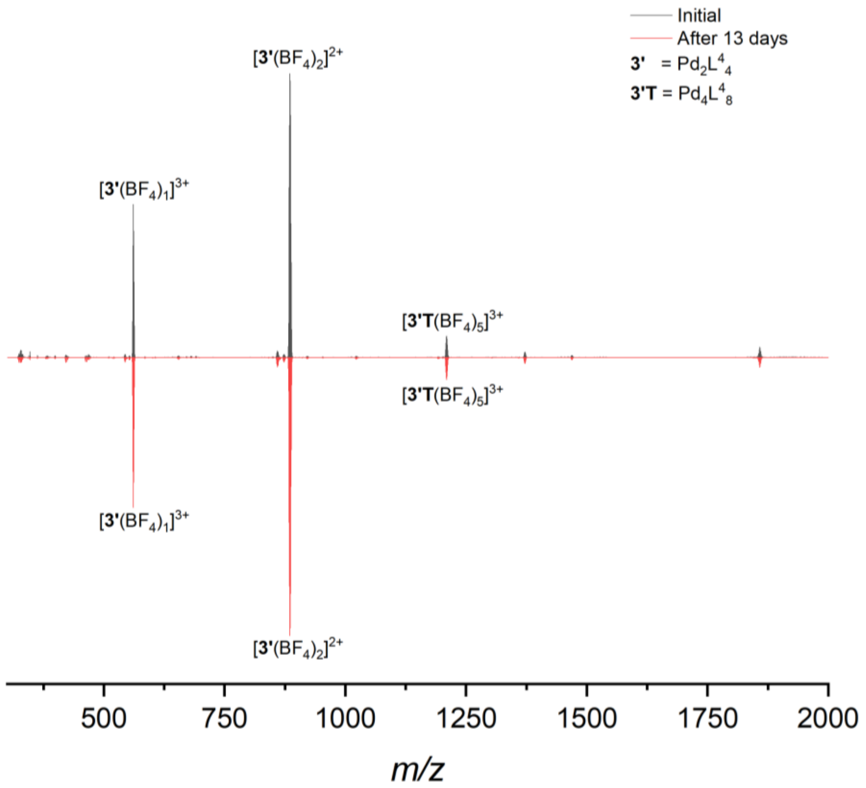

Figure S58: ESI-MS of norbornene acid functionalised lantern cage **3'** and tetrahedral cage **3'T**
Spectra taken immediately after post-assembly modification under inert conditions (black, top) and 13 days after post-assembly modification under inert conditions (red, bottom)

S7.2.3 Lantern cage 3'' and tetrahedral cage 3''T

ESI-MS analysis immediately after post-assembly modification of tetrahedral cage **1** with cyclopentene to give lantern cage **3''** and tetrahedral cage **3''T**, showed a mixture containing signals from lantern cage **3''**, tetrahedral cage **3''T**, and also a $Pd_3L^5_6$ double-walled triangular cage **3''Tri** (Figure S59, top). This triangular cage **3''Tri** is constructed from the dihydropyridazine ligands $\mathbf{L^5}$ but has the same structure as the oxidised product triangular cage **5** that is constructed from the oxidised pyridazine ligands $\mathbf{L^6}$. Again, the greater flexibility of the $\mathbf{L^5}$ ligand allows it to adopt different structural geometries. We attribute the fact that this **3''Tri** structure is only seen in the cyclopentene-modified system due to the lower steric bulk of the added cyclopentene substituent, given that **3''Tri** possesses double-walled edges. Whilst ESI-MS analysis is not quantitative, comparing the results of this system to the others, there appears to be a greater proportion of tetrahedral cage **3''T** in the initial spectrum for this system. This is consistent with the smaller cyclopentene substituent providing a smaller driving force for rearrangement, and/or the equilibrium mixture containing a higher proportion of tetrahedral cage **3''T**, again perhaps due to the lower steric demand of the substituent. Further ESI-MS analysis after 13 days at 298 K under inert conditions, gave a spectrum that still

showed all three types of structures: lantern cage **3’’**, tetrahedral cage **3’’T**, and triangular cage **3’’Tri** (**Figure S59**, bottom). There were some small changes in intensity, with peaks solely from tetrahedral cage **3’’T** decreasing in intensity and peaks from lantern cage **3’’** and triangular cage **3’’Tri** increasing in intensity. This is consistent with the equilibrium mixture containing a greater proportion of these lower nuclearity structures, but perhaps also the barrier to rearrangement being larger due to the lower steric bulk of the cyclopentyl substituent.

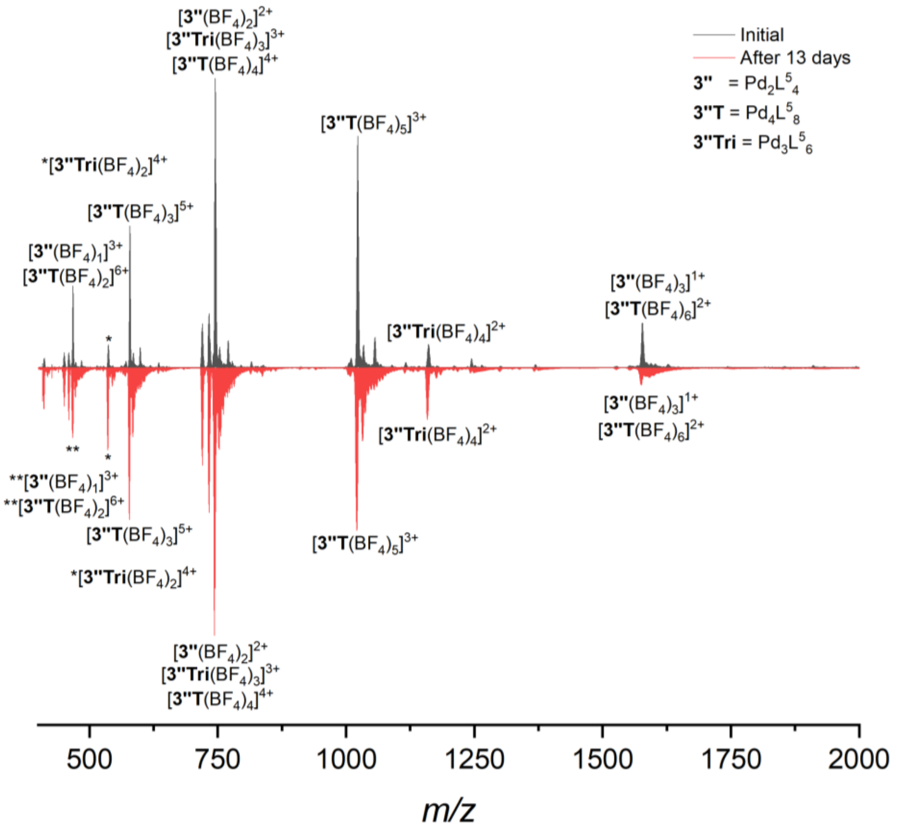

Figure S59: ESI-MS of cyclopentene functionalised lantern cage **3’’**, tetrahedral cage **3’’T**, and triangular cage **3’’Tri**
Spectra taken immediately after post-assembly modification under inert conditions (black, top) and 13 days after post-assembly modification under inert conditions (red, bottom)

S7.2.4 Lantern cage 3’’ and tetrahedral cage 3’’T at higher temperatures

As ESI-MS analysis of the sample of the cyclopentene modified system still showed appreciable proportions of tetrahedral cage **3’’T** after 13 days at 298 K under inert conditions, we performed another experiment where the mixture was heated. As before, the ESI-MS ran immediately after post-assembly modification under inert conditions showed a mixture containing signals from lantern cage **3’’**, tetrahedral cage **3’’T**, and triangular cage **3’’Tri** (**Figure S60**, top). A second sample synthesised at the same time was then heated for 18 h at 338 K under inert conditions and further ESI-MS analysis was performed (**Figure S60**,

bottom). This spectrum showed even lower amounts of the tetrahedral cage **3’’T** and higher amounts of lantern cage **3’’** and triangular cage **3’’Tri** than the sample that had been left for 13 days at 298 K. This indicated that there was likely a higher barrier to rearrangement in this cyclopentene system and that the equilibrium composition had not been reached after 13 days at 298 K. The fact that all three structural types were still present after heating for 18 h at 338 K, indicated the lower steric bulk of the cyclopentyl substituent, combined with the flexibility of the dihydropyridazine ligand make all structures energetically accessible in this system.

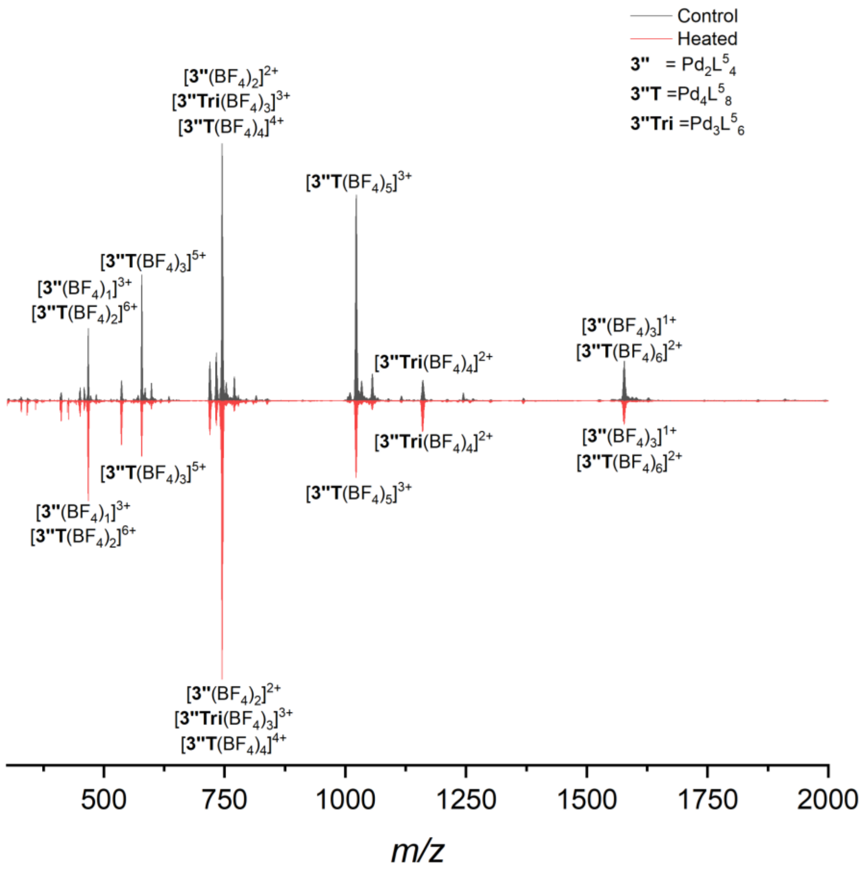

Figure S60: ESI-MS of cyclopentene functionalised lantern cage **3’’**, tetrahedral cage **3’’T**, and triangular cage **3’’Tri**
Spectra taken immediately after post-assembly modification under inert conditions (black, top) and after heating at 338 K for 18 h under inert conditions (red, bottom)

S8 Determination of bend angles and ligand vector angles

The angles quoted in this work were determined from the single crystal X-ray diffraction data obtained. In the processing of the X-ray data, atom labels were assigned to each atom in the final Crystallographic Information File (.cif). These files were then opened within PyMOL. A single atom at a time was selected in the PyMOL interface and the following command was used to retrieve the (x,y,z) coordinates of that atom.

xyz = cmd.get_coords('sele', 1),

print xyz

The atom coordinates of all atoms in question were tabulated in Excel. 3D Cartesian vectors were defined from the position of one atom to another atom. For the vectors for calculation of the angle α between the adjoining bonds at the 3 and 6-positions, the start of each vector was on the 3 or 6-ring atom and the end of each vector was on the 3-position of the pyridine ring. The resultant 3D Cartesian vectors **A**(x,y,z) and **B**(x,y,z) were calculated through subtraction of the coordinates of the starting atom from the coordinates of the final atom.

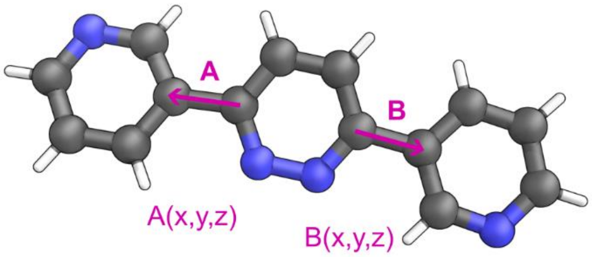

Figure S61: Vectors for calculation of the angle, α, between bonds to adjoining rings

For the vectors for calculation of the metal-ligand coordination vector angle, Θ, the start of each vector was on the pyridine nitrogen atom and the end of each vector was on the palladium atom. The resultant 3D Cartesian vectors **A**(x,y,z) and **B**(x,y,z) were calculated through subtraction of the coordinates of the starting atom from the coordinates of the final atom.

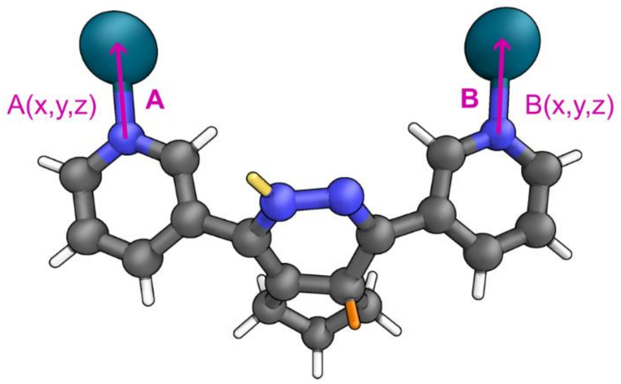

Figure S62: Vectors for calculation of the metal-ligand coordination vector angle, Θ

As the two vectors **A**(x,y,z) and **B**(x,y,z) are not coplanar, the following mathematical formulae were used to calculate the angle between the two 3D Cartesian vectors, with the vector components denoted Ax, Ay, Az, Bx, By, and Bz respectively.

$$\alpha = \cos^{-1}\frac{(AxBx + AyBy + AzBz)}{\sqrt{Ax^2 + Ay^2 + Az^2} * \sqrt{Bx^2 + By^2 + Bz^2}}$$

$$\Theta = \cos^{-1}\frac{(AxBx + AyBy + AzBz)}{\sqrt{Ax^2 + Ay^2 + Az^2} * \sqrt{Bx^2 + By^2 + Bz^2}}$$

Within each structure, each crystallographically independent value of the angles α and Θ were calculated. Mean angles α_{av} and Θ_{av} were then calculated for either the overall structure where values were similar, or separately for single-walled and double-walled edges in cases where the values were substantially different. The standard deviation in the values was also calculated. The coordinates, vectors and angles for each structure are tabulated below. The numbering of the atoms follows the same numbering convention as the numbers assigned in the Crystallographic Information File (.cif).

S8.1 Cage 1

Single-walled edges			
**Atom/Vector**	**x**	**y**	**z**
Pd1	13.47	19.61	8.97
N11A	14.56	21.16	9.66
**A vector**	**−1.09**	**−1.56**	**−0.69**
Pd4	20.17	21.08	19.11
N31A	20.89	21.19	17.22
**B vector**	**−0.72**	**−0.11**	**1.90**
**Θ**			**94.89°**
Pd4	20.17	21.08	19.11
N11B	20.87	19.18	19.24
**A vector**	**−0.70**	**1.90**	**−0.13**
Pd3	13.64	11.17	16.10
N31B	15.07	11.28	17.52
**B vector**	**−1.43**	**−0.11**	**−1.43**
**Θ**			**76.32°**
Pd3	13.64	11.17	16.10
N11C	12.16	11.33	17.47
**A vector**	**1.48**	**−0.17**	**−1.38**
Pd2	8.93	21.87	19.86
N31C	8.09	20.06	20.11
**B vector**	**0.85**	**1.82**	**−0.25**
**Θ**			**71.59°**
Pd1	13.47	19.61	8.97
N31D	11.75	20.61	9.21
**A vector**	**1.72**	**−1.00**	**−0.25**
Pd2	8.93	21.87	19.86
N11D	7.91	22.20	18.14
**B vector**	**1.03**	**−0.32**	**1.72**
**Θ**			**65.95°**

Double-walled edges

Atom/Vector	x	y	z
Pd1	13.47	19.61	8.97
N11E	15.15	18.49	8.79
**A vector**	**−1.69**	**1.11**	**0.17**
Pd3	13.64	11.17	16.10
N31E	15.12	11.09	14.72
**B vector**	**−1.49**	**0.07**	**1.38**
**Θ**			**46.62°**
Pd1	13.47	19.61	8.97
N11F	12.29	18.05	8.37
**A vector**	**1.17**	**1.56**	**0.60**
Pd3	13.64	11.17	16.10
N31F	12.22	10.97	14.66
**B vector**	**1.42**	**0.20**	**1.44**
**Θ**			**46.99°**
Pd2	8.93	21.87	19.86
N11G	9.92	21.60	21.61
**A vector**	**−0.99**	**0.27**	**−1.75**
Pd4	20.17	21.08	19.11
N31G	19.37	20.89	20.98
**B vector**	**0.81**	**0.18**	**−1.87**
**Θ**			**52.56°**
Pd2	8.93	21.87	19.86
N11H	9.87	23.65	19.62
**A vector**	**−0.94**	**−1.77**	**0.25**
Pd4	20.17	21.08	19.11
N31H	19.42	22.95	18.92
**B vector**	**0.75**	**−1.87**	**0.19**
**Θ**			**49.49°**

Single-walled edges			
**Atom/Vector**	**x**	**y**	**z**
C13A	16.30	21.99	11.09
C21A	17.44	21.65	12.02
**A vector**	**−1.14**	**0.34**	**−0.93**
C33A	20.35	21.21	14.89
C24A	19.38	21.30	13.78
**B vector**	**0.97**	**−0.09**	**1.11**
**α**			**166.85°**
C33B	16.88	12.44	18.57
C24B	17.67	13.69	18.72
**A vector**	**−0.79**	**−1.25**	**−0.15**
C13B	20.33	16.85	19.13
C21B	19.31	15.77	19.00
**B vector**	**1.02**	**1.07**	**0.13**
**α**			**168.92°**
C33C	8.19	17.68	19.87
C24C	8.90	16.47	19.40
**A vector**	**−0.71**	**1.21**	**0.48**
C13C	10.60	12.77	18.58
C21C	10.05	14.15	18.80
**B vector**	**0.56**	**−1.39**	**−0.22**
**α**			**166.83°**
C33D	10.03	21.33	10.68
C24D	9.50	21.40	12.06
**A vector**	**0.52**	**−0.07**	**−1.39**
C13D	7.80	22.15	15.76
C21D	8.42	21.78	14.47
**B vector**	**−0.62**	**0.37**	**1.29**
**α**			**167.19°**

Double-walled edges

Atom/Vector	x	y	z
C13E	16.49	16.69	9.62
C21E	16.61	15.52	10.54
**A vector**	**−0.12**	**1.17**	**−0.92**
C33E	16.40	12.20	13.01
C24E	16.54	13.35	12.13
**B vector**	**−0.14**	**−1.16**	**0.88**
**α**			**170.01°**
C13F	11.23	16.03	9.00
C21F	11.05	15.05	10.08
**A vector**	**0.19**	**0.97**	**−1.08**
C33F	11.13	11.86	12.73
C24F	11.00	13.05	11.84
**B vector**	**0.13**	**−1.19**	**0.89**
**α**			**163.38°**
C33G	17.48	20.60	22.41
C24G	16.01	20.57	22.57
**A vector**	**1.47**	**0.04**	**−0.17**
C13G	11.91	21.08	22.81
C21G	13.35	20.78	22.77
**B vector**	**−1.44**	**0.31**	**0.05**
**α**			**165.89°**
C33H	17.50	24.37	18.73
C24H	16.03	24.46	18.68
**A vector**	**1.48**	**−0.09**	**0.05**
C13H	11.94	24.79	19.19
C21H	13.38	24.65	18.88
**B vector**	**−1.44**	**0.14**	**0.31**
**α**			**165.85°**

S8.2 Cage 2

Single-walled edges			
**Atom/Vector**	**x**	**y**	**z**
Pd1	8.46	5.68	12.11
N21A	10.17	4.64	11.87
**A vector**	**−1.71**	**1.03**	**0.24**
Pd2	12.98	3.41	1.21
N11A	14.00	3.08	2.93
**B vector**	**−1.02**	**0.33**	**−1.72**
**Θ**			**65.77°**
Pd4	8.31	14.09	4.99
N21B	9.78	13.94	3.61
**A vector**	**−1.47**	**0.16**	**1.38**
Pd2	12.98	3.41	1.21
N11B	13.85	5.22	0.96
**B vector**	**−0.87**	**−1.81**	**0.25**
**Θ**			**71.08°**
Pd4	8.31	14.09	4.99
N21C	6.87	14.00	3.55
**A vector**	**1.44**	**0.10**	**1.44**
Pd3	1.77	4.22	1.96
N11C	1.08	6.12	1.84
**B vector**	**0.69**	**−1.90**	**0.12**
**Θ**			**76.16°**
Pd1	8.46	5.68	12.11
N21D	7.36	4.13	11.42
**A vector**	**1.10**	**1.55**	**0.68**
Pd3	1.77	4.22	1.96
N11D	1.07	4.08	3.85
**B vector**	**0.71**	**0.14**	**−1.89**
**Θ**			**94.13°**

Double-walled edges

Atom/Vector	x	y	z
Pd3	1.77	4.22	1.96
N21E	2.57	4.40	0.10
**A vector**	**−0.79**	**−0.18**	**1.86**
Pd2	12.98	3.41	1.21
N11E	12.01	3.69	−0.53
**B vector**	**0.97**	**−0.28**	**1.74**
**Θ**			**51.97°**
Pd3	1.77	4.22	1.96
N21F	2.52	2.35	2.13
**A vector**	**−0.75**	**1.88**	**−0.17**
Pd2	12.98	3.41	1.21
N11F	12.05	1.64	1.45
**B vector**	**0.94**	**1.77**	**−0.24**
**Θ**			**49.24°**
Pd4	8.31	14.09	4.99
N21G	9.71	14.28	6.43
**A vector**	**−1.40**	**−0.19**	**−1.44**
Pd1	8.46	5.68	12.11
N11G	9.62	7.24	12.70
**B vector**	**−1.16**	**−1.56**	**−0.60**
**Θ**			**47.32°**
Pd4	8.31	14.09	4.99
N21H	6.80	14.16	6.35
**A vector**	**1.51**	**−0.07**	**−1.36**
Pd1	8.46	5.68	12.11
N11H	6.77	6.80	12.28
**B vector**	**1.69**	**−1.13**	**−0.18**
**Θ**			**46.29°**

Single-walled edges			
**Atom/Vector**	**x**	**y**	**z**
C23A	11.85	3.88	10.39
C34A	12.37	3.80	8.98
**A vector**	**−0.52**	**0.08**	**1.41**
C13A	14.11	3.11	5.29
C31A	13.44	3.42	6.58
**B vector**	**0.67**	**−0.31**	**−1.28**
**α**			**168.43°**
C23B	11.33	12.51	2.47
C34B	11.85	11.14	2.20
**A vector**	**−0.52**	**1.37**	**0.27**
C13B	13.75	7.59	1.20
C31B	13.00	8.81	1.62
**B vector**	**0.74**	**−1.22**	**−0.42**
**α**			**167.91°**
C23C	5.07	12.86	2.51
C31C	4.26	11.60	2.36
**A vector**	**0.81**	**1.25**	**0.15**
C13C	1.61	8.47	1.94
C34C	2.63	9.52	2.08
**B vector**	**−1.02**	**−1.05**	**−0.14**
**α**			**168.99°**
C23D	5.59	3.32	9.99
C34D	4.59	3.64	9.00
**A vector**	**1.01**	**−0.32**	**0.99**
C13D	1.60	4.06	6.18
C31D	2.63	3.99	7.23
**B vector**	**−1.03**	**0.07**	**−1.04**
**α**			**170.17°**

Double-walled edges			
**Atom/Vector**	**x**	**y**	**z**
C23E	4.44	4.67	−1.36
C31E	5.93	4.70	−1.50
**A vector**	**−1.49**	**−0.03**	**0.14**
C13E	10.02	4.20	−1.74
C34E	8.56	4.48	−1.70
**B vector**	**1.45**	**−0.28**	**−0.04**
**α**			**167.35°**
C23F	4.43	0.92	2.34
C34F	5.91	0.83	2.40
**A vector**	**−1.48**	**0.09**	**−0.05**
C13F	9.99	0.50	1.85
C31F	8.54	0.62	2.16
**B vector**	**1.45**	**−0.12**	**−0.31**
**α**			**166.02°**
C23G	10.84	13.40	8.36
C34G	10.98	12.26	9.29
**A vector**	**−0.14**	**1.13**	**−0.93**
C13G	10.71	9.24	12.11
C31G	10.95	10.27	11.07
**B vector**	**−0.24**	**−1.03**	**1.04**
**α**			**163.91°**
C23H	5.53	13.09	8.04
C34H	5.38	11.90	8.94
**A vector**	**0.16**	**1.19**	**−0.90**
C13H	5.42	8.62	11.45
C31H	5.33	9.79	10.56
**B vector**	**0.09**	**−1.17**	**0.90**
**α**			**170.48°**

S8.3 Cage 3

Atom/Vector	x	y	z
Pd1	3.68	6.92	9.28
N11	2.75	8.34	10.32
**A vector**	**0.93**	**−1.42**	**−1.04**
Pd1	9.62	6.92	15.42
N21	8.55	8.30	16.36
**B vector**	**1.07**	**−1.39**	**−0.94**
**ϴ**			**5.15°**
Pd1	3.68	6.92	9.28
N21	4.75	8.30	8.34
**A vector**	**−1.07**	**−1.39**	**0.94**
Pd1	9.62	6.92	15.42
N11	10.55	8.34	14.38
**B vector**	**−0.93**	**−1.42**	**1.04**
**ϴ**			**5.15°**
Pd1	3.68	6.92	9.28
N21	4.75	5.53	8.34
**A vector**	**−1.07**	**1.39**	**0.94**
Pd1	9.62	6.92	15.42
N11	10.55	5.49	14.38
**B vector**	**−0.93**	**1.42**	**1.04**
**ϴ**			**5.15°**
Pd1	3.68	6.92	9.28
N11	2.75	5.49	10.32
**A vector**	**0.93**	**1.42**	**−1.04**
Pd1	9.62	6.92	15.42
N21	8.55	5.53	16.36
**B vector**	**1.07**	**1.39**	**−0.94**
**ϴ**			**5.15°**

Due to a high amount of unfixable disorder in the SCXRD data of lantern cage **3**, uncertainties around individual atom hybridization and stereochemistry at the carbon centres mean that calculation of the average angle, α_{av}, between adjoining C–C bonds would not be meaningful from the data available for this structure, so these values have not been tabulated. Due to the asymmetric unit of the final structure being one quarter of cage **3**, all tabulated values of the metal-ligand coordination vector, ϴ, are the same. Hence whilst this ϴ value represents a true average from the data, an equivalent standard deviation cannot be determined.

Two views of a representation of the crystal structure are shown in **Figure S63**. The hydrogen atoms of the 1,4-dihydropyridazine rings have been omitted due to uncertainty of the atom hybridization and stereochemistry at the carbon stereocenters in these rings. Although the location of the N–H groups cannot be determined, the relative orientation of these rings strongly suggests two pairs of N–H to N hydrogen bonds, with the ligands in head to tail orientation. The average distance between the nitrogen atoms is 3.19 Å. This is again

consistent with hydrogen bonding, although we note restraints put on the rings during data analysis may have influenced this value. There is considerable disorder in the ligand position, and for clarity only one orientation is shown here. In this orientation, the one carbon bridges of the norbornyl substituent are orientated towards each other. This can be easily rationalized based on steric arguments. Whilst the data are certainly consistent with this isomer being the sole or major isomer in the crystal structure, the additional presence of other isomers cannot be excluded due to the disorder (see Section S10.5).

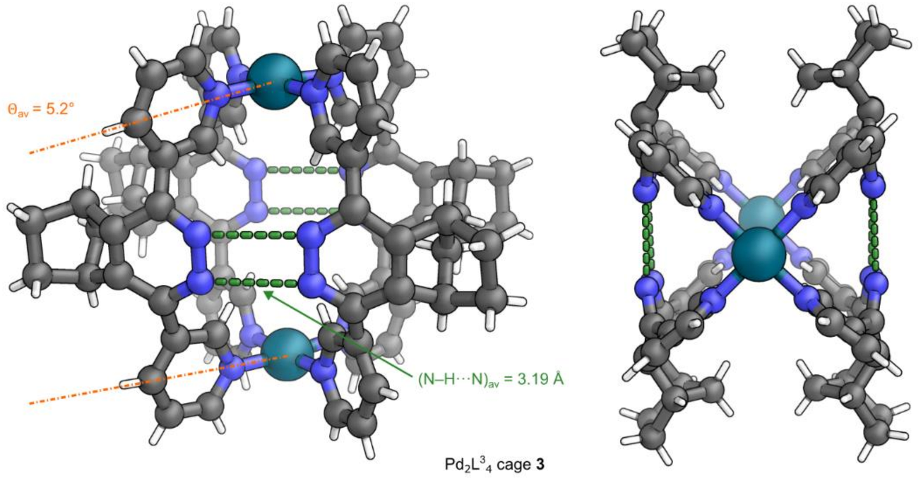

Figure S63: Two views of the SCXRD structure of lantern cage **3**
Disorder, counteranions, and solvent have been omitted for clarity. Hydrogens on the 1,4-dihydropyridazine rings have been omitted due to uncertainty over atom hybridization and stereochemistry of the carbon stereocenters in these rings. Colour: C = grey, N = blue, Pd = turquoise

S8.4 Cage 3''

Atom/Vector	x	y	z
Pd1	−3.67	0.56	10.19
N21A	−2.13	0.27	8.90
**A vector**	**−1.54**	**0.29**	**1.30**
Pd1	−5.06	8.37	6.36
N11A	−3.45	7.87	5.21
**B vector**	**−1.61**	**0.50**	**1.15**
**Θ**			**7.31°**
Pd1	−5.06	8.37	6.36
N21B	−3.70	9.14	7.68
**A vector**	**−1.36**	**−0.77**	**−1.32**
Pd1	−3.67	0.56	10.19
N11B	−2.33	1.55	11.40
**B vector**	**−1.34**	**−0.99**	**−1.21**
**Θ**			**7.04°**
Pd2	4.85	11.56	0.75
N21C	5.02	11.71	−1.29
**A vector**	**−0.17**	**−0.16**	**2.03**
Pd2	3.74	2.95	−0.75
N11C	3.94	3.37	−2.73
**B vector**	**−0.20**	**−0.42**	**1.99**
**Θ**			**7.62°**
Pd2	4.85	11.56	0.75
N11D	6.86	11.24	0.83
**A vector**	**−2.00**	**0.32**	**−0.08**
Pd2	3.74	2.95	−0.75
N21D	5.77	2.87	−0.61
**B vector**	**−2.03**	**0.09**	**−0.14**
**Θ**			**6.79°**

Atom/Vector	x	y	z
C13A	−1.65	6.33	4.88
C32A	−1.00	5.08	5.31
**A vector**	**−0.65**	**1.26**	**−0.43**
C23A	−0.84	1.36	7.20
C38A	−0.61	2.64	6.49
**B vector**	**−0.23**	**−1.28**	**0.71**
**α**			**143.84°**
C23B	−2.02	8.51	9.28
C38B	−1.35	7.40	9.98
**A vector**	**−0.67**	**1.11**	**−0.70**
C13B	−1.16	3.63	11.73
C32B	−0.93	5.00	11.24
**B vector**	**−0.23**	**−1.37**	**0.49**
**α**			**142.12°**
C13C	4.61	5.09	−4.30
C32C	5.12	6.46	−4.45
**A vector**	**−0.51**	**−1.37**	**0.16**
C23C	5.30	10.46	−3.34
C38C	5.51	9.12	−3.93
**B vector**	**−0.21**	**1.33**	**0.60**
**α**			**138.37°**
C13D	8.54	9.58	0.40
C32D	8.79	8.19	−0.02
**A vector**	**−0.25**	**1.39**	**0.42**
C23D	7.78	4.14	−0.41
C38D	8.35	5.51	−0.40
**B vector**	**−0.57**	**−1.36**	**−0.01**
**α**			**143.71°**

S8.5 Cage 4

Double-walled edges			
**Atom/Vector**	**x**	**y**	**z**
Pd1	25.67	9.77	29.40
N21A	25.27	9.09	31.21
**A vector**	**0.40**	**0.68**	**−1.81**
Pd4	25.22	−1.14	29.90
N11A	25.33	−0.48	31.74
**B vector**	**−0.11**	**−0.66**	**−1.84**
**Θ**			**42.64°**
Pd1	25.67	9.77	29.40
N21B	27.47	9.22	29.45
**A vector**	**−1.80**	**0.55**	**−0.05**
Pd4	25.22	−1.14	29.90
N11B	27.08	−0.58	29.62
**B vector**	**−1.87**	**−0.56**	**0.28**
**Θ**			**35.13°**
Pd2	22.56	4.82	19.29
N21C	21.50	3.17	19.32
**A vector**	**1.06**	**1.65**	**−0.03**
Pd3	14.93	4.14	27.10
N11C	14.50	3.11	25.52
**B vector**	**0.43**	**1.03**	**1.57**
**Θ**			**56.02°**
Pd2	22.56	4.82	19.29
N21D	20.98	5.93	18.95
**A vector**	**1.59**	**−1.11**	**0.35**
Pd3	14.93	4.14	27.10
N11D	14.94	5.90	26.15
**B vector**	**−0.01**	**−1.76**	**0.95**
**Θ**			**54.80°**

Single-walled edges

Atom/Vector	x	y	z
Pd1	25.67	9.77	29.40
N11E	26.01	10.68	27.70
**A vector**	**−0.34**	**−0.91**	**1.69**
Pd2	22.56	4.82	19.29
N21E	23.62	6.54	19.25
**B vector**	**−1.05**	**−1.72**	**0.04**
**Θ**			**59.49°**
Pd2	22.56	4.82	19.29
N21F	24.24	4.06	19.72
**A vector**	**−1.67**	**0.76**	**-0.42**
Pd4	25.22	−1.14	29.90
N11F	25.02	−1.71	27.97
**B vector**	**0.20**	**0.57**	**1.93**
**Θ**			**100.73°**
Pd3	14.93	4.14	27.10
N21G	15.40	5.18	28.80
**A vector**	**−0.48**	**−1.04**	**−1.71**
Pd1	25.67	9.77	29.40
N11G	23.88	10.27	29.23
**B vector**	**1.79**	**−0.50**	**0.17**
**Θ**			**99.29°**
Pd3	14.93	4.14	27.10
N21H	14.96	2.53	28.16
**A vector**	**−0.03**	**1.61**	**−1.06**
Pd4	25.22	−1.14	29.90
N11H	23.45	−1.88	30.16
**B vector**	**1.76**	**0.74**	**−0.27**
**Θ**			**67.72°**

Double-walled edges			
**Atom/Vector**	**x**	**y**	**z**
C23A	25.03	7.11	32.69
C38A	24.98	5.64	32.94
**A vector**	**0.05**	**1.48**	**−0.25**
C13A	25.19	1.56	33.08
C32A	25.01	3.04	33.08
**B vector**	**0.18**	**−1.49**	**0.00**
**α**			**166.91°**
C23B	28.73	7.05	29.50
C38B	28.99	5.56	29.64
**A vector**	**−0.26**	**1.49**	**−0.14**
C13B	28.60	1.41	29.81
C32B	28.86	2.90	29.69
**B vector**	**−0.26**	**−1.50**	**0.12**
**α**			**160.49°**
C23C	19.66	1.77	20.15
C38C	18.43	1.61	20.97
**A vector**	**1.24**	**0.16**	**−0.81**
C13C	15.22	1.99	23.46
C32C	16.38	1.73	22.56
**B vector**	**−1.16**	**0.26**	**0.91**
**α**			**163.44°**
C23D	18.83	6.92	19.69
C38D	17.95	7.33	20.83
**A vector**	**0.87**	**−0.41**	**−1.15**
C13D	15.69	7.28	24.24
C32D	16.52	7.47	23.02
**B vector**	**−0.84**	**−0.19**	**1.23**
**α**			**156.45°**

Single-walled edges			
**Atom/Vector**	**x**	**y**	**z**
C13E	25.93	10.87	25.29
C32E	25.50	10.29	23.98
**A vector**	**0.43**	**0.58**	**1.31**
C23E	24.71	8.40	20.44
C38E	24.91	9.19	21.72
**B vector**	**−0.20**	**−0.79**	**−1.28**
**α**			**168.03°**
C23F	25.41	2.53	21.28
C38F	25.38	1.81	22.60
**A vector**	**0.04**	**0.72**	**−1.32**
C13F	25.12	−0.95	25.63
C32F	25.28	0.10	24.59
**B vector**	**−0.16**	**−1.05**	**1.04**
**α**			**162.84°**
C23G	17.09	6.47	30.03
C38G	18.19	7.47	29.85
**A vector**	**−1.10**	**−1.00**	**0.18**
C13G	21.45	9.90	29.23
C32G	20.30	8.98	29.52
**B vector**	**1.15**	**0.91**	**−0.29**
**α**			**174.20°**
C23H	16.20	0.81	29.36
C38H	17.55	0.24	29.65
**A vector**	**−1.35**	**0.57**	**−0.29**
C13H	21.01	−1.77	30.34
C32H	19.81	−0.94	30.01
**B vector**	**1.20**	**−0.83**	**0.34**
**α**			**168.43°**

S8.6 Ligand L[1]

These measurements are taken from a previously reported single crystal structure of **L[1]**, deposited at the CCDC with number 204848.[2]

Atom/Vector	x	y	z
C3	−1.96	3.21	−1.88
C7	−0.92	2.94	−0.88
**A vector**	**−1.05**	**0.27**	**−0.99**
C3	1.96	2.21	1.88
C7	0.92	2.48	0.88
**B vector**	**1.05**	**−0.27**	**0.99**
**α**			**180.00°**

S8.7 Ligand L[2]

Atom/Vector	x	y	z
C23	−0.59	29.54	3.41
C33	0.34	28.39	3.38
**A vector**	**−0.94**	**1.15**	**0.03**
C13	3.01	25.20	3.71
C36	2.07	26.33	3.53
**B vector**	**0.94**	**−1.13**	**0.18**
**α**			**171.74°**

S8.8 Ligand L[6]

Atom/Vector	x	y	z
C14	4.53	7.43	8.51
C26	4.41	6.22	9.35
**A vector**	**0.13**	**1.21**	**−0.85**
C33	4.31	3.06	12.10
C23	4.27	4.13	11.07
**B vector**	**0.04**	**−1.07**	**1.02**
**α**			**169.15°**

S9 Analysis of Isomers of Lantern Cage 3’’

S9.1 Discussion of isomer possibilities

Lantern cage **3’’** has four ligands. The cartoon views are looking down on the structure from the top. Starting in the top left corner and numbering round anticlockwise we can define the position of each ligand in the structure (**Figure S64**).

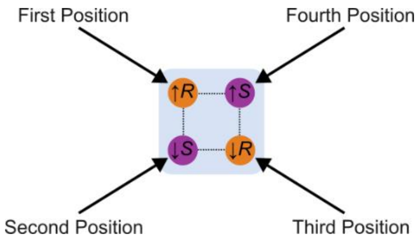

Figure S64: Defining the position of each ligand

At each ligand position, there are four possibilities when both the stereocenter and the orientation of the ligand are considered (there is a stereocenter which could be *R* or *S*, and the NH on the dihydropyridazine ring could be nearer the top (↑) or nearer the bottom (↓) when viewing the structure from the top). We have denoted these four possibilities as follows: 1 = *R*↑, 2 = *R*↓, 3 = *S*↑, 4 = *S*↓ (**Figure S65**).

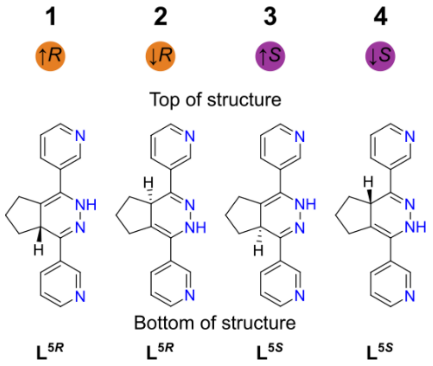

Figure S65: The four ligand possibilities

With four positions and four possibilities at each position, the total number of potential isomers is 4 × 4 × 4 × 4 = 256. Each of these 256 potential isomers can be represented as a four number code (e.g., **2342**) when looking down on the structure from the top, where the number sequence describes which possibility is present at each of the positions in turn (**Figure S66**).

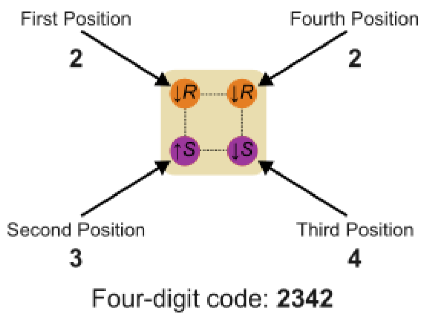

Figure S66: The four-digit code for a structure

All 256 of these four-digit numbers have been tabulated (see Section S9.2). However, many of these structures are equivalent and can be converted into each other through rotation. Where structures are equivalent, we define the structure with the lowest four-digit number as the unique structure. All unique structure numbers are shown in red, e.g., **1111**. Structure numbers which are duplicates (i.e., there is an equivalent structure with a lower four-digit number) are shown in black, e.g., **1121**.

To determine which structures are equivalent, first we consider rotation by 90°. This permutates the positions of the numbers within the four-digit sequence and can lead to up to four structures being equivalent. For example, rotation by 90° shows the following four structures as equivalent **4121** = **1214** = **2141** = **1412** (**Figure S67**). In this case **1214** is denoted the unique structure (with the lowest number) and **4121**, **2141**, and **1412** can all be discarded as duplicates.

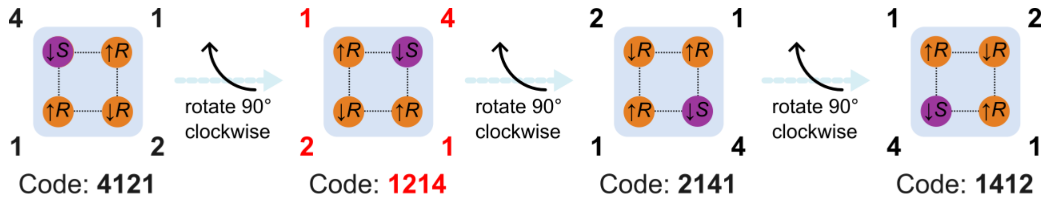

Figure S67: The equivalence of structures through 90° rotation

Secondly, we consider flipping the structure over top to bottom. We have always flipped structures by rotating by 180° around an axis running horizontally through the structure. In the flipping manoeuvre, the *R* and *S* stereocenters do not change, however the up and down arrows swap. This means that *R*↑ and *R*↓ interconvert, and *S*↑ and *S*↓ interconvert. The position of the ligands is also changed. The ligand in the first position becomes the ligand in the second position (and the ligand in the second position becomes the ligand in the first position). The ligand in the third position becomes the ligand in the fourth position (and the ligand in the fourth position becomes the ligand in the third position). In the example below (**Figure S68**), the first position ligand *R*↓ becomes *R*↑ and moves to the second position. The second position ligand *R*↓ becomes *R*↑ and moves to the first position. The third position ligand *S*↓ becomes *S*↑ and moves to the fourth position. The fourth position ligand *S*↑ becomes *S*↓

and moves to the third position. This means structure **2243** becomes the unique structure **1143**, and so structure **2243** can be discarded as a duplicate.

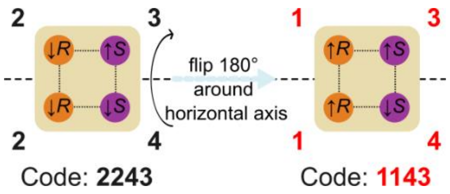

Figure S68: The equivalence of structures through flipping by 180° around horizontal axis

Finally, we consider flipping the structure over top to bottom, and then rotating the structure through sequential rotations of 90° to search for equivalent structures (**Figure S69**). The structure **2424** becomes structure **3131** upon flipping. Upon rotation structure **3131** becomes **1313**. **1313** is the unique structure which means that **2424** and **3131** can be discarded as duplicates.

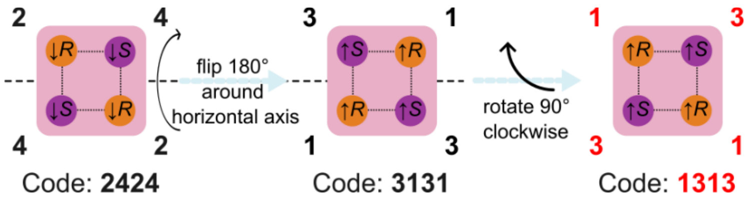

Figure S69: The equivalence of structures through flipping by 180° then rotation by 90°

Following these steps, the 256 potential isomers are reduced to a total of 39 unique structures which are isomers of each other. There are seven achiral structures and sixteen pairs of enantiomers (7 + (16 × 2) = 39). The unique structures (only one enantiomer shown of chiral structures) with their corresponding codes are shown below (**Figure S70**).

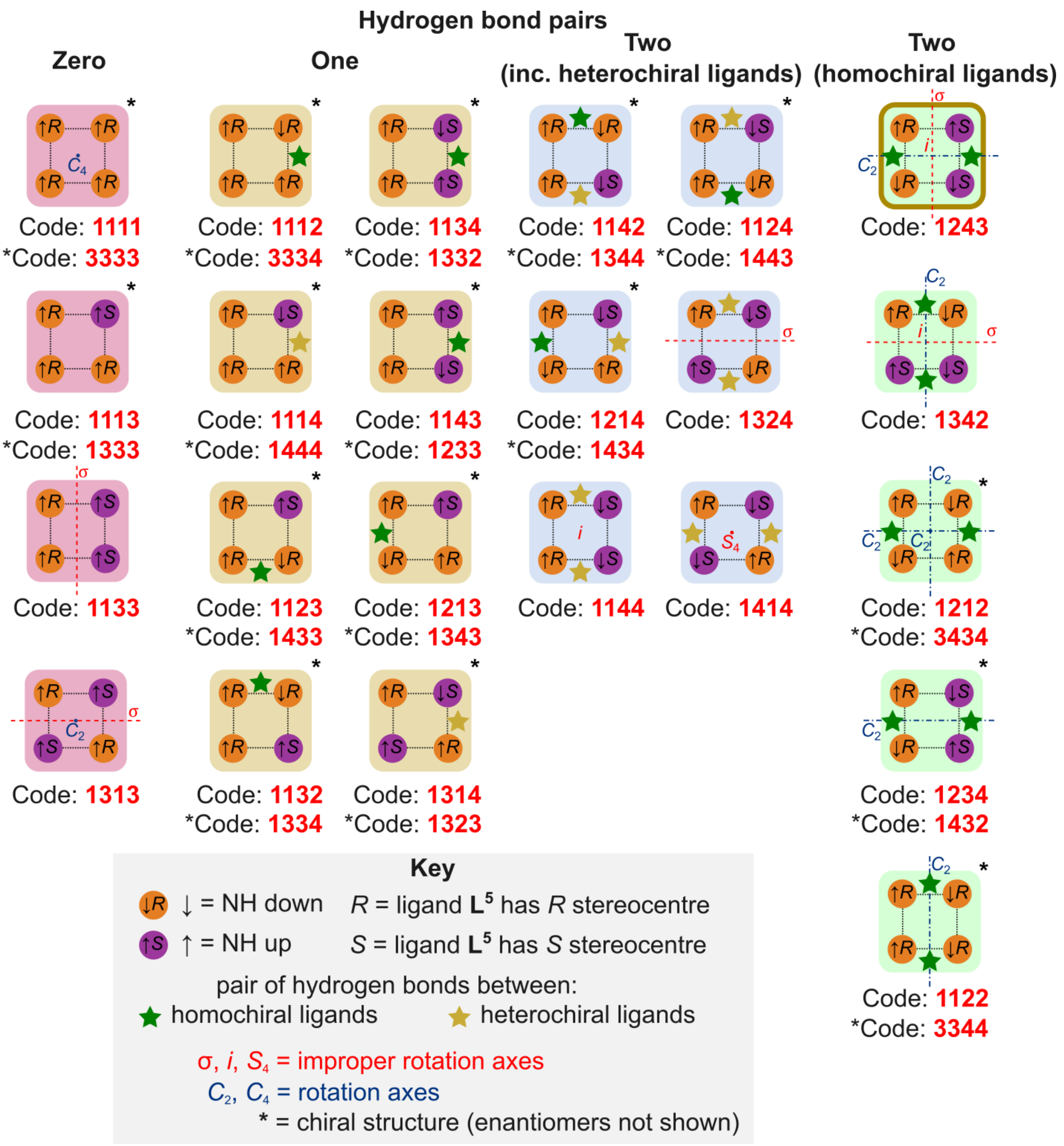

Figure S70: The unique structures with their corresponding codes

The first column in the table in Section S9.2 lists all 256 four-digit numbers in ascending order. Unique structures are shown in red, duplicates in black. If a duplicate structure can be converted into a unique structure through 90° rotation, this is shown in column two. Of the remaining duplicate structures, for those structures that can be converted into a new structure by flipping, this is shown in column three. If this flipped structure is unique then the number is shown in red. If the flipped structure is a duplicate, then this is shown in black. If flipping and then rotation is needed to convert to a unique structure, the number of the unique structure is shown in column four.

S9.2 Table showing equivalence of different structures

Structure	Converted by rotation to	Converted by flipping to	Converted by flipping and then rotation to
1111			
1112			
1113			
1114			
1121	1112		
1122			
1123			
1124			
1131	1113		
1132			
1133			
1134			
1141	1114		
1142			
1143			
1144			
1211	1112		
1212			
1213			
1214			
1221	1122		
1222		1211	1112
1223		1241	1124
1224		1231	1123
1231	1123		
1232		1214	
1233			
1234			
1241	1124		
1242		1213	
1243			
1244		1233	
1311	1113		
1312	1213		
1313			
1314			
1321	1132		
1322		4211	1142
1323			
1324			
1331	1133		
1332			
1333			
1334			
1341	1134		
1342			
1343			

1344			
1411	1114		
1412	1214		
1413	1314		
1414			
1421	1142		
1422		3211	1132
1423		3241	1324
1424		3231	1323
1431	1143		
1432			
1433			
1434			
1441	1144		
1442		3213	1332
1443			
1444			
2111	1112		
2112	1122		
2113	1132		
2114	1142		
2121	1212		
2122		2111	1112
2123		2141	1214
2124		2131	1213
2131	1213		
2132		2114	1142
2133	1332		
2134	1342		
2141	1214		
2142		2113	1132
2143	1432		
2144		2133	1332
2211	1122		
2212		1112	
2213		1142	
2214		1132	
2221		1121	1112
2222		1111	
2223		1141	1114
2224		1131	1113
2231		1124	
2232		1114	
2233		1144	
2234		1134	
2241		1123	
2242		1113	
2243		1143	
2244		1133	
2311	1123		

2312		4112	1124
2313	1323		
2314		4132	1324
2321		4121	1214
2322		4111	1114
2323		4141	1414
2324		4131	1314
2331	1233		
2332		4114	1144
2333		4144	1444
2334		4134	1344
2341	1234		
2342		4113	1134
2343		4143	1434
2344		4133	1334
2411	1124		
2412		3112	1123
2413	1324		
2414		3132	1323
2421		3121	1213
2422		3111	1113
2423		3141	1314
2424		3131	1313
2431	1243		
2432		3114	1143
2433		3144	1443
2434		3134	1343
2441		3123	1233
2442		3113	1133
2443		3143	1433
2444		3133	1333
3111	1113		
3112	1123		
3113	1133		
3114	1143		
3121	1213		
3122		2411	1124
3123	1233		
3124	1243		
3131	1313		
3132	1323		
3133	1333		
3134	1343		
3141	1314		
3142		2413	1324
3143	1433		
3144	1443		
3211	1132		
3212		1412	1214
3213	1332		

3214	1432		
3221		1421	1142
3222		1411	1114
3223		1441	1144
3224		1431	1143
3231	1323		
3232		1414	
3233		1444	
3234		1434	
3241	1324		
3242		1413	1314
3243		1443	
3244		1433	
3311	1133		
3312	1233		
3313	1333		
3314	1433		
3321	1332		
3322		4411	1144
3323		4441	1444
3324		4431	1443
3331	1333		
3332		4414	1444
3333			
3334			
3341	1334		
3342		4413	1344
3343	3334		
3344			
3411	1134		
3412	1234		
3413	1334		
3414	1434		
3421	1342		
3422		3411	1134
3423		3441	1344
3424		3431	1343
3431	1343		
3432		3414	1434
3433	3334		
3434			
3441	1344		
3442		3413	1334
3443	3344		
3444		3433	3334
4111	1114		
4112	1124		
4113	1134		
4114	1144		
4121	1214		

4122		2311	1123
4123	1234		
4124		2331	1233
4131	1314		
4132	1324		
4133	1334		
4134	1344		
4141	1414		
4142		2313	1323
4143	1434		
4144	1444		
4211	1142		
4212		1312	1213
4213	1342		
4214		1332	
4221		1321	1132
4222		1311	1113
4223		1341	1134
4224		1331	1133
4231		1324	
4232		1314	
4233		1344	
4234		1334	
4241		1323	
4242		1313	
4243		1343	
4244		1333	
4311	1143		
4312	1243		
4313	1343		
4314	1443		
4321	1432		
4322		4311	1143
4323		4341	1434
4324		4331	1433
4331	1433		
4332		4314	1443
4333	3334		
4334	3344		
4341	1434		
4342		4313	1343
4343	3434		
4344		4333	3334
4411	1144		
4412		3312	1233
4413	1344		
4414	1444		
4421		3321	1332
4422		3311	1133
4423		3341	1334

4424		3331	1333
4431	1443		
4432		3314	1433
4433	3344		
4434		3334	
4441	1444		
4442		3313	1333
4443		3343	3334
4444		3333	

S9.3 Similarity of structures 1243 and 1342

There are eight structures with two pairs of hydrogen bonds between homochiral ligands (colored with green backgrounds). We can rationalise why the three pairs of chiral structures are less favored with two other criteria. Firstly, the two pairs **1212**/**3434** and **1122**/**3344** are homochiral structures with all four ligands of the same handedness. Therefore, statistically they are disfavored due to entropy. Secondly, the two pairs **1234**/**1432** and **1122**/**3344** have lower overall symmetry, with only one C_2 axis, and are therefore less favored due to the preference to form structures of higher symmetry. This leaves the two achiral structures **1243** and **1342**, which at first glance appear very similar to each other. They both possess all the same symmetry operations and one of each of the four ligand possibilities (**Figure S71**).

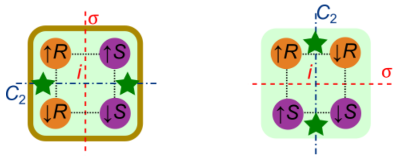

Code: **1243** Code: **1342**

Figure S71: Apparent similarity of structures **1243** and **1342**

However, these two structures are not the same. We only ever observed structure **1243** and never structure **1342** in the solid state. This difference is best visualised through comparison of the single crystal structure of **1243** and of a molecular model of **1342**. Within structure **1243**, with the ligands rotated to form the two pairs of hydrogen bonds, the hydrogens on the carbon stereocenters point towards the outside of the structure, with the hydrogens on the *R* and *S* centers on adjacent ligands pointing away from each other (**Figure S72a**). With structure **1342** with the ligands rotated to form the two pairs of hydrogen bonds, the hydrogens on the carbon stereocenters point towards the inside of the structure, with the hydrogens on the *R* and *S* centers on adjacent ligands pointing towards each other (**Figure S72b**). If structure **1342** is forced to adopt a conformation where the hydrogens on the carbon stereocenters point towards the outside of the structure, with the hydrogens on the *R* and *S* centers on adjacent ligands pointing away from each other (**Figure S72c**), then the NH groups are in the incorrect orientation (head-to-head) for hydrogen bonding to occur. This highlights that these structures

are not in fact equivalent and are different in energy, with the hydrogens pointing towards the out of the structure clearly being favored.

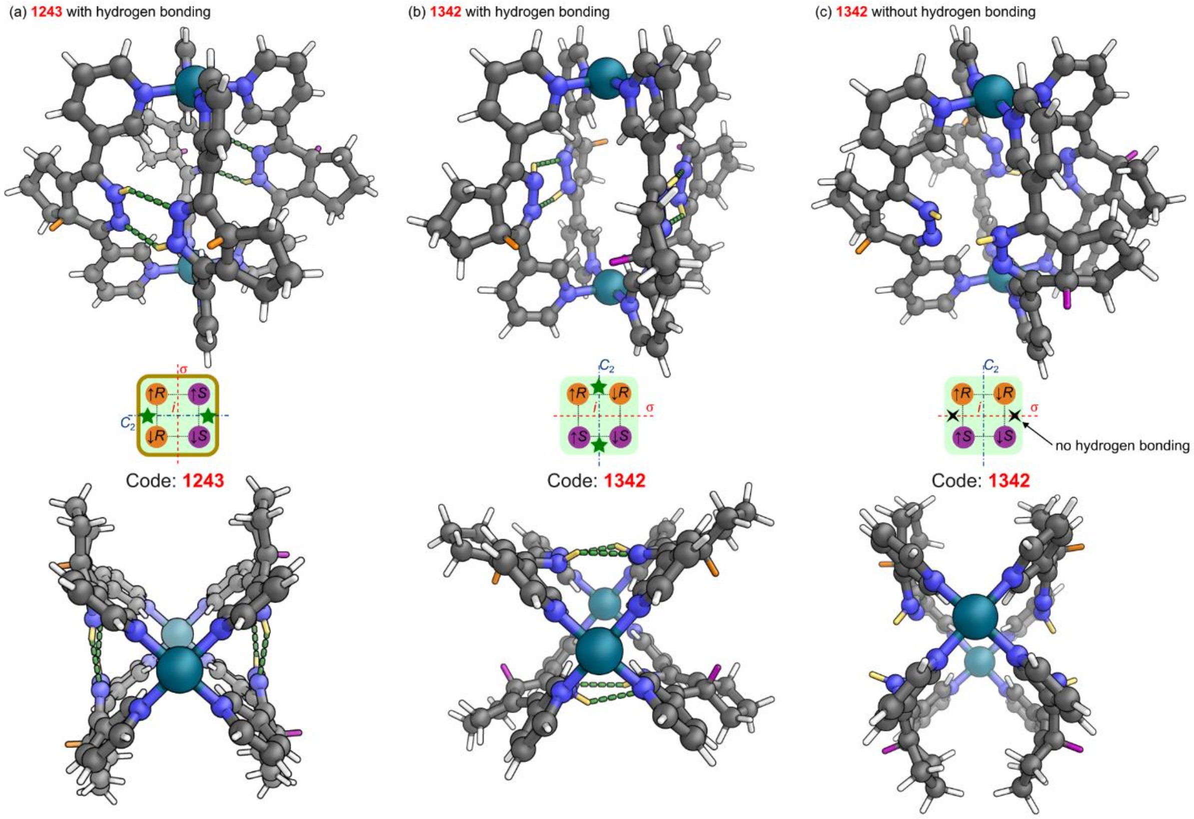

Figure S72: Different orientation of substituent groups in structures **1243** and **1342** (a) Observed structure **1243** with hydrogen bonding; (b) Structure **1342** (not observed) showing different orientation of groups required to get hydrogen bonding; (c) Structure **1342** (not observed) forced to be in the same orientation as the observed **1243** structure, showing that hydrogen bonding is not possible in this orientation.

The theoretical calculations to generate the structures for **1342** were performed using the Gaussian 09 package.[3] **1342** with HB and **1342** without HB were optimized using PM6 semi-empirical methods and their energy minimized structures were obtained. No symmetry constraints were used during the optimization procedure.

S9.4 Comparison to bullvalene system

The combination of structural features in a lantern cage observed in this work is rare. To the best of our knowledge, the single prior paper which described the same stereochemical features is that of Bloch *et. al.* and involves a lantern made from bullvalene ligands.[4]

In the bullvalene case, there is a head to tail difference in the ligand and the ligand also exists as a pair of enantiomers. This is referred to with *M* and *P* helicity rather than *R* and *S* by the authors. This combination of features means the stereochemical problem is the same (four ligand possibilities and four positions). We thank the authors of this paper for their efforts to

explain the isomer possibilities in this system, and we have followed their numbering convention for discussions.

In the bullvalene paper, the authors report 38 unique isomers (15 enantiomer pairs and 8 achiral isomers). Whilst we came to a very similar conclusion, we believe there are some errors in the list presented in the bullvalene paper (highlighting the complex nature of this stereochemical problem). Accounting for these presumed errors (two duplicates; three omissions, some incorrect determinations of achiral/chiral and matching of enantiomers), our stereochemical analysis is the same. These are listed below for reference.

(a) Structure 1423 is not unique and is the same as structure 1324 (through a flip by 180° around a horizontal axis and then rotation by 90°).

(b) Structure 1244 is not unique and is the same as 1233 (through a flip by 180° around a horizontal axis).

(c) Structure 1234 is chiral rather than achiral. This structure only possesses a C_2 axis which does not make it achiral. The enantiomeric structure 1432 has been omitted.

(d) The achiral structure 1342 is missing.

(e) The chiral structure 1332 is missing.

(f) Structures 1123 and 1433 are enantiomers, and structures 1132 and 1334 are enantiomers. These have been incorrectly paired (as 1123 with 1334, and 1132 with 1433).

(g) Structure 1134 is not the enantiomer of 1233 as listed. Structure 1134 is the enantiomer of structure 1332 which was missing.

(h) Structure 1143 is the enantiomer of structure 1233.

S9.5 Consideration of hydrogen bonds

In addition to the possibilities of isomerism already listed, the 39 isomers would split into more possibilities if differing positions of the hydrogen bonds also led to additional isomers. However, we have not considered the different position/orientation of the hydrogen bonds as leading to different structures. In the same way that a distinction is made between configurational isomers (which are not interconvertible at room temperature) and conformational isomers which are, the energy barrier of interconversion should be considered. Hydrogen bonds are known to be dynamic. Interconversion between structures with different hydrogen bonding patterns could be achieved by rotation around the single bonds at the 3 and 6-positions of the dihydropyridazine rings. The energetic barrier for this rotation would only

require the breaking of at most two hydrogen bonds and rotation around single bonds. We believe that there should be sufficient energy for this to be dynamic at room temperature. Hence, where structures have the possibility of more than one arrangement with the same number of hydrogen bonds, or arrangements with different numbers of hydrogen bonds (for example the two orientations of structure **1342** in **Figure S72**), we believe these should be considered as different conformers rather than different stereochemical isomers. We contrast this to any possible equilibration between the 39 structures already mentioned which requires the making/breaking of covalent bonds (either through tautorimerization of the ligand and/or metal de-ligation and re-ligation). The energy barrier for this would be considerably higher.

S9.6 Additional possibilities of isomerism in lantern 3

In addition to the stereocenter on the dihydropyridazine ring in lantern **3**, additional isomeric possibilities are generated from the groups added from the norbornene. The hydrogen on the dihydropyridazine ring can either be *cis* to the one carbon bridge or *cis* to the two carbon bridge. In the single crystal structure of lantern **3**, the solution is most consistent with the one carbon bridges of the structure pointing towards each other across pairs of hydrogen bonded ligands (**Figure S63**). It is easy to rationalize why this may be the preferred isomer due to a smaller steric clash between the one carbon bridges of paired ligands. However, analysis of the data shows that the overall solution may be a mixture. The orientation of the dihydropyridazine rings and the average distances between the nitrogen atoms of 3.19 Å is strongly suggestive of pairs of hydrogen bonds and ligands orientated in a head-to-tail arrangement, even though hybridization of individual atoms on the ring cannot be determined.

Hence, as the data do not conclusively support the sole existence of a single isomer in the solid state, we have not analysed the total number of isomers here. It is worth emphasising that whereas the 39 isomers of lantern cage **3’’** detailed above can interconvert solely by tautomerization, lantern **3** contains additional stereocenters on the norbornyl bridges that are not tautomerizable. The interconversion between isomers which have bridges orientated in different directions (towards each other or away from other) requires metal de-ligation and re-ligation and so may have a higher energy barrier than interconversion mechanisms relying solely on tautomerization.

S10 X-ray Crystallography

Single crystals were selected and mounted using Fomblin® (YR-1800 perfluoropolyether oil) on a polymer-tipped MiTeGen MicroMountTM and cooled rapidly to 120 K in a stream of cold N_2 using an Oxford Cryosystems open flow cryostat.[5] Single crystal X-ray diffraction data were collected on an XtaLAB PRO MM007 (PILATUS3 R 200K Hybrid Pixel Array detector, mirror-monochromated Cu-K_α radiation source; λ = 1.54184 Å, ω scans). Cell parameters were refined from the observed positions of all strong reflections and absorption corrections were applied using a Gaussian numerical method with beam profile correction (CrysAlisPro).[6] Structures were solved within Olex2[7] by dual space iterative methods (SHELXT)[8] and all non-hydrogen atoms refined by full-matrix least-squares on all unique F^2 values with anisotropic displacement parameters (SHELXL).[9] Hydrogen atoms were refined with constrained riding geometries and thermal parameters linked to U_{iso} of their parent atoms. Hydrogen atoms were refined both freely (see specific crystal structure and refinement details section for each crystal) and with constrained riding geometries and thermal parameters linked to U_{iso} their parent atoms. Structures were checked with checkCIF.[10] CCDC 2314587, 2314588, 2314589, 2314590, 2314591, 2314592, and 2314593 contain the supplementary data for these compounds. These data can be obtained free of charge from The Cambridge Crystallographic Data Centre via www.ccdc.cam.ac.uk/data_request/cif.

X-ray diffraction measurements were performed in Experiments Hutch 1 (EH1) of Beamline I19, at Diamond Light Source.[11] The data were collected at a wavelength of 0.6889 Å on a Fluid Film Devices 3-circle fixed-chi diffractometer using a Dectris Pilatus 2M detector. The crystal was mounted on a MiTeGen MicroMountTM using a perfluoropolyether oil and cooled for data collection by a Cryostream nitrogen gas stream.[4] The collected frames were integrated using XIA26 software[11c] and the data were corrected for absorption effects using AIMLESS,[12] an empirical method.

Table 1. Experimental details for ligand L^2 and L^6, cages 1 and 2.

	**L^2**	**L^6**	Tetrahedral cage **1**	Tetrahedral cage **2**
Chemical formula	$C_{14}H_{10}N_4$	$C_{17}H_{14}N_4·0.5(OH_2)$	$C_{95.976}H_{64}N_{47.953}Pd_4·2.298(C_2H_3N)·C_2N·7.4(BF_4)·0.6[BF_4]·15[C_2H_3N]$	$C_{112}H_{80}N_{32}Pd_4·6.589(BF_4)·C_2H_3N·1.437(C_2N)·1.4[BF_4]·18[C_2H_3N]$
Sum formula	$C_{14}H_{10}N_4$	C_{17} H_{15} N_4 $O_{0.50}$	$C_{132.57}$ $H_{115.89}$ B_8 F_{32} $N_{66.25}$ Pd_4	$C_{152.87}$ H_{137} $B_{7.99}$ $F_{31.96}$ $N_{52.44}$ Pd_4
M_r	234.26	283.33	3757.23	3827.89
Crystal system, space group	Monoclinic, $P2_1/c$	Orthorhombic, *Pbcn*	Triclinic, *P*	Triclinic, $P\overline{1}$
Temperature / K	120	120	100	120
a, *b*, *c* / Å	4.5796 (2), 43.8136 (9), 5.7594 (2)	7.2789 (3), 16.1168 (5), 23.0135 (8)	19.35400 (8), 22.4899 (1), 22.60230 (9)	19.3508 (3), 22.5049 (3), 22.6197 (4)
α, β, γ / °	108.538 (3)	90	77.7350 (4), 70.5830 (4), 77.8840 (4)	77.919 (1), 70.658 (2), 77.806 (1)
V / $Å^3$	1095.65 (7)	2699.77 (17)	8962.64 (7)	8980.6 (3)
Z	4	8	2	2
Radiation type	Cu K_a	Cu K_a	Synchrotron, λ = 0.6889 Å	Cu K_a
μ / mm^{-1}	0.72	0.71	0.46	4.01
Crystal size / mm	0.25 × 0.10 × 0.07	0.05 × 0.03 × 0.02	0.1 × 0.06 × 0.02	0.42 × 0.18 × 0.07
Diffractometer	SuperNova, Atlas S2	XtalLAB PRO *MM007*, PILATUS3 R 200K	I19 Experimental Hutch 1 Fluid Film Devices	SuperNova, Titan S2
T_{min}, T_{max}	0.801, 1.000	0.960, 1.000	0.973, 1.0	0.304, 1.000
No. of measured, independent and observed [$I > 2\sigma(I)$] reflections	14718, 2185, 2022	9873, 2696, 2064	150518, 53622, 38966	142427, 35003, 26703
R_{int}	0.029	0.042	0.035	0.090
$(\sin \theta/\lambda)_{max}$ / $Å^{-1}$	0.619	0.628	0.714	0.621
$R[F^2 > 2s(F^2)]$, $wR(F^2)$, S	0.048, 0.126, 1.11	0.063, 0.190, 1.09	0.065, 0.231, 1.08	0.079, 0.255, 1.08
No. of reflections	2185	2696	53622	35003
No. of parameters	163	200	2003	1722
No. of restraints	0	6	9036	6528
$\Delta\rho_{max}$, $\Delta\rho_{min}$ / e $Å^{-3}$	0.20, -0.20	0.99, -0.23	3.18, -1.52	2.12, -1.33

Table 2. Experimental details for cages 3,3'', 3''T, and 4

	Lantern cage **3**	Lantern cage **3''**	Tetrahedral cage **3''T**	Tetrahedral cage **4**
Chemical formula	$C_{60.191}H_{49.948}N_{12.487}Pd_2{\cdot}1.757(C_4N_2){\cdot}1[C_2H_3N]{\cdot}4[BF_4]{\cdot}4[C_2H_3N]$	$2(C_{68}H_{64}N_{16}Pd_2){\cdot}4(C_2H_3N){\cdot}6(BF_4){\cdot}2[C_2H_3N]{\cdot}2[BF_4]{\cdot}2[C_2H_3N]$	$C_{136}H_{120}N_{32}Pd_4{\cdot}2.75(BF_4){\cdot}5.25[(BF_4)]{\cdot}8[C_2H_3N]$	$C_{136}H_{112}N_{32}Pd_4{\cdot}4(BF_4){\cdot}$[+solvents]
Sum formula	$C_{86}\ H_{87}\ B_4\ F_{16}\ N_{21}\ Pd_2$	$C_{152}\ H_{152}\ B_8\ F_{32}\ N_{40}\ Pd_4$	$C_{152}\ H_{144}\ B_8\ F_{32}\ N_{40}\ Pd_4$	$C_{156}\ H_{142}\ B_8\ F_{32}\ N_{42}\ Pd_4$
M_r	1974.80	3659.21	3651.14	3725.19
Crystal system, space group	Monoclinic, *I*2/*m*	Triclinic, $P\overline{1}$	Monoclinic, $P2_1/n$	Monoclinic, *C*2/*c*
Temperature / K	100	120	120	100
a, *b*, *c* / Å	13.6862 (6), 13.8329 (6), 24.7010 (13)	15.2333 (6), 15.9543 (7), 17.5903 (3)	20.3860 (6), 26.5231 (6), 34.0785 (10)	40.079 (5), 17.9825 (15), 45.019 (5)
α, β, γ / °	90.890 (4)	103.818 (3), 96.828 (2), 114.595 (4)	102.204 (3)	95.030 (12)
V / $Å^3$	4675.8 (4)	3657.5 (3)	18009.8 (9)	32321 (4)
Z	2	1	4	8
Radiation type	Synchrotron, l = 0.6889 Å	Cu K_a	Cu K_a	Synchrotron, l = 0.6889 Å
μ / mm^{-1}	0.44	4.86	3.95	0.50
Crystal size / mm	0.05 × 0.05 × 0.04	0.09 × 0.05 × 0.02	0.13 × 0.11 × 0.06	0.05 × 0.02 × 0.02
Diffractometer	I19 Experimental Hutch 1 Fluid Film Devices	XtalLAB PRO *MM007*, PILATUS3 R 200K	XtalLAB PRO *MM007*, PILATUS3 R 200K	I19 Experimental Hutch 1 Fluid Film Devices
T_{min}, T_{max}	0.437, 1.000	0.791, 0.957	0.742, 0.951	0.998, 1.0
No. of measured, independent and observed [$I > 2\sigma(I)$] reflections	24753, 3512, 2657	83715, 14745, 9100	222941, 23750, 10494	83558, 9770, 4436
R_{int}	0.059	0.109	0.099	0.258
$(\sin \theta/\lambda)_{max}$ / $Å^{-1}$	0.555	0.630	0.542	0.417
$R[F^2 > 2s(F^2)]$, $wR(F^2)$, S	0.113, 0.354, 1.51	0.084, 0.271, 1.08	0.157, 0.486, 1.57	0.175, 0.471, 1.40
No. of reflections	3512	14745	23750	9770
No. of parameters	269	988	741	836
No. of restraints	681	2088	2107	4129
$\Delta\rho_{max}$, $\Delta\rho_{min}$ / e $Å^{-3}$	1.99, -0.47	2.10, -1.98	2.36, -1.02	2.13, -0.74

S10.1 Single Crystal Structure of L²

Single crystals of **L²** ($C_{14}H_{10}N_4$) were prepared via slow evaporation from MeCN. A suitable crystal was selected and mounted using FomblinÂ. (YR-1800 perfluoropolyether oil) on a SuperNova, Atlas S2 diffractometer. The crystal was kept at 120(2) K during data collection. Using Olex2[7] the structure was solved with the SHELXT[8] structure solution program using Intrinsic Phasing and refined with the SHELXL[9] refinement package using Least Squares minimisation.

S10.1.1 Specific crystal structure and refinement details for L²

All hydrogen atoms were observed in the electron density map before being geometrically placed and refined using a riding model.

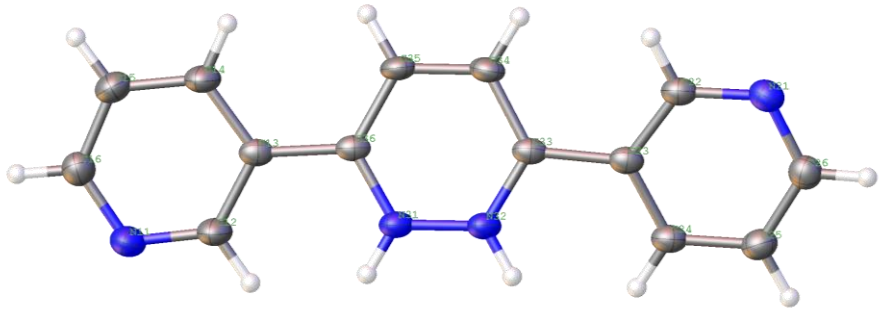

Figure S73: Image of **L²** at 50% ellipsoids

S10.2 Single Crystal Structure of L^6

Single crystals of **L⁶** ($C_{17}H_{15}N_4O_{0.5}$) were prepared via slow evaporation from MeCN. A suitable crystal was selected and mounted using FomblinÂ. (YR-1800 perfluoropolyether oil) on a polymer-tipped MiTeGen MicroMountTM and cooled rapidly to 120 K in a stream of cold N_2 using an Oxford Cryosystems open flow cryostat on a XtalLAB PRO MM007, PILATUS3 R 200K diffractometer. The crystal was kept at 120(2) K during data collection. Using Olex2,[7] the structure was solved with the SHELXT[8] structure solution program using Intrinsic Phasing and refined with the SHELXL[9] refinement package using Least Squares minimisation.

S10.2.1 Specific crystal structure and refinement details for L^6

CheckCIF gives 3 B level alerts. These alerts result from a disordered water molecule. Water residue O2 is disordered over a two-fold rotation symmetry element with each site refined at half occupancy and placed into part −1. A sensible model for the hydrogen atoms of the water residues could not be developed despite the presence of residual electron density in the Fourier map, possibly indicative of further unmodelled disorder. The hydrogen atoms for the water residue were not included in the model, however, they are included in the unit cell contents and derived parameters. The anisotropic displacement parameters of the water oxygen atom were restrained to have more isotropic character (ISOR).

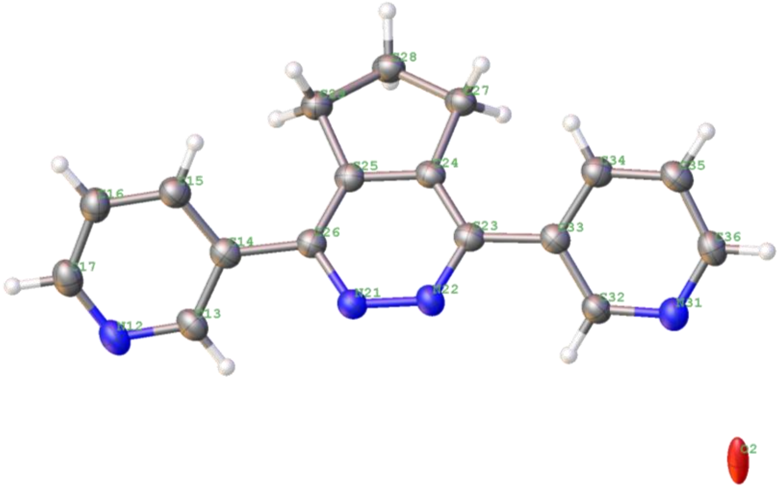

Figure S74: Image of **L⁶** at 50% ellipsoids

S10.3 Single Crystal Structure of tetrahedral cage 1

Single crystals of tetrahedral cage **1** ($C_{132.57}H_{115.89}B_8F_{32}N_{66.25}Pd_4$) were grown via slow evaporation of diethyl ether into MeCN. A suitable crystal was selected and mounted in fomblin film on a micromount on a Fluid Film Devices diffractometer. The crystal was kept at 100(2) K during data collection. Using Olex2,[7] the structure was solved with the SHELXT[8] structure solution program using Intrinsic Phasing and refined with the SHELXL[9] refinement package using Least Squares minimisation.

S10.3.1 Specific crystal structure and refinement details for tetrahedral cage 1

Diffraction data for the large supramolecular coordination cage complex was somewhat weak at high angle despite the use of synchrotron radiation. The data used in the refinement was truncated to a resolution of 0.7 Å at which point the Isigma value for the data dropped below 3. The porous nature of the cage structure containing and surrounded by diffuse and mobile anions and solvent residues will have contributed to the weak high angle data. Rigid bond and similarity restraints were applied to all isotropic and anisotropic displacement parameters of carbon, nitrogen, boron, and fluorine atoms in the structure (RIGU, SIMU). Geometric similarity restraints were applied to all symmetrically related ligand moieties (including disorder components), tetrafluoroborate anions, and solvent residues (SADI, SAME). The disordered tetrazine ring moieties were restrained to have planar geometry (FLAT). Tetrafluoroborate anion B91 was refined with rigid geometry with coordinates taken from the Olex2 FragmentDB. The displacement parameters of tetrafluoroborate anion B91 were refined isotropically with the value for all atoms constrained to have an identical value. The occupancies of tetrafluoroborate anions B21, B71, B81, and B91 were initially refined before being fixed at indicated values of 0.7, 0.8, 0.6, and 0.3 respectively. The tetrazine ring moieties of ligands A, C, D, F, and G are disordered over two conformations, the occupancies of which have been refined and the disordered component pairs constrained to sum to unity. The anisotropic displacement parameters of closely lying disorder counterpart atoms were constrained to have identical values (EADP). The occupancy of acetonitrile residue N11 was refined to a value of 0.81(1) and the occupancy of acetonitrile residue N41 was fixed at a value of 0.5. The hydrogen atoms of acetonitrile residue N31 could not be converged and were not included in the model. A solvent mask was calculated, and 748 electrons were found in a volume of 3079 $Å^3$ in 1 void per unit cell. This is consistent with the presence of 0.6[BF_4], 15[C_2H_3N] per Unit Cell which account for 709 electrons per unit cell.

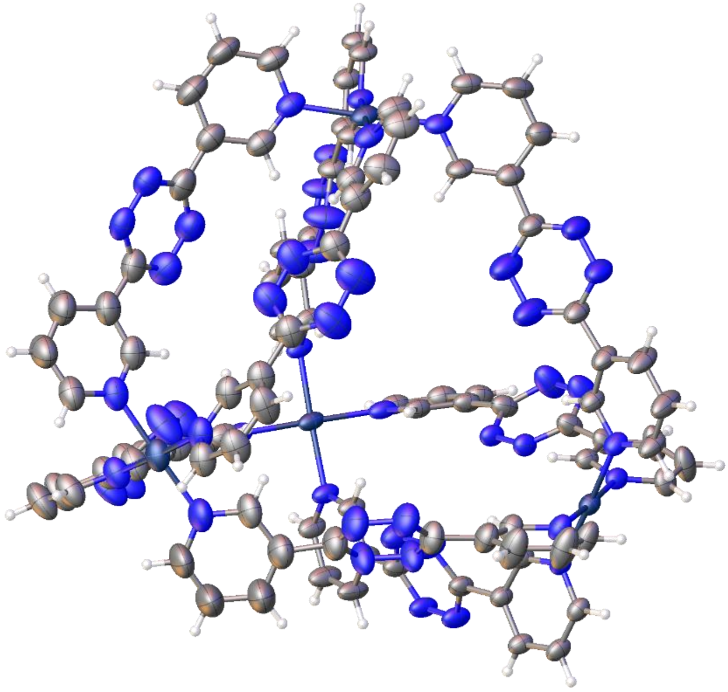

Figure S75: Image of tetrahedral cage **1** at 50% ellipsoids

S10.4 Single Crystal Structure of tetrahedral cage 2

Single crystals of tetrahedral cage **2** ($C_{152.87}H_{137}B_{7.99}F_{31.96}N_{52.44}Pd_4$) were grown via slow evaporation of diisopropyl ether into MeCN. A suitable crystal was selected and mounted in fomblin film on a micromount on a SuperNova, Titan S2 diffractometer. The crystal was kept at 120(2) K during data collection. Using Olex2,[7] the structure was solved with the SHELXT[8] structure solution program using Intrinsic Phasing and refined with the SHELXL[9] refinement package using Least Squares minimisation.

S10.4.1 Specific crystal structure and refinement details for tetrahedral cage 2

The crystal of the large metallo-coordination cage was moderately diffracting with weak data at high angle as a consequence of large regions of diffuse anions and solvents between the cage residues. Restraints were used wherever possible to aid refinement of the large structure. Rigid bond and similarity restraints were applied to the anisotropic displacement parameters of all atoms in the structure (SIMU, RIGU).

Geometric similarity restraints were applied to the 1,2 and 1,3 bond distances of chemically identical moieties across the eight symmetrical coordinated ligands and tetrafluoroborate anions (SAME, SADI). Geometric restraints were applied to the 1,2 and 1,3 distances of acetonitrile solvent residues T and S (DFIX), whilst acetonitrile solvent residue U was refined as a rigid body using coordinates from the Olex2 FragmentDB. The pyridazine ring moieties of ligands B, D, and G are disordered over two conformations which each share atoms sites for the substituted carbon atoms. The occupancies of the disorder components of ligand moieties D and G were fixed at 50% each, whilst the occupancies for moiety B were refined and constrained to sum to unity resulting in values of 0.71(1) and 0.29(1).

The orientation of the pyridazine rings and hence identity of the carbon and nitrogen atom sites was made by examination of the bond lengths around the six-membered rings and the curvature of the ligands which have a crescent shape with the N=N moiety on the inside of the curve. Assignment of the carbon and nitrogen atoms of these moieties could not be made on the basis of displacement parameters or hydrogen atom sites owing to the noisy electron density map. It cannot be ruled out that some of the time the pyridazine rings are disordered with their two orientations superimposed on each other.

The occupancies of tetrafluoroborate anions B11, B21, B31, B41, B51, B71, and B91 were allowed to refine using free variables whilst anions B61 and B81 were refined at full occupancy. Tetrafluoroborate anions B61, B81, and B91 were refined with anisotropic atomic displacement parameters whilst the remaining anions could only be refined with isotropic atomic displacement parameters. The isotropic atomic displacement parameters of anions

B11, B41, and B71 were fixed at a value of 0.2. A total of eight tetrafluoroborate anions are expected in the structure to balance the charge of the four Pd(II) cations in the cage complex. A total of occupancy of 6.4 tetrafluoroborate anions is modelled across nine sites, the remainder of the charge balancing anions are likely to be disordered across otherwise partially occupied sites and in diffuse void regions treated with a solvent mask. The atomic displacement parameters of the acetonitrile solvent residues S and T were refined with isotropic atomic displacement parameters, whilst the isotropic values for residue U were fixed at values of 0.2. The occupancies of solvent residues T and U were refined with free variables.

A residual electron density peak of 1.35 e $Å^{-3}$ is observed 0.762 Å from Pd1. This peak is not consistent with any unmodelled disorder and is likely an artefact of deficiencies in the adsorption correction.

A solvent mask was calculated, and 836 electrons were found in a volume of 3202 $Å^3$ in 1 void per unit cell. This is consistent with the presence of 1.4[BF_4], 18[C_2H_3N] per asymmetric unit which accounts for 907 electrons per unit cell.

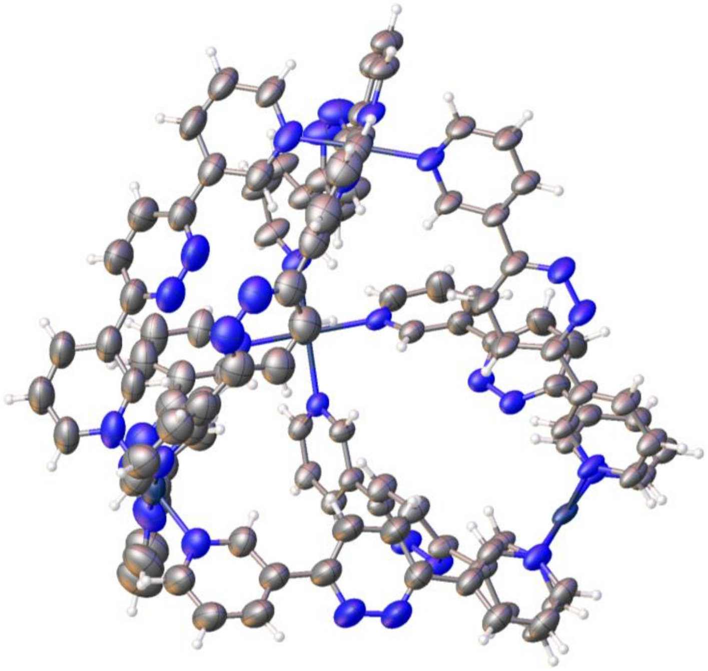

Figure S76: Image of tetrahedral cage **2** at 50% ellipsoids

S10.5 Single Crystal Structure of 3

Single crystals of lantern cage **3** ($C_{86}H_{87}B_4F_{16}N_{21}Pd_2$) were grown via vapour diffusion of diisopropyl ether into MeCN while in a dark environment. A suitable crystal was selected and mounted in fomblin film on a micromount on a Fluid Film Devices diffractometer. The crystal was kept at 100(2) K during data collection. Using Olex2,[7] the structure was solved with the SHELXT[8] structure solution program using Intrinsic Phasing and refined with the SHELXL[9] refinement package using Least Squares minimisation.

S10.5.1 Specific crystal structure and refinement details for lantern cage 3

Crystals of the large solvated supramolecular cage complex diffracted moderately strongly to a resolution of 0.9 Å; a synchrotron radiation source was used, and several crystals were run to obtain the best dataset. Refinement of the structure is complicated by the presence of several modes of disorder that are possible for the conformation and orientation of the dihydropyridazine-norbornyl moiety. Rigid bond and similarity restraints were applied to all isotropic and anisotropic displacement parameters of carbon and nitrogen atoms in the structure (RIGU, SIMU).

It was not possible to distinguish the orientation of the dihydropyridazine amine and imine nitrogen atoms from the electron density map despite the expectation this assignment could be made based on bond lengths, bond angles, and the presence of peaks for hydrogen atoms. A conformational disorder component is modelled for the dihydropyridazine ring rotated approximately 90 degrees along the pyridyl-pyridyl axis. The occupancies of these disorder components were refined and constrained to sum to unity, giving a value of 0.56(1) for the major component. The associated norbornyl moiety for the minor dihydropyridazine component could not be distinguished in the electron density map and was not included in the model. The position of the minor dihydropyridazine disorder component with respect to the pyridyl-pyridyl axis indicated that there may also be some related disorder in the pyridyl rings, however, a model accounting for this disorder could not be developed.

Extensive use of geometric restraints has been used to aid refinement of the model. The geometries of the pyridyl and norbornyl moieties have been restrained to have target 1,2 and 1,3 bond distances with the target values taken from the fragment DB library in Olex2 (DFIX, DANG). Geometric similarity restraints have been applied to the parts of the ligand expected to have precisely (or approximately in the case of some disordered moieties) geometries (SADI). Given that it was not possible to distinguish the specific orientation of the C1-symmetric dihydropyridazine ring, but it was necessary to restrain its geometry to sensible values, 1,2 and 1,3 distance restraints of 1.35 and 2.34 Å were applied to all the bond lengths

and angles round the ring (DFIX, DANG), which was additionally restrained to have approximately planar geometry (FLAT). These restraint values represent averages of the longer and shorter bonds that would be expected to be observed around the ring if only one orientational component were present.

A solvent mask was calculated, and 556 electrons were found in a volume of 2022 Å^3 in 2 voids per unit cell. This is consistent with the presence of 1[C_2H_3N], 4[BF_4], 4[C_2H_3N] per formula unit which account for 548 electrons per unit cell. The assignment of the masked residues was made based on charge balance, presence of cavity bound solvent in other structures of similar cages and adherence to plausible overall molecular volumes.

Conclusions that can be drawn from this structure are the topology and composition of the [Pd_2L_4]$^{4+}$ lantern complex cation and the intramolecular conformation of the dihydropyridazine-norbornyl moieties. From this intramolecular conformation of the dihydropyridazine rings of adjacent ligands it is possible to determine that they are oriented such that the amine and imine groups are able and likely to be donating and accepting a pair of hydrogen bonds between each pair of ligands, as has been observed in similar structures.

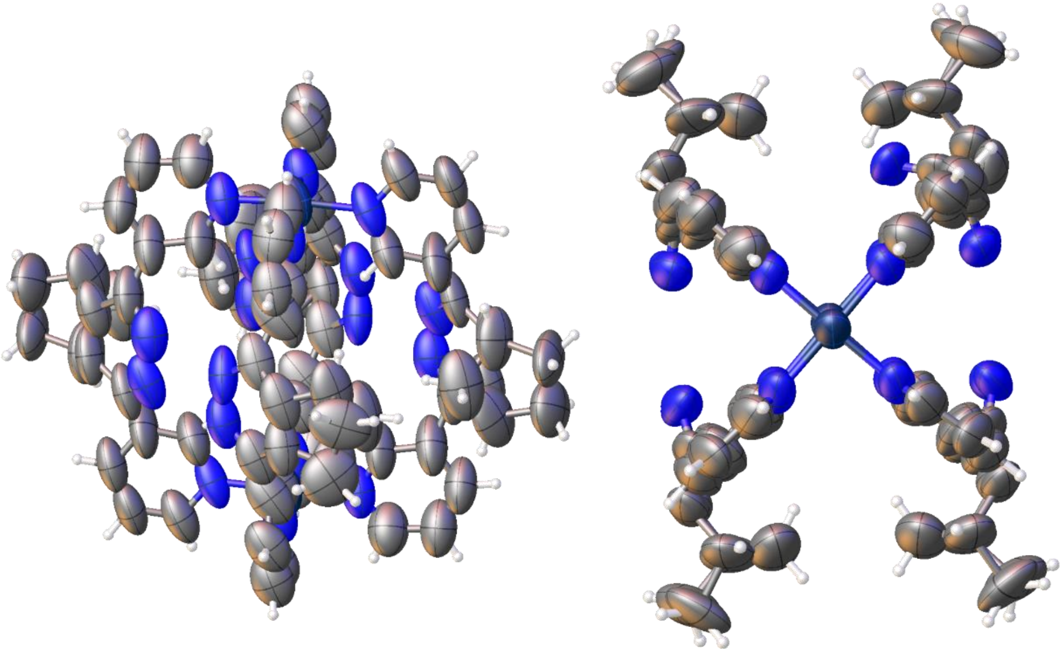

Figure S77: Image of lantern cage **3** at 50% ellipsoids

S10.6 Single Crystal Structure of 3’’

Single crystals of lantern cage **3’’** ($C_{152}H_{152}B_8F_{32}N_{40}Pd_4$) were grown via vapour diffusion of diisopropyl ether into MeCN while in a dark environment. A suitable crystal was selected and mounted in fomblin film on a XtalLAB PRO MM007, PILATUS3 R 200K diffractometer. The crystal was kept at 120(2) K during data collection. Using Olex2,[7] the structure was solved with the SHELXT[8] structure solution program using Intrinsic Phasing and refined with the SHELXL[9] refinement package using Least Squares minimisation.

S10.6.1 Specific crystal structure and refinement details for lantern cage 3’’

The crystal of a minority morphology diffracted weakly. No other crystal of this phase could be found in this or subsequent batches of crystals. Restraints were applied to the geometry and anisotropic displacement parameters of the structure to aid refinement and support the low data to parameter ratio. Many attempts were made to grow stronger diffracting crystals. Restraints were used wherever possible to aid refinement of the large structure. Rigid bond and similarity restraints were applied to the anisotropic displacement parameters of all atoms in the structure except Pd (SIMU, RIGU). The C–C bond lengths of the pyridine-dihydropyridazine bridging atoms are restrained to be similar (SADI). Geometric similarity restraints were applied to the 1,2 and 1,3 bond distances of chemically identical pyridine moieties across the four symmetrical coordinated ligands (SAME). Tetrafluoroborate anions B11E and B11G were refined at full occupancy whilst B1F and B1F' were allowed to refine with free variables. The anisotropic displacement parameters of pairs of disordered tetrafluoroborate borons B1F and B1F' were constrained to be identical. A total of four tetrafluoroborate anions are expected in the structure to balance the charge of the two Pd(II) cations in the cage complex. A total occupancy of three tetrafluoroborate anions is modelled across three sites. The remainder of the charge balancing anions are likely to be disordered across otherwise partially occupied sites and in diffuse void regions treated with a solvent mask. Disordered solvent molecules and a tetrafluoroborate anion could not be sensibly modelled, so the structure was treated with a solvent mask. The occupancies of solvent residues T and S were refined at full occupancy. A solvent mask was calculated, and 100 electrons were found in a volume of 287 $Å^3$ in 3 voids per unit cell. This is consistent with the presence of 2[C_2H_3N], 2[BF_4], 2[C_2H_3N] per unit cell which accounts for 170 electrons per unit cell. Each cage in the unit cell appears to contain an acetonitrile which is four-fold disordered and could not be sufficiently modelled. All hydrogen atoms were geometrically placed and refined using a riding model except hydrogens H37A, H37B, H37C, and H37D which were found based upon the sp^3 geometry of the carbon on the dihydropyridazine (AFIX 137). Hydrogen atoms H37C and H37D could be seen in the electron density map supporting the

placement based on the geometry of the carbon. Hydrogen atoms H31D and H31C could be seen in the electron density map which support the presence of hydrogen bonding. Pairs of large residual electron density peaks in the range 2.088-2.250 e Å^{-3} are located on opposite sides of the palladium atoms of both complexes (Pd-Q peak distances range 0.873-0.924 Å). This peak is not consistent with any unmodelled disorder and is likely an artefact of deficiencies in the adsorption correction.

Table 3. Selected hydrogen-bond parameters

D—H···*A*	*D*—H / Å	H···*A* / Å	*D*···*A* / Å	*D*—H···*A* / °
N31A—H31A···N39B	0.88	2.25	2.977 (9)	140.1
N31D—H31D···N39C	0.88	2.22	2.932 (9)	137.8

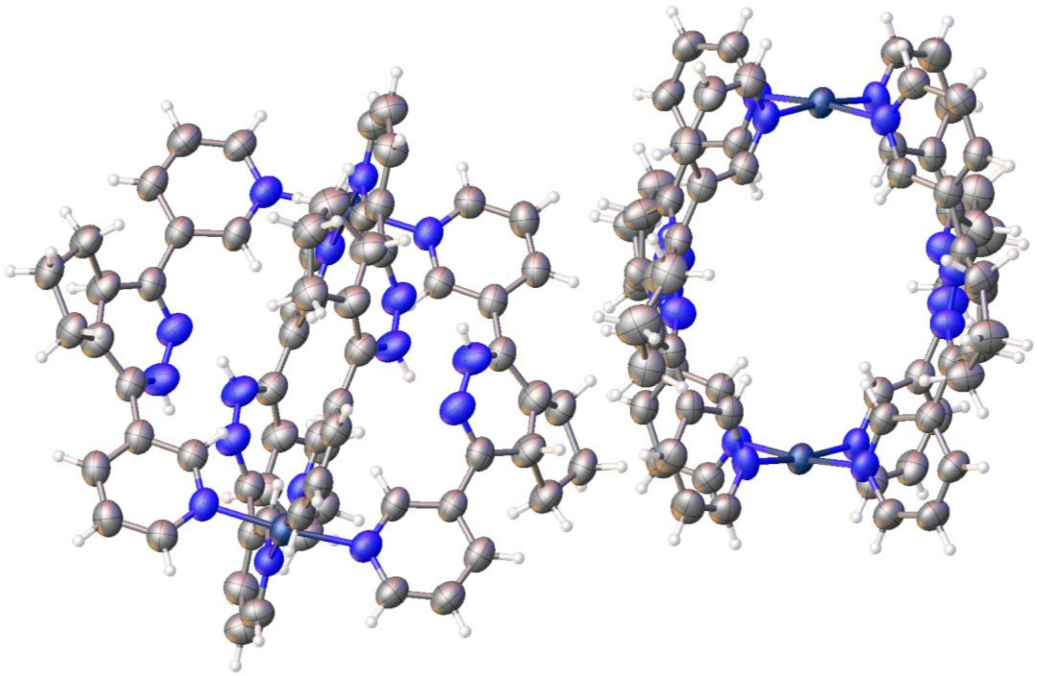

Figure S78: Image of lantern cage **3''** at 50% ellipsoids

S10.7 Single Crystal Structure of 3''T

Single crystals of lantern cage **3''T** ($C_{152}H_{144}B_8F_{32}N_{40}Pd_4$) were grown via vapour diffusion of diisopropyl ether into MeCN while in a dark environment. A suitable crystal was selected and mounted in fomblin film on a XtalLAB PRO MM007, PILATUS3 R 200K diffractometer. The crystal was kept at 120(2) K during data collection. Using Olex2,[7] the structure was solved with the SHELXT[8] structure solution program using Intrinsic Phasing and refined with the SHELXL[9] refinement package using Least Squares minimisation.

S10.7.1 Specific crystal structure and refinement details for tetrahedral cage 3''T

The structure of the large metallo-coordination cage complex was difficult to refine owing to the overall porosity of the structure and the intrinsic disorder of the constituent ligands, anions and solvent residues. These factors contribute to weak scattering and a low angle diffraction limit despite the examination of many crystals and use of a high intensity rotating anode X-ray source. The data used in the refinement was truncated to a resolution of 0.92 Å. The solution from which the crystals were grown contained a mixture of isotopical bidentate bridging ligands in either their oxidised (pyridazine) or reduced (dihydropyridazine) form between the pyridyl donor moieties. The oxidative state of the ligands dictates subtle changes in the shape of the ligand; the oxidised form has a 2-fold symmetric planar aromatic ring whilst the reduced form possesses a non-planar asymmetric chiral conformation and an N-H group at one of the nitrogen atoms. The two forms of the ligand orient their pyridyl donor moieties similarly enough that they are interchangeable with each other in the coordination cage complex. The asymmetric reduced form of the ligand can fulfil the bridging role in one of two orientations in which the positions of the N-H amine and C-H methine groups are swapped over. The ligands can also exhibit disorder in the conformations of their cyclopentyl ring moieties and the rotation of the pyridazine/dihydropyridazine rings about the pyridyl-pyridyl axis. Owing to problems associated with the data quality and ligand disorder stated above, the inferences that can be drawn from this structure are limited to the following key points. The structure is a cage complex composed of four palladium cations and eight ligands with the cations and bridging ligands adopting a tetrahedral topology. Where two ligands traverse the same edge of this tetrahedron their mutual orientation and conformations are consistent with at least one of the ligands donating an N-H···N hydrogen bond to the other. The chemical identity of any specific ligand cannot be specified; indeed the severity of the disorder at the pyridazine/dihydropyridazine linker moieties is entirely consistent with the types of disorder discussed in this experimental section. No detailed bond lengths or angles can be deduced from this dataset.

The four palladium cations are refined with anisotropic displacement parameters. All other atoms in the structure are refined with isotropic displacement parameters. Similarity restraints were applied to the atomic displacement parameters of all atoms in the structure (SIMU). Disorder is observed in varying degrees in all the ligand pyridazine/dihydropyridazine linker moieties. The nature and severity of the disorder is consistent with the presence of a mixture of ligands scrambled positionally and conformationally in the manners described above. The mode of bridging between Pd(II) cations by the ligands can be categorised as either single-walled edge or double-walled edge; four of each ligand are found in the structure. The four ligands on the double-walled edges (A-D) are observed at full occupancy in a single rotational conformation. The four ligands found on the single-walled edges (E-H) are rotationally disordered around the pyridyl-pyridyl axis. In ligands E, F, and H each one of the disorder components is modelled at a fixed half occupancy. In ligand G the three carbon atoms of the cyclopentyl ring moiety could not be identified in the electron density map or modelled. In the case of ligand G, the assignment of the two nitrogen atoms of the heterocyclic ring was made based on steric factors dictating the likely orientation with respect to the cage cavity.

Given the weak data, extensive use of restraints was necessary to aid refinement of a sensible model. The choice of geometric restraints was complicated by the underlying disorder of the pyridazine/dihydropyridazine rings, meaning that many chemically differing moieties are overlaid.

The geometries of all pyridyl rings were restrained to be planar (FLAT) and similar (SAME). The connecting atoms of all pyridyl and pyridazine/dihydropyridazine rings for single-walled edge ligands (E-H) were restrained to be coplanar with the rings to which they are connected (FLAT). The bond distances between connecting atoms of pyridyl and pyridazine/dihydropyridazine for all ligands were restrained to be similar (SADI). The pyridazine/dihydropyridazine ring moieties of all single-walled edge ligands (E-H) were restrained to be planar (FLAT) and to have similar 1,2 bond distances around the ring (SADI). These last two restraints contradict the expected asymmetric geometry for dihydropyridazine rings, however, they are deemed an acceptable compromise given the need to apply approximately appropriate restraints to a very disordered structure. The 1,2 and 1,3 distances around the cyclopentyl rings of all ligands were restrained to be similar (SADI).

Of a total of eight tetrafluoroborate anions expected based on charge balance, four sites were identified in the electron density map. The occupancies of the tetrafluoroborate anions were refined before being fixed at indicated partial or full occupancies of 1, 0.5, 0.5, and 0.75. The geometries of the tetrafluoroborate anions were constrained to refine as rigid bodies with coordinates taken from the Olex2 FragmentDB.

Where ligand fragments are modelled at partial occupancy or omitted from the model they have been included in the unit cell contents at full occupancy. The formula unit used to calculate this content is based on the presence of four pyridazine ligands and four dihydropyridazine ligands. This assignment is consistent with the structure refined and supporting analysis, however the data presented does not discount the presence of different formula units co-crystallised together.

No hydrogen atoms were observed in the electron density map. Hydrogen atoms for pyridyl sp^2 carbon atoms and cyclopentyl methylene carbon atoms were geometrically placed and refined with a riding model. Potential hydrogen atoms for the disordered dihydropyridazine carbon and nitrogen atoms were not included in the model, however, they were included in the unit cell contents according to the proposed formula unit.

Significant residual electron density peaks (range 1.78 - 2.67 e $Å^{-3}$) are observed on all palladium atoms in the structure. The identity of the atoms as Pd is confirmed by mass spectrometry. The residual electron density and further residual peaks along the pyridyl-Pd bond coordinates are likely a consequence of a minor unmodelled disorder component potentially involving the position of the cation. Although these peaks are significant, they do not undermine the conservative inferences about the structure discussed above.

A solvent mask was calculated, and 1623 electrons were found in a volume of 7480 $Å^3$ in 1 void per unit cell. This is consistent with the presence of 5.25[BF_4], 8[C_2H_3N] and [$C_{11.5}H_{22}N_4$] per formula unit, which accounts for 1614 electrons per unit cell. The missing disorder components and hydrogen atoms from the pyridazine/dihydropyridazine ligands are accounted for in the moiety unit [$C_{11.5}H_{22}N_4$].

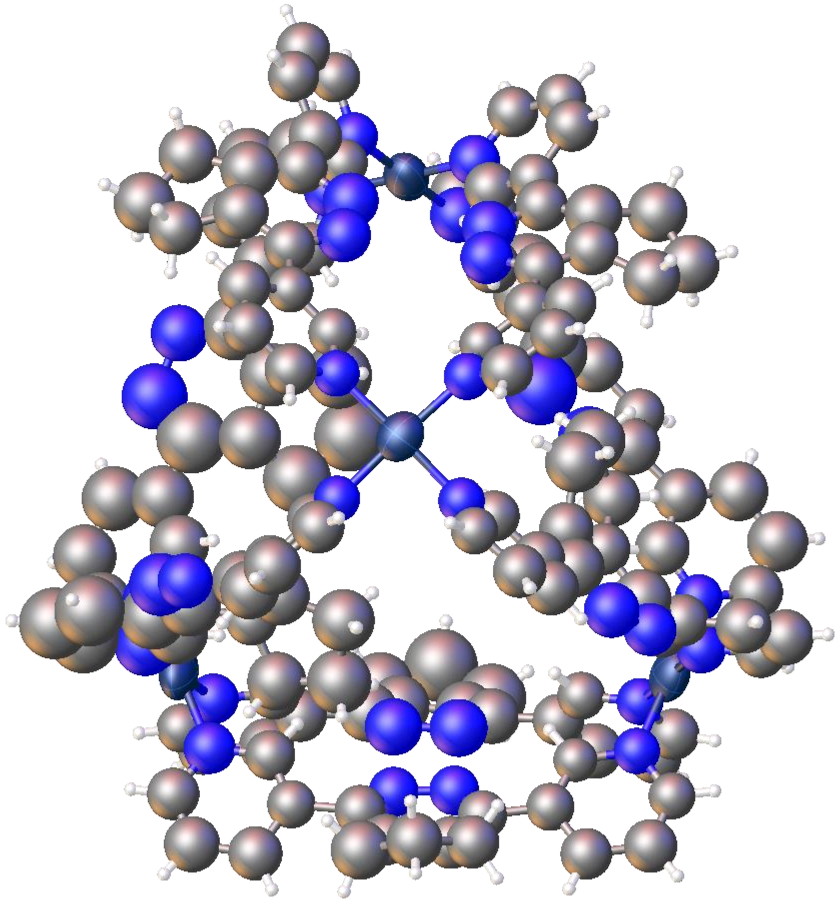

Figure S79: Image of **3''T** at 50% ellipsoids

S10.8 Single Crystal Structure of 4

Single crystals of tetrahedral cage **4** ($C_{156}H_{142}B_8F_{32}N_{42}Pd_4$) were grown via vapour diffusion of diisopropyl ether into MeCN. A suitable crystal was selected and mounted in fomblin film on a micromount on a Fluid Film Devices diffractometer. The crystal was kept at 100(2) K during data collection. Using Olex2,[7] the structure was solved with the SHELXT[8] structure solution program using Intrinsic Phasing and refined with the SHELXL[9] refinement package using Least Squares minimisation.

S10.8.1 Specific crystal structure and refinement details for tetrahedral cage 4

The small needle-like crystals of a supramolecular metallo-cage complex were weakly diffracting with a low-resolution diffraction limit despite the use of synchrotron radiation. The data used in the refinement was truncated to a resolution of 1.2 Å. Measures taken to aid refinement of the structure against a low data parameter ratio include extensive use of restraints and constraints to the atom geometries and thermal displacement parameters.

All atoms in the structure except for the palladium were refined with isotropic displacement parameters; the weak low-resolution data did not support meaningful refinement of anisotropic ellipsoids for the displacement of the light atoms, even with the use of restraints. Similarity restraints were applied to the isotropic displacement parameters of all atoms in the structure.

Geometric similarity restraints were applied to the geometries of all pyridyl rings and 6,7-dihydro-5H-cyclopentapyridazine moieties, including the bonds between these moieties (SAME, SADI). The aromatic ring portions of these moieties were restrained to have planar geometries (FLAT). The geometries of selected C–N, C–C and 1,3 C⋯C distances within the pyridyl moieties were further restrained to target distances from similar structures in the CCDC. The 6,7-dihydro-5H-cyclopentapyridazine moieties of ligands C and D are disordered over two conformations which have been modelled at a fixed occupancy of 50% in each case.

Out of the expected eight tetrafluoroborate anions expected to balance the charge of the four Pd(II) cations, five were observed in electron density map. The anions were refined as rigid bodies with geometries taken from the Olex2 FragmentDB tool. The occupancies of two of the anions were fixed at values of 0.75 and the occupancy of one was fixed at 0.5.

Pairs of large residual electron density peaks (range 1.46-2.12 e $Å^{-3}$) are observed on opposite sides of all four Pd atoms, approximately aligned along the unit cell c-direction (distances in the range 1.30-1.69 Å). It is likely these peaks are a result of deficiencies in the absorption correction or other systematic errors in the processing of the weak low resolution diffraction data.

Large regions of diffuse solvent and anion residues were treated with the Olex2 Solvent mask tool. The assignment of the masked residues was informed by the assumption of charge balance and the atomic volume of solvent likely to fill the remainder of the void.

A solvent mask was calculated, and 2776 electrons were found in a volume of 268 Å^3 in 3 voids per unit cell. This is consistent with the presence of four BF_4^- anions and 10 acetonitrile residues per formula unit, which accounts for 3072 electrons per unit cell.

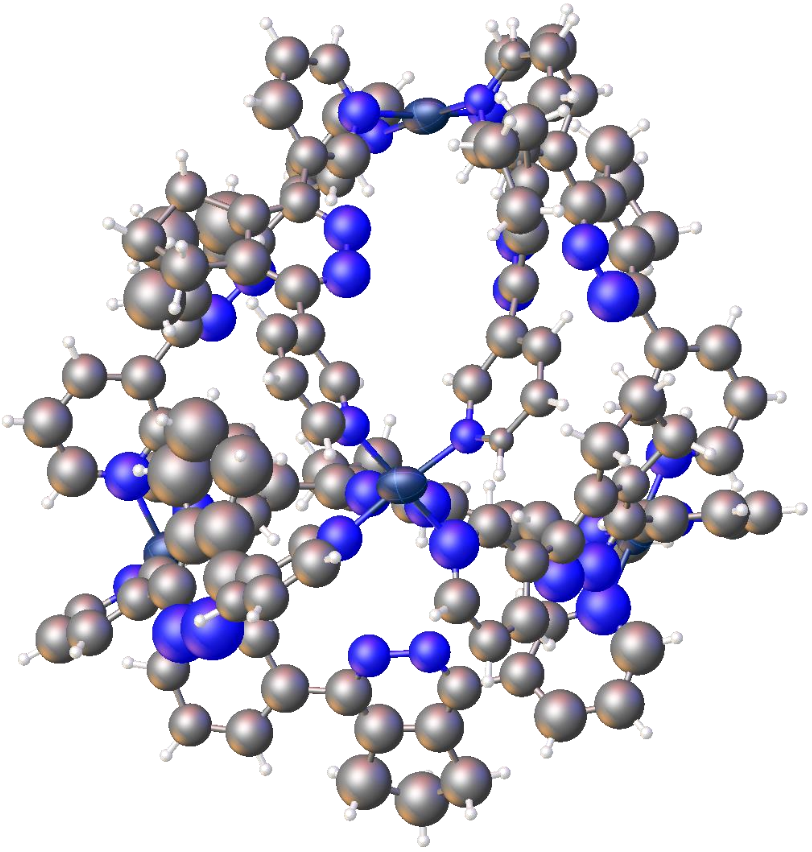

Figure S80: Image of tetrahedral cage **4** at 50% ellipsoids

S11 References

(1) Li, C.; Ge, H.; Yin, B.; She, M.; Liu, P.; Li, X.; Li, J. Novel 3,6-unsymmetrically disubstituted-1,2,4,5-tetrazines: S-induced one-pot synthesis, properties and theoretical study. *RSC Adv.* **2015**, *5*, 12277.

(2) Oxtoby, N. S.; Blake, A. J.; Champness, N. R.; Wilson, C. The role of 1,2,4,5-tetrazine rings in π–π stacking interactions. *CrystEngComm* **2003**, *5*, 82.

(3) Frisch, M. J.; Trucks, G. W.; Schlegel, H. B.; Scuseria, G. E.; Robb, M. A.; Cheeseman, J. R.; Scalmani, G.; Barone, V.; Mennucci, B.; Petersson, G. A.; Nakatsuji, H.; Caricato, M.; Li, X.; Hratchian, H. P.; Izmaylov, A. F.; Bloino, J.; Zheng, G.; Sonnenberg, J. L.; Hada, M.; Ehara, M.; Toyota, K.; Fukuda, R.; Hasegawa, J.; Ishida, M.; Nakajima, T.; Honda, Y.; Kitao, O.; Nakai, H.; Vreven, T.; Montgomery, J. A.; Peralta, J. E.; Ogliaro, F.; Bearpark, M.; Heyd, J. J.; Brothers, E.; Kudin, K. N.; Staroverov, V. N.; Kobayashi, R.; Normand, J.; Raghavachari, K.; Rendell, A.; Burant, J. C.; Iyengar, S. S.; Tomasi, J.; Cossi, M.; Rega, N.; Millam, J. M.; Klene, M.; Knox, J. E.; Cross, J. B.; Bakken, V.; Adamo, C.; Jaramillo, J.; Gomperts, R.; Stratmann, R. E.; Yazyev, O.; Austin, A. J.; Cammi, R.; Pomelli, C.; Ochterski, J. W.; Martin, R. L.; Morokuma, K.; Zakrzewski, V. G.; Voth, G. A.; Salvador, P.; Dannenberg, J. J.; Dapprich, S.; Daniels, A. D.; Farkas; Foresman, J. B.; Ortiz, J. V.; Cioslowski, J.; Fox, D. J. Wallingford CT, **2009**.

(4) Birvé, A. P.; Patel, H. D.; Price, J. R.; Bloch, W. M.; Fallon, T. Guest-Dependent Isomer Convergence of a Permanently Fluxional Coordination Cage. *Angew. Chem., Int. Ed.* **2022**, *61*, e202115468.

(5) Cosier, J. t.; Glazer, A. A nitrogen-gas-stream cryostat for general X-ray diffraction studies. *J. Appl. Crystallogr.* **1986**, *19*, 105.

(6) Rigaku Oxford Diffraction, (2018), CrysAlisPro Software system, version 1.171.40.45a, Rigaku Corporation, Oxford, UK.

(7) Dolomanov, O. V.; Bourhis, L. J.; Gildea, R. J.; Howard, J. A.; Puschmann, H. OLEX2: a complete structure solution, refinement and analysis program. *J. Appl. Crystallogr.* **2009**, *42*, 339.

(8) Sheldrick, G. SHELXT - Integrated space-group and crystal-structure determination. *Acta Cryst. A* **2015**, *71*, 3.

(9) Sheldrick, G. Crystal structure refinement with SHELXL. *Acta Cryst. C* **2015**, *71*, 3

(10) "CheckCIF," can be found under http://checkcif.iucr.org.

(11) (a) Winter, G. xia2: an expert system for macromolecular crystallography data reduction. *J. Appl. Crystallogr.* **2010**, *43*, 186; (b) Allan, D. R.; Nowell, H.; Barnett, S. A.; Warren, M. R.; Wilcox, A.; Christensen, J.; Saunders, L. K.; Peach, A.; Hooper, M. T.; Zaja, L. A novel dual air-bearing fixed-χ diffractometer for small-molecule single-crystal X-ray diffraction on beamline I19 at diamond light source. *Crystals* **2017**, *7*, 336; (c) Winter, G.; Waterman, D. G.; Parkhurst, J. M.; Brewster, A. S.; Gildea, R. J.; Gerstel, M.; Fuentes-Montero, L.; Vollmar, M.; Michels-Clark, T.; Young, I. D. DIALS: implementation and evaluation of a new integration package. *Acta Cryst. D* **2018**, *74*, 85; (d) Beilsten-Edmands, J.; Winter, G.; Gildea, R.; Parkhurst, J.; Waterman, D.; Evans, G. Scaling diffraction data in the DIALS software package: algorithms and new approaches for multi-crystal scaling. *Acta Cryst. D* **2020**, *76*, 385.

(12) Journal, 2018, CCP4 7.0.062: AIMLESS, version 060.067.062 : 027/005/018.
